# Bone in Parathyroid Diseases Revisited: Evidence From Epidemiological, Surgical and New Drug Outcomes

**DOI:** 10.1210/endrev/bnaf010

**Published:** 2025-04-03

**Authors:** Afroditi Roumpou, Andrea Palermo, Symeon Tournis, Valeria Hasenmajer, Janice L Pasieka, Gregory Kaltsas, Andrea Isidori, Eva Kassi

**Affiliations:** Endocrine and Bone Metabolic Disorders Unit, Second Propaedeutic Department of Internal Medicine, Attikon University Hospital, School of Medicine, National and Kapodistrian University of Athens, Athens 12462, Greece; Unit of Metabolic Bone and Thyroid Diseases, Fondazione Policlinico Universitario Campus Bio-Medico, Rome 00128, Italy; Unit of Endocrinology and Diabetes, Campus Bio-Medico University of Rome, Rome 00128, Italy; Laboratory for Research of the Musculoskeletal System “Th. Garofalidis”, Medical School, National and Kapodistrian University of Athens, KAT General Hospital, Kifissia 14561, Athens, Greece; Department of Experimental Medicine Sapienza, Endocrine and Andrological Regional, University of Rome, Rome 00185, Italy; Rare Disease Center (Endo-ERN accredited), Policlinico Umberto I, Rome 00161, Italy, European Reference Network on Rare Endocrine Conditions (ENDO-ERN); Departments of Surgery and Oncology, University of Calgary, Calgary, Canada AB T2N 2T9; Division of Diabetes, Endocrinology and Metabolic Diseases, First Department of Propaedeutic and Internal Medicine, General Hospital of Athens LAIKO, School of Medicine, National and Kapodistrian University of Athens, Athens 11527, Greece; Center of Expertise for Rare Endocrine Diseases (ENDO-ERN accredited), General Hospital of Athens LAIKO, National and Kapodistrian University of Athens, Athens 11527, Greece, European Reference Network on Rare Endocrine Conditions (ENDO-ERN); Department of Experimental Medicine Sapienza, Endocrine and Andrological Regional, University of Rome, Rome 00185, Italy; Rare Disease Center (Endo-ERN accredited), Policlinico Umberto I, Rome 00161, Italy, European Reference Network on Rare Endocrine Conditions (ENDO-ERN); Division of Diabetes, Endocrinology and Metabolic Diseases, First Department of Propaedeutic and Internal Medicine, General Hospital of Athens LAIKO, School of Medicine, National and Kapodistrian University of Athens, Athens 11527, Greece; Center of Expertise for Rare Endocrine Diseases (ENDO-ERN accredited), General Hospital of Athens LAIKO, National and Kapodistrian University of Athens, Athens 11527, Greece, European Reference Network on Rare Endocrine Conditions (ENDO-ERN); Department of Biogolical Chemistry, School of Medicine, National and Kapodistrian University of Athens, Athens 11527, Greece

**Keywords:** bone, fracture, hyperparathyroidism, hypoparathyroidism, parathyroid disorders, pseudohypoparathyroidism

## Abstract

PTH-related disorders have a major impact on bone metabolism and skeletal properties because of the pivotal role of PTH in calcium and phosphate homeostasis and bone remodeling. Hyperparathyroidism is characterized by continuous exposure to excessive endogenous PTH, causing increased bone turnover in favor of bone resorption. Depending on the background of PTH overproduction, hyperparathyroidism is divided into primary, secondary, and tertiary hyperparathyroidism. The clinical presentation varies from deterioration of bone microarchitecture and decreased bone mineral density to profound bone involvement, such as osteitis fibrosa cystica and fragility fractures. Although successful parathyroidectomy represents the definitive treatment and may promote regression of most of the skeletal defects, the medical approach of calcimimetics and antiresorptive agents is a promising alternative in cases where parathyroidectomy is not feasible or unsuccessful. Hypoparathyroidism is the pathophysiological counterpart of hyperparathyroidism and also leads to disorders of bone metabolism and structure. Chronic PTH deprivation is associated with low bone remodeling and increased bone mineral density. The defective microarchitecture might affect bone strength and raise the risk for adverse skeletal events. Recombinant human PTH acts as a replacement therapy and is safe and efficient in restoring calcium/phosphate homeostasis and bone turnover. However, it is approved only for refractory cases, as conventional management with calcium and active vitamin D remains the first-line treatment. This article reviews the skeletal involvement in the most frequent parathyroid disorders, hyperparathyroidism and hypoparathyroidism, and rare familial disorders of PTH metabolism, as assessed by clinical, laboratory, and imaging parameters, and the effect of the available treatment strategies.

## Graphical Abstract

**Graphical Abstract:**
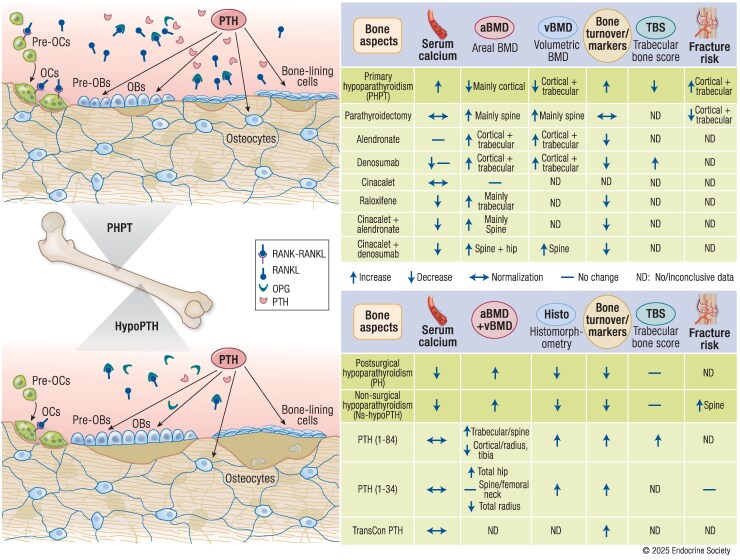

**Essential Points**

- Primary hyperparathyroidism leads to increased bone remodeling, impaired mineralization, decreased cortical and trabecular BMD, and increased fracture risk due to continuous exposure of the skeleton to high PTH levels.
- Successful parathyroidectomy remains the mainstay for treatment of primary hyperparathyroidism leading to restoration of BMD and fracture risk reduction, although antiresorptive agents (ie, bisphosphonates, denosumab) can decrease bone resorption and restore to an extent BMD.
- Chronic kidney disease includes a wide range of skeletal manifestations, ranging from hyperparathyroidism with high bone turnover to adynamic bone disease with low bone turnover or mixed disorder.
- Cinacalcet has proved its efficacy in BMD improvement and fracture risk reduction in chronic kidney disease-mineral and bone disorder, whereas antiresorptive drugs and romosozumab appear to improve bone loss when selected in case-by-case basis.
- Hypoparathyroidism leads to elevated BMD and impaired bone microarchitecture; however, data on bone strength and fracture risk remain controversial, with a trend toward increased risk for vertebral fractures in nonsurgical hypoparathyroidism.
- Treatment of hypoparathyroidism with human recombinant PTH (1-84) is associated with bone remodeling restoration, whereas Trans-Con PTH demonstrates promising effects in terms of calcium normalization and restoration of BMD; however, data on their effects on fracture risk are missing.
- Pseudohypoparathyroidism is a rare disorder caused by resistance of target tissues to PTH, and because there are no data on the prevalence of osteoporosis in patients with pseudohypoparathyroidism, treatment should be aimed at maintenance of serum PTH and calcium within normal levels to normalize bone metabolism.

Parathyroid disorders are responsible for a wide range of manifestations and skeletal abnormalities, that result from alterations of bone metabolism. Nowadays, the measurement of serum calcium and PTH has become increasingly accessible. Therefore, most parathyroid disorders are diagnosed incidentally, early in the course of the disease, when skeletal involvement is mild or even asymptomatic. Primary hyperparathyroidism (PHPT) is the most common parathyroid disorder due to the autonomous overproduction of PTH mostly from parathyroid adenomas, with significant skeletal consequences driven by increased bone turnover, although the classic and extreme manifestation of PHPT*, osteitis fibrosa cystica,* is now rarely seen. Unlike PHPT, in secondary hyperparathyroidism (SHPT), excessive PTH is not autonomously produced by parathyroid glands, but most commonly results from defects in calcium homeostasis that lead to hypocalcemia. On the other hand, hypoparathyroidism (HypoPTH) is characterized by absent or inappropriately low PTH levels in the presence of hypocalcemia, and it mainly occurs as a neck surgery complication. PTH deficiency leads to reduced bone turnover and subsequent rise of total volumetric bone mineral density (BMD). Despite the elevated BMD, bone disease results from increased bone mineralization and altered skeletal microarchitecture. Here, we review the impact of the main parathyroid disorders on bone, evaluating bone remodeling, bone strength, and fracture risk. Then, we analyze the mechanisms by which the available treatments for these diseases affect the skeleton.

## The Role of PTH in Bone and Mineral Metabolism

**Figure 1.**
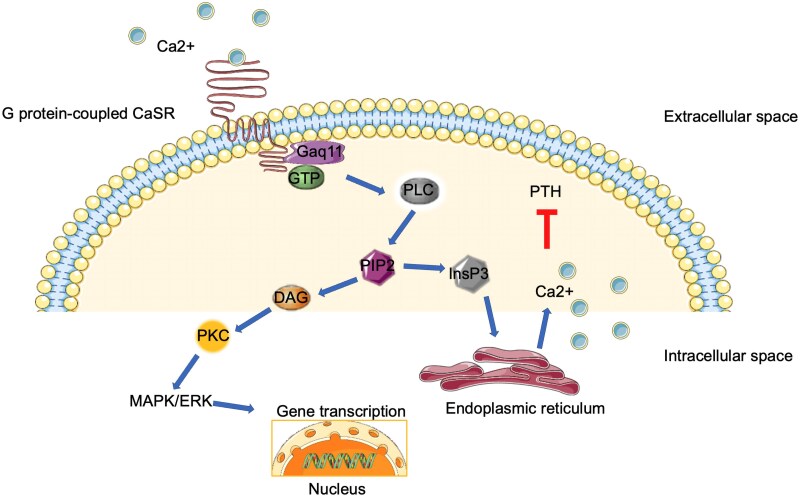
The calcium-sensing receptor (CaSR) signaling in the parathyroid cell. CaSR is a G-coupled protein receptor consisting of an extracellular domain, an intramembrane domain of seven hydrophobic helices, and an intracellular domain. The large extracellular domain interacts with the extracellular ionized calcium (Ca^2+^) leading to activation of the G-protein and in turn of the phospholipase C (PLC). PLC hydrolyses phosphatidylinositol-4,5-diphosphate (PIP2) to inositol-14,5-triphosphate (InsP3) and diacylglycerol (DAG). InsP3 mediates the release of intracellular calcium from the endoplasmic reticulum, resulting in inhibition of PTH secretion. DAG activates protein kinase C (PKC) resulting in gene transcription in the nucleus by activating mitogen-activated protein kinase (MAPK) and extracellular signal-regulated kinases (ERK).

**Figure 2.**
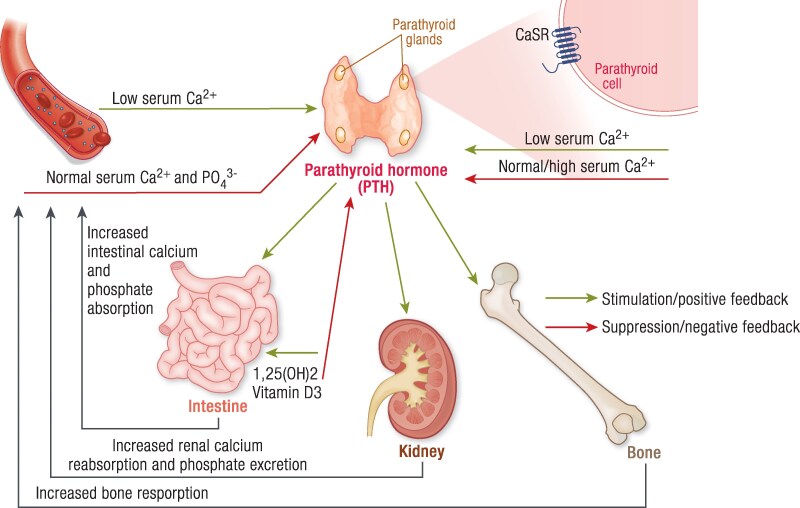
Parathyroid hormone (PTH) is a main regulator of calcium and phosphate metabolism. Calcium levels (Ca^2+^) are tightly controlled through the action of PTH and 1,25(OH)_2_D_3_ (calcitriol). Calcium-sensing receptors (CaSRs) are localized on the parathyroid cell membrane and detect changes in the serum Ca^2+^ concentrations. Hypocalcemia triggers the release of PTH by the parathyroid cells; conversely, hypercalcemia suppresses the release of PTH. PTH stimulates bone resorption, which increases serum calcium and phosphate (PO_4_^3−^) levels. In the kidney, PTH stimulates the reabsorption of calcium and promotes phosphate excretion. PTH promotes the conversion of 25-hydroxyvitamin D (25(OH)D) to calcitriol in the kidney, the active form of vitamin D responsible for increased intestinal absorption of calcium and phosphate. All these well-orchestrated steps restore calcium levels to the normal range (8.5-10.5 mg/dL) and via actions of PTH and other hormones, such as fibroblast growth factor 23, in the kidney, restore the phosphate levels within the normal range (2.5-4.5 mg/dL).

PTH is a key regulator of calcium homeostasis and of bone metabolism. Parathyroid glands synthesize PTH as a precursor peptide and then store it as a cleaved bioactive molecule (PTH 1-84). Parathyroid cells have the capacity to sense minute fluctuations of extracellular calcium, through calcium-sensing receptor (CaSR). CaSR is a G protein-coupled receptor that mediates signaling through activation of phospholipase C and subsequent generation of diacylglycerol and inositol triphosphate (1). The signaling pathway results in increase in the intracellular calcium and inhibition of PTH release (Fig. 1). PTH is excreted in response to low serum ionized calcium levels (Ca^2+^), whereas PTH gene transcription and stability are inhibited by increased Ca^2+^ (2, 3). Chronic hypocalcemia promotes cellular replication leading to hyperplasia of the parathyroid glands. PTH actions aim to restore serum calcium levels, directly affecting bone and kidney mineral metabolism and indirectly affecting intestinal mineral absorption (Fig. 2). In normal state, all these interactions strictly maintain Ca^2+^ levels within a narrow range (1.1-1.3 mmol/L) (4). Precise homeostasis of Ca^2+^ is requisite, not only for bone metabolism, but also for many other physiological processes, such as cell signaling and neuromuscular function.

Despite the fact that the main circulating bioactive form of PTH is an 84 amino acid peptide, almost its entire biological activity is contained within the amino (NH_2_)-terminal domain, and therefore, the synthetic peptide composed of the NH_2_-terminal 34 residues (PTH 1-34, teriparatide) appears to exhibit the same biological activity of the entire molecule (5). PTH actions are mediated by a G protein-coupled receptor, the PTH/PTH-related protein (PTHrP) receptor 1 (PTH1R). Its name implies the signaling transduction of the PTHrP as well, which is a relative molecule of PTH (6). PTH and PTHrP can both act on the same receptor because of their structural resemblance in the amino-terminal domain, which allows them to form similar secondary/tertiary structures when bound to the receptor (6). However, the mediated biological effects are not identical. It appears that PTHrP can exert biological activities through domains beyond the amino-terminal, which remains to be fully explored (7). PTH or PTHrP binding to PTH1R leads to activation of G proteins, mainly Gas, which in turn activates adenylyl cyclase, that produces cAMP as a second messenger. The signaling cascade results in activation of protein kinase A (PKA), which translocates to the nucleus and phosphorylates cAMP-responsive elements in DNA, enabling transcription of target genes (8). This signal transduction is controlled by multiple intracellular mechanisms (cAMP degradation by phosphodiesterases, selective activation of PKA, inhibition of the activity of PKA by PKA inhibitor peptide) (9-11). Interestingly, the lesser bioavailability of PTHrP to PTH1R attributes to the reduced potency of PTHrP compared to the PTH peptides as osteoanabolic drugs (7).

PTH can have catabolic or anabolic effect on bone (12). It stimulates bone resorption through OPG-RANKL-RANK pathway (osteoprotegerin-receptor activator of nuclear factor κB ligand-receptor activator of nuclear factor κB). In particular, PTH modulates the expression of RANKL and OPG both in the precursors of osteoblasts (mesenchymal stem cells) and in osteocytes; the first binds to RANK on the surface of hemopoietic precursors of osteoclasts and of mature osteoclasts, promoting their differentiation, survival, and activity; the second binds to RANKL, inhibiting the RANK-RANKL interaction (13-16). The balance between the actions of these factors determines osteoclastogenesis (17). Mice studies have shown that osteocytes are the major source of RANKL resulting in PTH-mediated bone resorption (18-20). In mice and rats, continuous infusion of PTH increases mRNA encoding for RANKL and decreases mRNA encoding for OPG, resulting in an increased RANKL/OPG ratio, which in turn promotes osteoclastogenesis and bone resorption (21-23). In humans, it seems that PTH regulates the OPG-RANKL-RANK pathway accordingly. Individuals with primary hyperparathyroidism have higher levels of RANKL, OPG, and RANKL/OPG ratio than healthy controls (24), and the high levels of RANKL are favorably associated with bone resorption indicators and rates of bone loss at the total femur (25), whereas it has been shown that 1 year after parathyroidectomy (PTX), RANKL/OPG ratio drops (26). Another potential mediator of PTH-induced bone resorption is the monocyte chemoattractant protein-1 (MCP-1), a chemokine for monocytes and macrophages. In vitro and in vivo studies suggest that MCP-1 promotes chemoattraction of osteoclast precursors and stimulates RANKL-induced osteoclastogenesis, which in turn increases bone resorption, whereas PTH treatment enhances MCP-1 expression in rats (27). In addition, MCP-1 levels have been positively associated with PTH levels in primary hyperparathyroidism patients and have been reported to be markedly and promptly decreased after PTX (28). PTH-mediated bone resorption seems to particularly affect the cortical bone (29). Prolonged exposure to excessive levels of PTH promotes bone remodeling and demineralization of the skeleton, reduces BMD and increases the risk of all osteoporotic fractures (30).

The significant anabolic role of PTH in normal fetal bone formation as well as in normal fracture healing has been documented in studies on mice models (31, 32). Preclinical studies have also demonstrated that, contrary to the continuous exposure to endogenous PTH, intermittent administration of exogenous PTH exerts significant anabolic effects on bone (33, 34). The clinical application of this observation has led to the approval of PTH1R agonists (teriparatide and abaloparatide), as osteoanabolic therapeutic agents for osteoporosis (35). Although treatment with intermittent PTH causes an increase in bone turnover, such as continuous PTH exposure, it leads to an early promotion of bone production without resorption, followed by a subsequent general increase in bone turnover. The “anabolic window” refers to the time frame during which PTH exerts its most anabolic effects (36-38). Intermittent PTH administration promotes osteoblast differentiation and increases the number of osteoblasts by inhibiting their apoptosis (36, 39). Through PTH1R signaling, cAMP/PKA stimulation and subsequent phosphorylation of pro-apoptotic proteins, PTH (1-34) administration quickly triggers anti-apoptotic signaling pathways (40), leading to delayed osteoblast apoptosis (41). The anabolic effect of PTH on osteoblast lineage is mediated through the expression of genes that normally signal bone formation (such as osteoblast-specific transcription factor Runx2, osteocalcin, alkaline phosphatase) as well as through bone morphogenetic protein signaling (42, 43). Moreover, the glycoprotein sclerostin, mainly derived from osteocytes, inhibits bone formation (44). Its expression and action is blocked by PTH, resulting in increased bone modeling (45). Levels of sclerostin were lower in patients with primary hyperparathyroidism compared to controls, having a negative correlation with PTH levels and being increased after PTX (46-48). Similar to sclerostin, Dickkopf1 (DKK-1) is a protein secreted by osteoblasts and inhibits Wnt signaling and bone formation. Preclinical and clinical studies provide controversial results regarding the effect of PTH on DKK-1 (49, 50), providing space for further research regarding the role of DKK-1 as a mediator of PTH-related bone formation.

The equilibrium between the new bone formation vs bone resorption determines the total net effect of PTH on bone remodeling. When Ca^2+^ in the extracellular space is decreased, PTH secretion responds immediately toward maintaining normal calcium levels by increasing bone resorption more than formation. On the other hand, PTH deficiency results in low bone remodeling, leading to increased BMD, whereas data regarding fracture risk are still unclear (12, 30) (Table 1). Overall, these features confirm that PTH plays a pivotal role in regulating bone remodeling. The bone abnormalities caused by endogenous PTH alterations in the context of common parathyroid disorders are detailed later.

**Table 1.** The effect of endogenous PTH excess and deficiency in skeletal properties

Endogenous PTH	aBMD (DXA)	vBMD (qCT)	TBS	Bone remodeling	Fracture risk
Excess	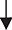	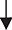	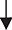	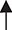	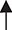
Deficiency	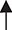	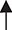	=	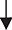	=, 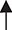 *^a^*

Abbreviations: 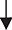, decrease; 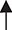, increase; =, no changes; aBMD, areal bone mineral density; DXA, dual-energy X-ray absorptiometry; PTH, parathyroid hormone; qCT, quantitative computed tomography; TBS, trabecular bone score; vBMD, volumetric bone mineral density.

^a^The increased risk of vertebral fractures concerns only patients with nonsurgical hypoparathyroidism.

## Hyperparathyroidism

### Primary Hyperparathyroidism

PHPT is characterized by excessive and inappropriate secretion of endogenous PTH by 1 or more parathyroid glands, resulting in hypercalcemia because of a concomitant defective feedback control of PTH secretion by extracellular calcium concentrations (4). The incidence of PHPT varies from 34 to 120 cases per 100 000 people, positively correlated with age (51), with the majority of cases being postmenopausal women (52, 53). It has been speculated that estrogen acts as a regulator for PTH's effect on bone by inhibiting bone resorption, and that, during menopause, the state of estrogen insufficiency eliminates this regulation, enabling PTH to fully affect bone, resulting in hypercalcemia (54). The favorable effect of estrogen and raloxifene in controlling bone turnover and biochemical indices of PHPT (55, 56) lends credence to this theory.

PHPT is the leading cause of hypercalcemia in the outpatient environment (3, 57). Although the majority of sporadic PHPT cases (75%-85%) are caused by a single benign adenoma, 15% to 25% of cases are associated with multigland adenoma or hyperplasia. Approximately 5% to 10% of PHPT cases are due to familial isolated or syndromic diseases, with multiple endocrine neoplasia (MEN) 1 being the most common type (58, 59). Unlike sporadic forms, syndromic and hereditary PHPT are often associated with multiple parathyroid tumors/hyperplasia. The group of syndromic PHPT constitutes MEN types 1, 2A, and 4, familial isolated hyperparathyroidism, and the hyperparathyroidism-jaw tumor syndrome (HPT-JT). The latter is associated with parathyroid cancer in approximately 15% of cases (58). Parathyroid cancer represents a very small percentage of all PHPT cases (<1%) (4).

The initial diagnostic workup of hyperparathyroidism should be focused on excluding causes of SHPT (eg, kidney failure, vitamin D deficiency, treatment with diuretics) (4). Biochemical evaluation is needed to confirm the diagnosis of PHPT. However, the disorder may appear with a vast range of biochemical profiles, thus complicating the diagnosis. The classic biochemical presentation of PHPT is characterized by elevated serum calcium levels along with inappropriately increased PTH levels. Elevated serum calcium levels and inappropriately normal PTH levels are characteristics of the variant known as normohormonal PHPT (up to 20% of the cases). A third variant is normocalcemic PHPT (NPHPT), which is defined by persistently elevated serum PTH levels and normal total and ionized calcium levels, in the absence of secondary causes. Classic hypercalcemic PHPT (HPHPT) may follow NPHPT, as a number of these patients may develop hypercalcemia over time (57). It is crucial to differentiate PHPT from the genetic disorder of familial hypocalciuric hypercalcemia (FHH), as biochemical tests, such as serum calcium, PTH, and phosphate levels often overlap, whereas management approaches differ significantly; the gold standard in the management of PHPT is surgery, in opposition to FHH, where PTX fails to lower calcium levels and generally is contraindicated (60). Calcium excretion is usually elevated in PHPT and may predispose in nephrolithiasis. Calcium-creatinine clearance ratio is used to distinguish the 2 disorders, with the majority of cases with FHH and PHPT having a clearance ratio of <0.01 and >0.02, respectively. Between 0.01 and 0.02 can be found in approximately 40% of people with either condition (60, 61).

The clinical presentation of the disease has evolved over the past 50 years. In the past, PHPT was primarily diagnosed due to severe bone loss, fragility fractures or renal calcifications, and lithiasis, rather than through the incidental discovery of hypercalcemia during routine biochemical screening (4). Therefore, the entity of asymptomatic PHPT (PHPT without overt signs and symptoms that typically is found by biochemical screening, with or without evidence of target organ involvement) and of normocalcemic PHPT have prevailed over the diagnosis of classic PHPT with severe multisystemic complications (3, 62, 63). However, regardless of the grade of involvement, bone remains 1 of the main target organs of the disease, causing site-specific BMD loss and predisposing to fragility fractures. Therefore, it is strongly recommended that BMD is measured at the hip, lumbar spine, and distal radius in all patients with PHPT (64). Delayed diagnosis may lead to aggravation of skeletal disease and development of more severe manifestations, such as *osteitis fibrosa cystica*, repeated fractures, and delayed fracture healing (51). Once PHPT is confirmed, treatment strategy must be developed. PTX is the definitive treatment for PHPT. Imaging studies are used to localize the abnormal gland(s), rather than confirming the diagnosis. Combined ultrasonography and parathyroid scintigraphy with Technetium Tc 99 m (sestamibi) increases localization accuracy preoperatively and aids toward revealing any coexistent thyroid disease. Four-dimensional computed tomography (CT), magnetic resonance imaging, single photon emission CT, and choline positron emission tomography/CT may be considered in challenging cases, where multiple gland involvement or ectopic parathyroid tissue are suspected or when the previous techniques failed to localize the disease (65). Indications for parathyroidectomy are summarized in Table 2 (61).

**Table 2.** Indications for operative treatment in PHPT (61)

Indications for parathyroidectomy in patients with primary hyperparathyroidism*^a^*
Serum calcium levels >0.25 mmol/L (>1 mg/dL) above upper limit of normal Bone involvement Osteoporosis (T-score ≤ −2,5 L1-L4, total hip/neck or radius 33%) Evidence of fracture by VFA or by any imaging technique Renal involvement Nephrolithiasis or nephrocalcinosis on imaging Hypercalciuria (24-hour urine calcium level >250 mg for women and >300 mg for men) Glomerular filtration rate <60 mL/min Patients aged 50 years or younger In the absence of all the above criteria, parathyroidectomy may still be the treatment of choice, when not contraindicated, if both the patient and doctor agree*^b^*

There are not definite data in favor of neurocognitive, life quality and cardiovascular indices improvement, thus surgery is not recommended for these indications.

Abbreviation: VFA, vertebral fracture assessment.

^a^The presence of 1 or more criteria justifies an indication for parathyroidectomy.

^b^Clinical or biochemical suspicion of parathyroid cancer, muscle weakness, impaired functional function, gastrointestinal symptoms, not desired or feasible surveillance according to protocols may be considered in the decision for parathyroidectomy.

#### Bone disease in sporadic PHPT

##### PHPT and bone turnover, BMD, bone quality, and fracture risk

PTH excess leads to acceleration of bone remodeling and swifts the balance toward bone resorption, leading to a high serum OPG and RANKL and a low OPG/RANKL ratio (24), usually associated with elevated biochemical bone turnover markers (66, 67). In fact, a significant positive correlation between serum PTH concentration and osteocalcin and alkaline phosphatase (ALP) has been proven (68). Increased remodeling mediated by PTH thins trabecular, endocortical, and intracortical surfaces, leading to enlarged medullary cavity, increased porosity, thinning of the cortex and production of cortical remnants that look like trabeculae (69, 70). It has been shown in numerous studies by bone densitometry (71-73) and histomorphometric analysis (29, 71, 74) that cortical bone is mostly affected, whereas trabecular bone seems to be preserved at least in studies from transiliac bone biopsies, in contrast to what is observed in postmenopausal state. However, Stein et al by performing individual trabecula segmentation, showed that there is less axial aligned trabecular network and reduced connectivity in PHPT with a decreased plate-like to rod-like trabeculae ratio (75). In addition, a recent study evaluating in vivo the bone material characteristics in PHPT patients using impact microindentation (76), demonstrated that patients with PHPT have lower Bone Material Strength index than normal individuals, and those with frequent fractures were the most affected.

Low BMD (77) mirrors the catabolic actions of PTH on bone, and therefore BMD should be monitored by dual energy X-ray absorptiometry (DXA), not only at lumbar spine, total hip and femoral neck, but also at distal 1/3 radius of PHPT patients, as a site of predominantly cortical bone, especially affected by PTH excess (78). BMD alone is not the only fracture risk predictor, because the underlying pathophysiology of PHPT significantly alters bone microarchitecture. Vertebral fracture assessment, Trabecular Bone Score (TBS) and high-resolution peripheral quantitative CT (HRpQCT) are useful tools for assessing microarchitecture, trabecular bone involvement, and fracture risk (61, 78). Data obtained through HRpQCT (70, 75, 79) and TBS (72, 80) have challenged the traditional belief and proved that trabecular bone is also affected in PHPT. The normal or high trabecular density reported in earlier studies may be attributed to inclusion of cortical remnants in the medullary compartment, as most imaging methods cannot distinguish these remnants from true, functional trabeculae (69). A recent meta-analysis highlights significant decrease in BMD at all sites in patients with PHPT compared with controls (81). TBS is a useful texture measurement tool, acquired during DXA scan, that offers valuable data regarding bone microarchitecture and fracture risk (80, 82). Eller-Vainicher et al described, among 92 patients with PHPT, a significant decrease of TBS in patients with mild PHPT who had a vertebral fracture (n = 3), compared to those without (n = 7), whereas no statistical differences in BMD were found (82). TBS is reduced in PHPT and correlated with spinal deformity index and vertebral fractures, regardless of BMD, age, body mass index, and gender (82). Tabacco et al recently reported that Bone Strain Index, a new bone quality index based on finite element analysis from lumbar vertebrae and femoral neck DXA images, was impaired in 50 PHPT patients when compared to 100 age- and sex-matched controls (83).

It has been confirmed that gender and geographic location can affect bone involvement in PHPT. Although biochemical severity of PHPT seems to be similar between pre- and postmenopausal women and men, the clinical presentation differentiates among the groups (84). Castellano et al (84). showed that premenopausal women and men are at significantly higher risk of developing nephrolithiasis, whereas osteoporosis is significantly more frequent among postmenopausal women, providing data regarding the influence of the menopausal status. No differences were found between men and premenopausal women regarding the lumbar spine or hip T-score. When US and Italian Caucasian women were compared regarding BMD, a significant difference between the 2 groups was observed, in favor of US female patients, both at femoral neck and total hip (85).

There are numerous epidemiological reports that demonstrate an increased fracture risk at both cortical and trabecular sites in patients with PHPT (86-89). As shown by Khosla et al (86), fracture risk is increased primarily at sites rich in cancellous bone, such as vertebrae, ribs, and distal forearm. Recent meta-analyses have provided more relevant data (81, 90). It was reported that patients with PHPT are at significantly increased risk for any fracture (relative risk, 1.71; 95% CI) (81) as well as forearm (odds ratio, 2.36; 95% CI) (90) and vertebral fracture (2.57; 95% CI) (81), compared to healthy controls. Older age, length of time since menopause, and decreased BMD at distal radius and lumbar spine are associated with further increase in vertebral fracture risk (81).

In summary, PHPT decreases BMD, affecting both cortical and trabecular bone mainly in postmenopausal women compared to men and premenopausal women. Apart from bone quantity, PHPT also reduces bone quality, leading to increased fracture risk.

##### Osteitis fibrosa cystica

**Figure 3.**
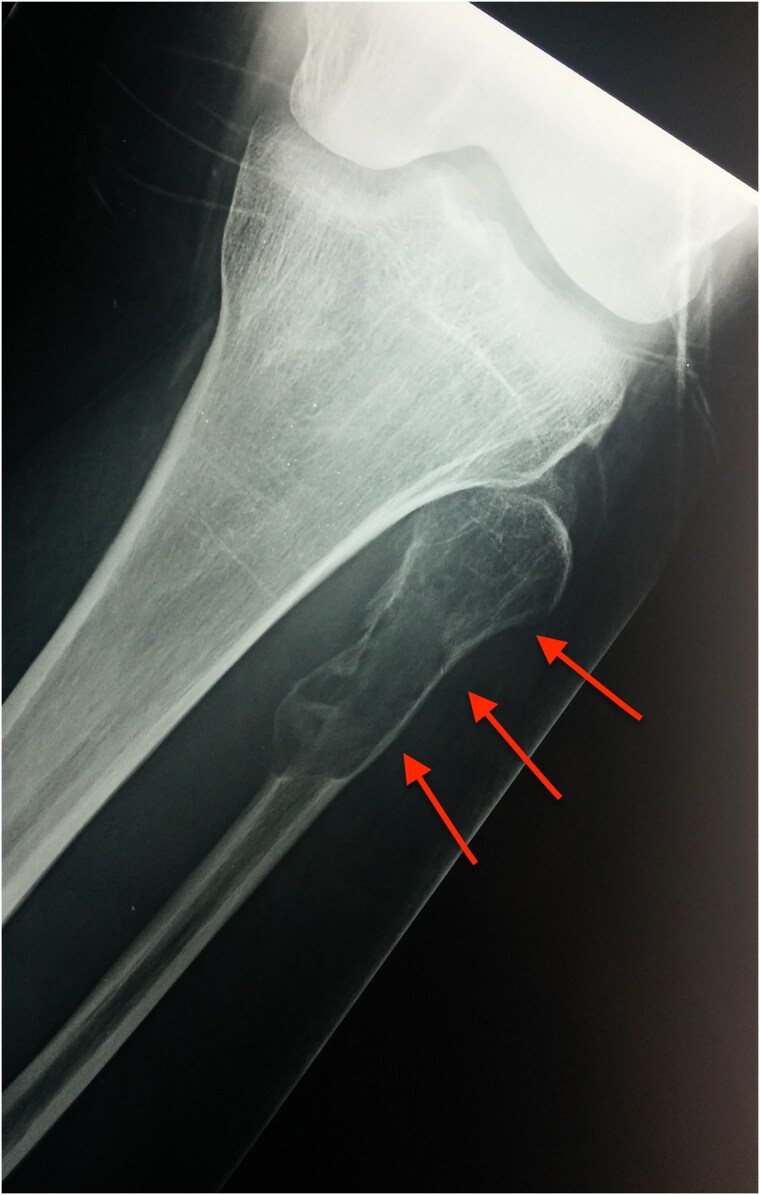
X-ray of the knee joint. Brown tumor of the fibula as a manifestation of primary hyperparathyroidism in a 52-year-old man.

As mentioned, the classic skeletal manifestations of *osteitis fibrosa cystica* are now rarely seen in developed countries and are restricted to severe or neglected cases of long-standing PHPT. Currently, the prevalence of *osteitis fibrosa cystica* in PHPT is less than 2% (91). However, it's far more common among cases of long-standing SHPT or tertiary hyperparathyroidism (THPT) because of chronic renal failure. Muscle weakness, bone pain, and pathological fractures may be the clinical presentation in *osteitis fibrosa cystica*. Although these patients usually present with nonspecific symptoms (eg, weakness, constipation, neuropsychiatric symptoms, nephrolithiasis), severe bone abnormalities are the milestone of the disease. The radiographic signs of “salt and pepper” or “ground glass” appearance resulting from demineralization of the skull, resorption of distal clavicle, subperiosteal resorption of the phalanges, cystic deformities, brown tumors, osteoclastomas of the long bones, and diffuse osteopenia are characteristics of this severe bone disease (92, 93). Brown tumors are nonneoplastic and histopathologically constitute osteoclasts, granulation tissue, vascular and fibrous tissue, brown hemosiderin deposition, and incompletely mineralized bone that result from excess osteoclast activity (91, 94). They are commonly found at jawbones, skull, pelvis, clavicle, ribs, femurs, and spine and may be single or multiple (Fig. 3). Their radiographic similarity to other benign (eg, aneurysmal or simple bone cyst, fibrous dysplasia) and malignant lesions may mislead the diagnosis. However, bone pain related to a brown tumor is milder compared to that of a malignant lesion and is accompanied by other characteristic signs of PHPT, such is “salt and pepper” appearance of the skull. BMD is particularly affected, but it is reversible after successful surgical treatment of PHPT (70). Severe symptomatic PHPT is characterized by markedly elevated serum calcium and PTH. Of note, PHPT is defined by increased bone remodeling, thus both biochemical markers of bone formation (eg, ALP, osteocalcin) and markers of bone resorption (eg, N-terminal telopeptide [NTX], C-telopeptide [CTX]) are typically notably elevated (93).

##### Bone involvement in normocalcemic PHPT

NPHPT is characterized by normal adjusted total calcium and normal ionized calcium levels along with elevated intact PTH on at least 2 measurements over a 3- to 6-month period, in the absence of other causes of SHPT. Among people submitted to DXA, NPHPT exhibits a prevalence of 0.18% (95).

One of the cohorts of NPHPT has revealed that bone phenotype is similar to controls, having normal bone turnover, no significant BMD and TBS impairment, and no increased risk of vertebral fracture (96, 97). On the contrary, previous studies showed that the majority of patients with NPHPT developed osteoporosis at spine and hip rather than the distal 1/3 of radius, with 57% prevalence of osteoporosis and 11% fragility fracture rate (98, 99).

Of note, by comparing NPHPT to HPHPT patients (100), it was demonstrated that patients with normocalcemia appear to become resistant to PTH's effects on the kidneys and bones. By studying patients with 40 NPHPT, 50 HPHPT subjects, and 80 age- and sex-matched controls with NPHPT, the authors found that the trabecular and cortical bones as assessed by Bone Strain Index were not significantly impaired in NPHPT (101). In agreement, in the World Health Organization MONICA study on a randomly selected Swedish cohort from the general population (102), there was no increase in fractures in normocalcemic PHPT patients over a 17-year period of follow-up.

To summarize, data regarding bone involvement are diverse; some studies imply that NPHPT doesn't affect skeletal properties; others suggest that BMD is diminished over time both at cortical and trabecular sites, lacking the preference for cortical sites found in hypercalcemic forms and to a lesser extent compared with hypercalcemic patients; fracture risk is mostly reported to be equal between patients with NHPTH and controls. Given the variability on the BMD data, it does raise the question if the entity of NPHPT is real or a biochemical diagnosis of values just falling outside of the “general population reference ranges” (103).

##### Bone involvement in asymptomatic PHPT

In an observational study, 80 asymptomatic patients, without nephrolithiasis or radiographic *osteitis fibrosa cystica*, having serum Ca^2+^ values <3.00 mmol/L, forearm *Z*-score >−2.5, and serum creatinine levels below 133 μmol/L, were followed without surgery (104). No progression of biochemical and bone density indices was reported up to 10 years of follow-up. Similar findings are revealed in numerous studies including PHPT patients without hypercalcemia/PTH-related symptoms or organ-specific symptoms (73, 105). However, decline in several aspects regarding quality of life was documented, using scores assessing social, physical, and emotional function, energy, and health perception (106). In a cohort of 101 asymptomatic patients (without *osteitis fibrosa cystica*, nephrolithiasis, classic neuromuscular symptoms, or hyperparathyroid crisis) followed up for 10 years, progression of the disease was observed in 25%, and it was associated with age, with patients younger than 50 years having a 3-fold risk of developing at least 1 new indication for PTX (73). However, these data don't prove a protection of asymptomatic patients over fractures. The introduction of TBS and HRpQCT in bone quality assessment has led to the documentation of more extensive bone lesions in asymptomatic disease than previously thought. In fact, the overall fracture risk is elevated in asymptomatic PHPT patients, too, reaching a 1.5-fold increase (89). By means of X-ray (107) and HRpQCT (70), it is shown that even in asymptomatic patients, excessive concentrations of PTH have an effect on bone geometry by inducing endosteal resorption and periosteal apposition, in a way that the inner diameter is enlarged to a greater extent than the outer diameter of the long bone, though retaining the cortical mechanical properties (70). Karlafti et al assessed the effect of PHPT on bone geometry using pQCT at the radius and tibia (108). They showed that both trabecular and cortical bone are affected, however, at the weight bearing bone, such as tibia, the deleterious effect is diminished when compared to radius, especially on cortical bone. A retrospective study of 56 asymptomatic PHPT patients, having no traditional and specific symptoms or signs of hypercalcemia or PTH excess, showed that PHPT adversely affects bone geometry (109). Cortical thickness of the femur neck was significantly decreased in those patients during 5 years of follow-up, suggesting that their hip might be prone to axial compressive stress (109).

In conclusion, asymptomatic patients, although usually stable, may experience biochemical progression and BMD, bone microarchitecture, and geometry deterioration, increasing the risk of fractures at both trabecular and cortical sites (78, 110).

#### Bone disease in syndromic PHPT

##### MEN-associated PHPT

PHPT resulting from parathyroid tumors may also occur as part of MEN syndromes, complex endocrine syndromes that are inherited in an autosomal dominant manner (111). MEN syndromes include 4 types (MEN1, MEN2A, MEN2B, MEN4) and represent approximately 5% to 10% of all PHPT cases. They are often associated with multiple parathyroid tumors (58, 59). In syndromic PHPT, the multiglandular asymmetric growth of parathyroid tissue may be missed at the initial surgery. Genetic testing is strongly recommended in PHPT patients aged <30 years or when a patient is highly suspected of having syndromic PHPT (multiglandular disease by imaging or history, cooccurrence of other endocrine tumors, atypical parathyroid adenoma and parathyroid carcinoma, familial history of hypercalcemia or syndromic diseases, such as MEN1, MEN2A, MEN4, and HPT-JT) (61).

MEN1 is the most common MEN syndrome, and it is caused by mutations of MEN1 gene that encodes a tumor suppressor protein (menin). It is a rare autosomal dominantly inherited syndrome, characterized by cooccurrence of parathyroid, anterior pituitary, and pancreatic islet cells tumors. Multiple hyperplasia and/or adenoma of the parathyroid glands causing PHPT is the most common endocrinopathy of MEN1. In fact, 95% of MEN1 patients present with PHPT as the first manifestation, with a mean age of 20 to 25 years (112). MEN1-related PHPT accounts for approximately 2% to 4% of all cases of PHPT (113), and it is estimated that the majority of MEN1 patients will develop hypercalcemia by the time they turn 50 years old (111).

Assessment of BMD using pQCT at the forearm has shown that, like in sporadic PHPT, MEN1-related PHPT is associated with reduced BMD when compared to healthy subjects (114). In a study of 68 patients with MEN1-related PHPT, only 18% had developed osteoporosis during the course of the disease (115). Another retrospective study including 29 women with MEN1 (116) reported that 86% of patients had reduced BMD. Authors concluded that bone loss is progressive from the late third to early fourth decade of life, with severe osteopenia affecting almost half of the patients by the age of 35 years. This earlier onset of bone involvement could interfere with the normal development of peak bone mass (117). The reduced bone mass was found both at femoral neck and lumbar spine and was associated with advanced age, uncontrolled PHPT, and increased prevalence of skeletal fractures (116). Although Wang et al demonstrated that MEN1 patients have lower BMD by DXA at all sites compared to sporadic PHPT patients, HRpQCT evaluation showed no significant differences in parameters of bone geometry, volumetric BMD (vBMD), or microarchitecture (117). Numerous studies have shown that MEN1 causes a more severe bone disease but similar renal disease compared to sporadic PHPT, despite the milder biochemical presentation (113, 117, 118). In a recent study, by comparing the skeletal involvement using DXA and TBS at the lumbar spine between 120 patients with MEN1-related PHPT and 360 patients with sporadic PHPT (119), the researchers found that although the BMD and TBS of the first group were lower than those of the second group, the proportion of skeletal involvement was not significantly different from the sporadic matched cases for gender and age. They also concluded that TBS score may be a sensitive index, in addition to BMD, in identifying cancellous bone microarchitecture impairment in patients with MEN1-related PHPT. The younger age at diagnosis (<50 years old) in combination with PTH levels in the normal range has been significantly associated with MEN1-related PHPT (113). These data disagree with the inverse correlation between PTH and BMD that is observed in sporadic PHPT (113). Thus, it is speculated that other factors apart from PTH increase may partially contribute to the severity of bone loss in MEN1-related PHPT; the coexistence of other endocrinopathies, including hyperprolactinemia, hypercortisolism, hypogonadism, and GH deficiency may also negatively affect bone mass (117). In addition, the greater level of bone involvement suggests a direct effect of the MEN1 gene on bone physiology, which has been proven by preclinical studies (120, 121). Osteoblasts in menin-deficient mice exhibit impaired mineralization and reduced responsiveness (120), whereas menin directly inhibits mesenchymal cell myogenic differentiation while accelerating osteoblast growth (121).

There are 3 clinical variants of MEN2: MEN2A, MEN2B, and medullary thyroid cancer (MTC)-only. MEN2A and MEN2B derive from gain-of-function mutations of the proto-oncogene RET, which encodes a tyrosine kinase receptor. MEN2A is the most common variant, characterized by MTC, parathyroid tumors (20%-30%), and pheochromocytomas (50%) (58, 122). HPT is usually asymptomatic and milder compared with MEN1 syndrome (122). Parathyroid tumors do not usually occur in MEN2B, which is characterized by MTC and pheochromocytoma, associated with a Marfanoid habitus, medullated corneal nerve fibers, mucosal neuromas, and intestinal autonomic ganglion dysfunction (111). However, it seems that skeletal manifestations in these patients are not restricted only to scoliosis, foot abnormalities, and marfanoid body habitus, but also extend to impaired skeletal metabolism and increased fracture risk. A recent study including 48 young patients (5-36 years of age) with MEN2B, compared them with healthy cohorts regarding fracture risk (123). The rate of fracture was significantly higher than in the general pediatric population (38% vs 19%), and the distribution of fractures was different, affecting the capital femoral epiphysis, the spine, and the long bones of the lower extremities, rather than the forearm.

MEN4 is caused by inactivating heterozygous mutations of a cyclin-dependent kinase inhibitor and includes a combination of multiple parathyroid tumors in 80% of cases, adenohypophyseal tumors in 40% of cases, whereas other tumors have also been reported, including pheochromocytomas and gastric tumors (112). There are insufficient data regarding bone disease in MEN4 syndrome, considering the small number of cases described (112).

In summary, MEN1 is characterized by a more severe bone disease because of the coexistence of PHPT with other endocrinopathies that negatively affect bone mass, whereas PHPT in the context of MEN2 syndrome is either milder (MEN2A) or scarcely occurs (MEN2B).

##### Hyperparathyroidism-Jaw tumor syndrome

HPT-JT syndrome is caused by inactivating mutations of the HRPT2/CDC73 tumor suppressor gene and consists of ossifying fibromas of the maxilla and mandible, adenomatous polyps of the uterus, and renal tumors. It is also characterized by increased risk of renal neoplasms and parathyroid carcinoma (112). The majority of patients develop PHPT, and the consequent bone involvement may present as osteoporosis and/or osteopenia, maxillary and mandibular ossifying fibromas, or even osteitis fibrosa cystica (112).

##### Familial hypocalciuric hypercalcemia

FHH is an autosomal dominant disorder resulting from inactivating mutations in the CaSR signaling cascade (124, 125). There are 3 distinct forms of FHH, namely FHH 1, 2, and 3, resulting from heterozygous loss-of-function mutations in the CaSR (located on chromosome 3q21.1, estimated prevalence 1/1000-5000), GNA11 (G-protein subunit alpha 11, located on chromosome 19p13.3—very rare), and AP2S1 (adaptor protein 2 sigma-1, located on chromosome 19q13.32-estimated prevalence 1/13 000) genes (124), respectively.

FHH1 is characterized by lifelong stable mild hypercalcemia, inappropriately normal/high PTH levels, normal or low-normal phosphate levels, and low urinary calcium excretion. Patients with FHH1 are generally asymptomatic, whereas patients with FHH3 tend to have higher calcium levels compared to FHH1.

Biochemistry between FHH and typical sporadic PHPT can be substantially overlapping. Although usually decreased (calcium-creatinine clearance ratio <0.01), urinary calcium excretion in FHH sometimes is borderline. In specific, approximately 40% of patients with either condition may have values in the 0.01 to 0.02 range (60, 61). Family history and genetic testing that is more available these days can be crucial to differentiate these 2 conditions to avoid unnecessary parathyroid surgery in FHH parents. FHH1 has been linked to >300 CaSR gene variations, with the majority of them consisting of missense replacements and affecting the first 350 amino acids of the extracellular domain of the receptor (126). In FHH2, only 4 loss-of-function GNA11 gene variations have been reported (T54M, L135Q, I200del, and F220S). FHH3 is caused by loss-of-function mutations in the APS1 gene that most commonly affect the R15 residue (Arg15Cys, Arg15His, and Arg15Leu) (125, 126).

There are some studies that evaluated bone disease in patients with FHH, especially type 1. Concerning areal BMD (aBMD), 2 studies (127, 128) evaluated patients with FHH1 as compared to patients with PHPT. They reported that those with FHH1 had normal aBMD at all sites, regardless of the severity of hypercalcemia (*Z*-scores not different from 0), and higher *Z*-score at the hip and the forearm as compared to patients with PHPT. aBMD at the spine was comparable. Bone turnover, in terms of ALP and urine NTX/creatinine was lower in FHH1 vs PHPT. Vertebral fractures were observed in 5% of cases with FHH. Similar findings in terms of aBMD were reported by Mouly et al (129). In this study, chondrocalcinosis was the most common finding as presented in 22% (11/51), whereas osteoporosis and fractures were found in 16% (6/38) and 14% (8/56), respectively. In a later study from the Danish group, researchers reported on 50 patients with FHH1 vs age- and gender-matched controls using aBMD by DXA, QCT at the spine and hip, and HRpQCT at the radius and tibia (130). aBMD and vBMD were comparable to controls in males, whereas females with FHH1 had higher vBMD and lower bone volume at the hip. Concerning microarchitecture, trabecular vBMD and trabecular thickness tended to be higher in patients FHH1, whereas estimated bone strength did not differ. Concerning FHH3, Vargas-Poussou et al (131) revealed that in 4 patients (of 22) tested with BMD, all had a lumbar spine BMD *Z*-score below −2.0. In addition, Hannan et al (132) reported that 5 of 10 patients aged 14 to 64 years with FHH3 tested had low BMD (T-score <−1.0 or *Z*-score <−2.0) at the lumbar spine or femoral neck.

Thus, available evidence indicates that bone strength is not compromised in patients with FHH, especially type 1. Definite diagnosis with appropriate genetic testing is mandatory and prevents misdiagnosis of PHPT and unnecessary surgical exploration. In cases with increased fracture risk, treatment with antiresorptive agents seems the best available choice, although evidence is lacking.

#### Effect of surgical treatment on PHPT-related bone disease

Successful surgical removal of the responsible parathyroid gland(s) remains the only definite treatment of PHPT. Among other criteria (Table 2), severe skeletal disease including pathological fractures, osteoporosis, and/or serum calcium levels >0.25 mmol/L (1 mg/dL) above the upper limit of normal constitute a strong recommendation for surgery (64). Primary goals of PTX are the restoration of normal serum calcium levels, the elimination of hypercalcemia-related symptoms, the reduction of fracture risk, and renal complications. When performed by experienced surgeons, PTX has a cure rate of more than 95% (57, 133) and low rates of complications (<1%) (77). Intraoperative PTH monitoring may be useful in assessing the successful parathyroid resection (4).

Patients who undergo PTX experience immediate normalization of PTH and calcium levels, decrease of bone remodeling markers over a period of few months (68, 73, 105, 110), as well as significant lasting improvement in subjective symptoms (134). In a retrospective cohort study (135), patients with PHPT were evaluated according to BMD and their fracture risk after PTX. Regarding both female and male patients’ total hip BMD, a significant but transient increase within 2 years of follow-up was recorded, followed by a decrease below the baseline after 5 years. Hence, regarding spine BMD measurement, a significant and sustained increase over 8 years of follow-up was noted. Another study showed dramatic and sustained improvement in bone remodeling and trabecular bone structure 3 years after PTX (67). The superior improvement in lumbar spine BMD compared to hip after PTX is opposite to the expected age- and estrogen-related bone loss over the years, especially at trabecular sites. A possible explanation is that trabecular bone is characterized by greater bone remodeling and thus offers a better ground for earlier and greater mineralization after successful PTX (94). Conflicting results have been found in studies looking at TBS alterations following PTX; some show improvement in TBS (82, 136), whereas others show no change (137, 138). These discrepancies may be attributed to variations in the population investigated, the severity of the condition, or sample size.

The improvement in BMD seems to be translated into substantially decreased risk of hip fracture and any fracture (87, 135). The results of a large retrospective cohort study (139), with a median follow-up of 6.5 years, showed that PTX is independently linked to a lower incidence of fracture (hazard ratio [HR], 0.68). Of 1569 patients with PHPT, 452 underwent PTX and the surgery was linked to an 8% reduction in the 10-year hip fracture rate and to a 3% reduction in the arm fracture rate, whereas no effect on spine, pelvis, and nonhip lower extremity fractures was observed. Fracture risk was not associated with calcium and/or PTH levels. In a large longitudinal cohort study including 210 206 older adults (>65 years old) (140), 70% were managed nonoperatively and 30% underwent PTX within 1 year of diagnosis. PTX was associated with lower risk for hip fracture (HR, 0.76) and any fracture (HR, 0.78) compared with nonsurgical treatment, whereas absolute fracture risk reduction was significant 1, 2, 5, and 10 years after PTX. Fracture risk also remained decreased after adjusting for a history of medical treatment for osteoporosis, cinacalcet, or steroid use. These results imply a clinically meaningful favorable effect of surgical treatment in such population.

##### Osteitis fibrosa cystica

Agarwal et al (141) retrospectively studied bone density, radiographic recovery, and biochemical markers of bone turnover after PTX in 51 patients with PHPT and *osteitis fibrosa cystica*. They observed early remineralization, improvement of bone mass at cancellous, but not so at cortical, bone sites. The sites of bone cysts, brown tumors, and fractures appeared abnormally dense 3 months after PTX. As for bone turnover markers, they documented an early decrease in urinary cross-lap levels, in the first week after PTX, and early increase of bone formation markers, ALP and osteocalcin, followed by a decrease 6 to 9 months after PTX. In a case report (91), excision of the parathyroid adenoma improved biochemical parameters, increased bone density and resulted in healing of the brown tumors. Spontaneous regression of an orbital brown tumor has also been reported, approximately 2 years after PTX (142), whereas in a recent retrospective study brown tumors completely regressed in a mean period of 12 months (range, 2-24 months) after PTX and normalization of PTH and calcium (143).

##### Normocalcemic primary hyperparathyroidism

In the absence of hypercalcemia, the therapeutic approach depends on investigations regarding end-organ involvement. The decision for surgery should be in line with the indications for PTX in asymptomatic patients (Table 2), hence patients without significant bone or renal disease should follow a structured observational policy. Successful removal of the responsible parathyroid gland seems to normalize postoperative PTH levels (98). Koumakis et al showed that successful PTX in NPHPT patients with osteoporosis leads to increase in BMD at the spine and hip at 1 year, comparable to that observed in hypercalcemic counterparts (144). However, due to limited evidence, current guidelines do not recommend surgery for NPHPT (61).

##### Asymptomatic hyperparathyroidism

Several studies have investigated and compared the effect of PTX vs observation in patients with mild asymptomatic PHPT. In a long-term observational study including mainly asymptomatic patients (110), Rubin et al reported significantly lower, normal-range biochemical parameters (serum calcium, PTH, urinary calcium) 15 years after PTX. In addition, significant postoperative increases in BMD were documented, remaining above baseline at all skeletal sites for the entire 15-year follow-up period. Of note, there was no significant difference regarding the postoperative increase in BMD between patients who were initially followed-up without surgery but ended up having PTX, and those who underwent PTX soon after diagnosis (110). In another study, 191 asymptomatic PHPT patients were recruited and randomized to medical observation or surgery (105). Compared with the observation group, operated patients demonstrated a substantial increase in BMD both at lumbar spine and femoral neck 1 and 2 years after surgery. Similar results are documented in multiple randomized controlled trials (RCTs) (106, 145). In a systematic review and meta-analysis (133), Ye et al demonstrated that there is high-quality evidence regarding the achievement of biochemical cure in the majority of asymptomatic patients undergoing PTX (96.1%, 95% CI). Moreover, they reported that there was a significant increase in lumbar spine and total hip BMD postsurgically (mean difference 4.82; 95% CI and mean difference 4.41; 95% CI, respectively). However, data regarding fracture risk, both vertebral and nonvertebral, as well as regarding quality of life post-PTX were uncertain or of low quality. In addition, a recently published systematic review of 8 RCTs including 447 adults with PHPT (the majority of cases were asymptomatic) (146), reported that probably PTX leads to a significant increase in the cure rate compared with observation; however, it potentially has little or no effect on serious adverse events (including hospitalization for hypercalcemia), and uncertain effect on BMD.

In a prospective cohort of 177 women with primary hyperparathyroidism, PTX was related with a significant improvement in BMD in all measured sites (total hip, hip neck, lumbar spine, forearm) 1 year after surgery (147). Of note, the authors documented that PTX was associated with BMD gain regardless of baseline osteopenia or osteoporosis. In the same study, preoperative levels of serum CTX and procollagen type 1 N-terminal propeptide (P1NP) found to be higher in osteopenic women who exhibited the higher BMD gain postoperatively.

The Scandinavian Investigation on Primary Hyperparathyroidism study was a prospective RCT assessing the effect of PTX compared to observation on BMD and biochemical markers in patients with mild asymptomatic PHPT after 5 years of follow-up (148). The researchers concluded that there was an increase in BMD in the PTX group at all sites (the increase at lumbar spine BMD was significant, from a mean of 1.051 at baseline to 1.086 at 5 years), except for the 1/3 distal radius, where there was a progressive decrease of BMD regardless of treatment. P1NP and CTX were significantly diminished in the PTX group, but the variations between the 2 groups were only significant for CTX. An interesting observation of the study was the significant decrease in BMD at all sites except for the lumbar spine in the observation group, in contrast to the data from previous studies, which suggested that BMD remained unchanged for a long period of follow-up (73, 110). The authors speculated that this discrepancy might be due to the high prevalence of progressive spinal osteoarthritis among the population studied, as proven by radiographic data for spinal fractures. The 10-year extension of the study (149) confirmed the significantly positive effect of PTX compared to observation on lumbar spine BMD, over the entire follow-up; BMD was significantly diminished at all sites in the observation group. Of note, the results were null regarding fractures (149). Mortality, fracture, and cardiovascular and renal disease rates did not increase in the observation group during the 10-year follow-up period (150). Thus, it seems that observation might be a safe alternative for this group of patients.

A recent meta-analysis of 5 RCTs and 30 cohort studies compared fracture risk and BMD changes in PHPT patients receiving PTX (73 778 patients) vs observation (164 410 patients) (151). Significant reductions in fracture risk at any site and notably hip were reported in PTX compared to the observational group. These reductions were not accompanied by corresponding increases in BMD, implying the existence of other mechanisms through which PTX can impact fracture risk reduction independently of BMD. However, credibility issues were raised by authors that both RCTs and non-RCTs included studies affecting the meta-analysis study quality. Another meta-analysis by Cironi et al compared the effect of PTX vs medical management (calcium-lowering or modifying agents) in patients with mild asymptomatic PHPT (152). The authors reported that PTX leads to a prompt and significant reduction of calcium and PTH, reaching lower levels than the preoperative ones. On the contrary, the medically managed group demonstrated markedly elevated levels of posttreatment calcium, reaching the baseline values, a result that is possibly attributed to poor adherence to treatment. Concerning BMD, medically treated patients exhibited lower BMD at lumbar spine, femur, and hip, than surgically treated patients, who didn't experience significant alterations. However, the short-term follow-up of approximately 3.5 years, showed no significant difference regarding the incidence of fractures between the 2 groups.

Regarding TBS, surgically treated patients with asymptomatic PHPT exhibited significantly increased TBS 24 months after PTX, whereas conservatively followed-up patients tended to present decreased TBS (82). TBS decrease was correlated with the risk of vertebral fractures, independently of BMD (82).

To summarize, data from studies comparing PTX vs observation in asymptomatic PHPT patients show that PTX is significantly associated with biochemical cure and increase in BMD at lumbar spine and femoral neck; data regarding fracture risk remain inconclusive.

##### MEN1-associated PHPT

Surgical intervention is the treatment of choice for patients with hypercalcemia due to genetic PHPT. However, it can be challenging because of asynchronous multiglandular involvement, related to higher rates of recurrent hypercalcemia (approximately 20%-60% of MEN1 patients, compared to only 4% sporadic PHPT patients (153, 154)). Regarding postoperative bone assessment, Burgess et al (116) reported that BMD at the femoral neck and lumbar spine in MEN1 patients with PHPT showed an average of 5% and 3% improvement after surgery, respectively. In addition, Marini et al (59) confirmed the improvement of bone mass after parathyroidectomy at all the evaluated bone sites, but without reaching a statistical significance.

#### Hungry bone syndrome

Hungry bone syndrome (HBS) is a rare and serious event after successful PTX in PHPT patients with severe bone involvement. The term refers to a rapid, profound, and prolonged postoperative hypocalcemia that is accompanied by hypophosphatemia and hypomagnesemia (155). Hypocalcemia is thought to result from an increased influx of calcium into the bone because of the abrupt decrease in bone resorption and thus release of calcium from bone following PTX and accelerated mineralization, especially in patients with high preoperative bone turnover and thus are high remodeling transient. This condition can result in severe hypocalcemia, requiring IV calcium administration. Although replacement of calcium, vitamin D, and magnesium should be immediately considered, a universal therapeutic approach has yet to be established. Vitamin D deficiency was thought to increase the risk for HBS, and early postoperatively supplementation with high doses of cholecalciferol has been recommended (93). However, recent data suggest that preoperative supplementation in vitamin D-deficient PHPT patients undergoing PTX has no preventive role regarding hungry bone incidence (156). Several other prognostic indicators for developing HBS have been proposed across various studies, including biochemical (increased preoperative serum ALP, PTH, calcium, and urea levels), clinical (older age, existence of brown tumors and osteitis fibrosa cystica), parathyroid tumor features (increased weight, atypical, carcinomas) as well as prolonged parathyroidectomy time (157). A few studies and case series have suggested a protective effect of preoperative bisphosphonates against HBS in PHPT (158, 159), but since bisphosphonates could empirically worsen postoperative hypocalcemia and there are no randomized trials assessing their efficacy, preventive use of antiresorptive drugs to avoid hungry bone syndrome is still not routinely advised.

#### Effect of pharmacological treatment on PHPT-related bone disease

Because there is no curative pharmaceutical treatment, nonsurgical treatment is a choice in symptomatic patients for whom surgery is contraindicated or when localization of the enlarged parathyroid gland(s) is challenging or in patients who don't wish to undergo surgery. In addition, it can also be considered in asymptomatic PHPT. The effect of medical treatment on bone metabolism is summarized in Table 3 (55, 56, 160-175).

**Table 3.** Pharmacological approaches and their effect on bone metabolism in primary hyperparathyroidism

Agent	Serum calcium	PTH	BTMs	BMD	References
Bisphosphonates (alendronate)	=	=	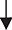	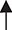	(160, 161)
Denosumab	=, 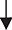	=, 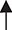	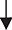	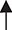	(162)
Cinacalcet	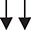	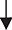	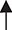 , 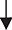	=	(163-165)
Estrogen	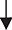	=	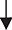	=, 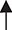	(56, 166-168)
Raloxifene	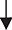 , =	=	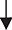	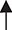	(55, 169, 170)
Combination cinacalcet + alendronate	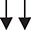	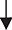	—	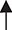	(171)
Combination cinacalcet + denosumab	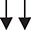	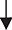	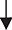	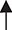	(172)
Calcium	=	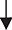	—	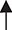 *^a^*	(173)
Vitamin D analogs	=	=, 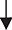	—	—	(174, 175)

Abbreviations: 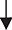, decrease; 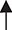, increase; 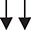, marked decrease; =, nonsignificant change; -, no data; BMD, bone mass density; BTMs, bone turnover markers.

^a^In asymptomatic primary hyperparathyroidism patients with a low-calcium diet.

Independently of the agent chosen, general measures should be taken; adequate hydration is important to prevent exacerbating hypercalcemia; thiazide diuretics should be avoided because they increase renal calcium reabsorption; lithium should be avoided because it may raise PTH by raising the set point at which extracellular calcium suppresses PTH secretion (176). Normal calcium intake (as indicated in the general population) and vitamin D supplementation in deficient patients with a goal of >20 or even >30 ng/mL are usually also recommended in patients with PHPT (161). Oral bisphosphonates, especially alendronate, have been investigated regarding their effectiveness in improving the bone aspects in PHPT. Their use led to reduction of bone markers and increase in BMD at the femur, total hip, and spine, although serum calcium and PTH levels remained unchanged (177, 178). There are no data regarding fracture risk reduction. In addition, no evidence concerning the effect of IV bisphosphonates in the treatment of PHPT is available. Denosumab is another antiresorptive agent that has favorable outcomes regarding BMD at the femoral neck, total hip, and lumbar spine, as well as TBS (136, 162). Cinacalcet is useful in reducing serum calcium and PTH serum levels significantly; however, it has no effect on BMD (163, 179). Hormone replacement therapy and selective estrogen receptor modulators (SERMs) seem to increase BMD and reduce bone turnover markers, although they have minimal effect on calcium and PTH levels (161). Relevant data regarding the effect of the nonsurgical pharmacological treatments on the PHPT-induced bone damage are provided next.

##### Bisphosphonates

Bisphosphonates are pyrophosphate analogs that act as very potent inhibitors of bone resorption. They directly lead to osteoclast inactivation by inhibiting farnesyl diphosphate synthase in the cholesterol pathway and thus suppressing isoprenylation (180). The rationale for their use resides in their antiresorptive potency and in the high rates of bone turnover in PHPT.

A systematic review and meta-analysis highlighted that alendronate remains the most widely investigated bisphosphonate regarding PHPT management (160). Alendronate use was documented in 12 studies, where treatment lasted anywhere from 5 days to 2 years, with 9 reports of at least 48 weeks. The researchers concluded that most studies agreed on an initial significant decrease in serum calcium levels for about 6 months after alendronate initiation, and then over time an increase back to baseline levels. PTH levels followed a similar pattern. Moreover, biochemical bone markers, which mirror bone metabolism and turnover, decreased in all studies. More pronounced gains in BMD were reported in trabecular-rich compared to cortical-rich sites, as all 9 long-term studies agreed in BMD increase at lumbar spine and hip, whereas some of the studies showed no change or even decrease of BMD at distal radius. The positive effect of alendronate in bone metabolism has been proven in every PHPT clinical variant, including symptomatic (181), mild (178), normocalcemic (182), and asymptomatic PHPT (177), offering a useful alternative to surgery regarding skeletal protection, in PHPT patients with osteoporosis not proceeding to PTX for any reason.

Risedronate administration demonstrated a significant acute decrease of serum calcium (183), but no effect on BMD and bone mineral content, as assessed by DXA and pQCT, respectively (184).

Interestingly, increases in BMD do not always reflect a decrease in fracture risk (185). Yeh et al studied retrospectively 6272 patients with PHPT regarding the effectiveness of surgical or bisphosphonate treatment compared to observation (alendronate was used in 92% of treated patients) (135). They concluded that in contrast to what was observed with PTX, increases in BMD with bisphosphonate treatment did not translate into a benefit regarding fracture risk. In fact, it seems that fracture risk is even more increased in those treated with bisphosphonates compared with untreated patients. However, these results may be attributed to the older age and lower baseline BMD of patients treated with bisphosphonates compared with the untreated patients (median age 71 vs 65 years and median baseline BMD 0.56 vs 0.66, respectively); moreover, the prevalent fracture at baseline was not reported. In a recent systematic review and meta-analysis, no significant effect of bisphosphonate therapy on fracture risk was observed (186).

##### Denosumab

Denosumab is a human monoclonal antibody targeting RANKL, which is a pivotal mediator of bone resorption (187). The indication of use regardless of the patient's renal filtration rate (187) is of clinical significance, primarily when treating elderly or patients with PHPT and severe renal involvement. Indeed, it was proven that denosumab is a safe and efficient treatment option for postmenopausal women with mild-to-moderate chronic kidney disease (CKD) and PHPT (188). After 24 months of treatment, BMD was considerably elevated in patients with CKD and osteoporosis related to PHPT; in addition, a significant drop in calcium levels was reported in the PHPT with CKD group compared to the PHPT without CKD group. The risk of hypocalcemia and severe hypocalcemia is higher among CKD patients, with late stages of CKD (3b-5) and lower baseline calcium levels representing the main predisposing factors (189-191).

Eller-Vainicher et al first reported the beneficial effect of denosumab on skeletal metabolism in 25 women with PHPT-related osteoporosis (162). After 24 months of treatment, PHPT patients experienced significantly increased BMD at femur, total hip, and spine compared to baseline values as well as compared to primary osteoporosis patients, whereas no change was observed in laboratory values including calcium, PTH, and ALP. In addition, a recent randomized double-blind trial by Leere et al (172) proved that denosumab treatment significantly increases both spine and total hip aBMD and vBMD, reduces bone turnover and the biochemical turnover markers, and, when combined with cinacalcet, improves biochemical indices in patients with PHPT.

In a meta-analysis, denosumab was compared to alendronate regarding its effect on bone metabolism in PHPT patients (186). In 2 studies included in this meta-analysis assessing serum calcium after denosumab treatment, a prompt decline in serum calcium and a subsequent increase up to the baseline levels was reported. Although alendronate led to a more long-term (up to 12 months) decrease in serum calcium, the effect disappeared as well, and calcium levels returned to baseline after 24 months of treatment. Denosumab therapy demonstrated no effect on PTH levels; the initial effect of alendronate in increasing serum PTH subsided, as PTH returned to baseline at 24 months of alendronate use. Twelve-month denosumab treatment significantly increased aBMD at lumbar spine and femoral neck compared to baseline. The effectiveness of denosumab in restoring bone metabolism in PHPT patients was also shown in a retrospective, longitudinal study (136). In this study, patients treated with denosumab once every 6 months were compared to those who successfully underwent PTX. Denosumab not only appeared equivalent to PTX in improving BMD but also was proven to be superior in improving lumbar spine TBS. Although these studies didn't show any efficacy regarding calcium levels, others have demonstrated that denosumab is a successful bridge to PTX, as it mediates rapid control of hypercalcemia in patients with severe symptomatic hypercalcemia due to PHPT, who are not candidates for immediate surgical management (192, 193). However, it should be administered with caution because there is a risk of developing short-term or protracted hypocalcemia, with renal insufficiency, prolonged hyperparathyroidism, parathyroid cancer, and vitamin D deficiency being the main risk factors (194). Moreover, a case of marked hypophosphatemia related to denosumab treatment in a patient with NPHPT should raise awareness toward the importance of adequate calcium and vitamin D supplementation in these patients (195, 196).

##### Calcimimetics

Cinacalcet is the first available calcimimetic drug, which increases the sensitivity of the CaSR in parathyroid cells to the circulating calcium, thus leading to a decreased synthesis and secretion of PTH, and in turn of calcium (197). It was first approved for patients with SHPT and consequently was available as a treatment alternative to PTX for patients with PHPT (198). Indications for its use in hyperparathyroidism are summarized in Table 4 (197, 199). Large clinical studies over the years have proven its prolonged efficacy in normalizing calcium and reducing PTH levels in PHPT patients (163, 164, 200-204). A systematic review and meta-analysis (205) have recently highlighted the efficacy of this well-tolerated drug in reducing and normalizing calcium levels in nearly 90% of patients, which is accompanied by a significant increase in phosphate levels. Although PTH levels are diminished, only 10% of patients reach normalization. The results of PRIMARA study (206), a multicenter prospective observational study including 303 PHPT patients, confirmed the efficacy of cinacalcet in diminishing calcium levels in PHPT. Sixty percent of patients treated with a median daily dose of 30 mg experienced ≥1 mg/dL (>0.25 mmol/L) reductions of calcium levels after 12 months of cinacalcet treatment. The main adverse events included gastrointestinal symptoms and no significant safety concerns were reported. Cinacalcet seems to be equally effective in normalizing serum calcium over a wide range of PHPT disease severity, ranging from asymptomatic disease to end-organ complications (165). Low-dose cinacalcet (30 mg per day) is a well-tolerated and effective treatment of PHPT hypercalcemia and may be of choice as a bridge to surgery in PHPT patients with severe hypercalcemia and bone disease who are at risk of postsurgical HBS (197, 203). Additional favorable effects of the drug regarding serum phosphorus and urinary calcium excretion have also been reported (202, 207). The effect on bone turnover as measured by biochemical bone markers appears to be diverse (160). In a recent systematic review and meta-analysis of RCTs and cohort studies, it was confirmed that cinacalcet normalizes serum calcium and significantly reduces PTH levels (179). However, cinacalcet demonstrated no beneficial effect on BMD at all sites in two nonrandomized studies (163, 208), whereas RCTs reporting on the effect on BMD as a primary or secondary outcome are still missing. Therefore, combined treatment with an antiresorptive drug may be considered in PHPT patients with overt bone disease (209).

**Table 4.** Indications of cinacalcet administration in patients with hyperparathyroidism (199, 201)

Therapeutic indications of cinacalcet	
Primary hyperparathyroidism	Contraindication or unwilling patient for PTX despite meeting the criteria Persistent or recurrent PHPT after PTX Hypercalcemia in parathyroid carcinoma Multiple hyperplasia or MEN1-associated PHPT
Secondary hyperparathyroidism	End-stage renal disease or maintenance on dialysis therapy Persisting SHPT after renal transplantation

Abbreviations: PHPT, primary hyperparathyroidism; PTX, parathyroidectomy; SHPT, secondary hyperparathyroidism.

It has been reported in multiple studies that cinacalcet reduces parathyroid gland volume in patients with SHPT (210-212). In fact, cinacalcet has a proven apoptotic effect in parathyroid cells of SHPT patients, as assessed by histological and cytological analyses (213). The first study to evaluate this effect in PHPT patients was recently conducted by Minezaki et al (198). The researchers observed that, after a 6-month cinacalcet treatment, the size of parathyroid adenomas was reduced by 29% in patients with PHPT. Moreover, a few cases of spontaneous PHPT remission during cinacalcet treatment have been reported (208, 214).

Patients with MEN1-related PHPT are more prone to recurrence after PTX and to surgical complications from multiple neck explorations. Cinacalcet can be effectively used in patients with MEN1-associated PHPT with persistent or recurrent PHPT after PTX or in cases where surgery is contraindicated (215). In a randomized, double-blind, crossover study including 15 MEN1 patients with hyperparathyroidism (216), cinacalcet led to normalization of serum calcium, significant increase in serum phosphate, and reduction of PTH levels in all patients after 3 months of therapy. Reduction of PTH levels to the normal range was reported in about 50% of the patients. Although cinacalcet normalizes calcium levels and is more potent in reducing PTH levels in MEN1 compared to sporadic PHPT, no modification of parathyroid tissue size or BMD has been observed (199, 216, 217).

##### Estrogen replacement therapy

As postmenopausal women are most affected by PHPT, estrogen replacement has early been identified as a potential pharmacological treatment. Short-term administration of ethinylestradiol in postmenopausal women with mild PHPT resulted in significant decrease of serum calcium levels and urine calcium and hydroxyproline levels, compared to baseline values, due to inhibition of bone resorption (56, 166). Similar results were observed with norethindrone therapy; however, neither of the 2 estrogen treatments was related to significant alterations of PTH plasma concentrations (56). A 2-year RCT including 42 menopausal PHPT women evaluated the effect of conjugated equine estrogens and medroxyprogesterone acetate on bone metabolism (167). Although no significant variations in plasma ionized calcium levels were observed, bone metabolism biochemical markers and urine calcium levels were decreased. BMD significantly increased at all skeletal sites. The adverse events of mastalgia and vaginal bleeding were mild and self-limited. The 4-year extension of the study included 23 patients from the initial cohort (168). The researchers reported slightly lower serum calcium levels, decreased bone turnover markers and significantly increased BMD compared to placebo at all sites (7.4% at femoral neck, 7.5% at lumbar spine, 7% at forearm, and 4.6% at total body). The initial reduction in urine calcium levels wasn't maintained for 4 years.

##### Raloxifene

Raloxifene is a SERM acting as an estrogen receptor agonist on skeleton and lipid metabolism and as an estrogen antagonist on breast and uterus. It is the only SERM studied regarding therapeutic potential in PHPT. Three postmenopausal osteopenic women with mild, asymptomatic PHPT, who declined hormone replacement therapy or PTX, were given raloxifene 60 mg or 120 mg daily for a year (169). After 1 year of treatment, BMD showed a 3.4% increase at lumbar spine and a 2.5% increase at femoral neck, whereas serum total calcium levels, serum phosphate levels, urine deoxyproline, and calcium excretion significantly decreased. However, ionized calcium and intact PTH levels returned close to baseline values at the end of the study. A larger randomized study of 18 patients with mild asymptomatic PHPT (55), also showed a decrease in serum calcium and serum NTX in patients treated with raloxifene compared to controls, but PTH, serum phosphate, calcitriol, ALP, and urinary calcium excretion remained unchanged. Calcium and bone marker changes were reversed after discontinuation of raloxifene. When compared to alendronate, raloxifene was inferior in decreasing serum calcium levels in postmenopausal women with PHPT and osteoporosis (170). On the other hand, raloxifene and alendronate had an equally significant effect in increasing BMD at the lumbar spine after 12 months of therapy (170).

##### Calcium and vitamin D

Dietary calcium intake is pivotal in regulating calcium metabolism, with increased intake resulting in suppression of PTH secretion and inhibition of parathyroid cell proliferation. In a large prospective 22-year-long study, including more than 58 000 women from the Nurses’ Health Study 1, researchers examined the relationship between dietary calcium intake and the development of PHPT (218). They concluded that increased calcium intake was independently related to reduced risk of PHPT. It has been reported that when calcium supplementation is given cautiously to asymptomatic PHPT patients with a low-calcium diet, there is a significant decrease in PTH levels and significant increase in femoral neck DXA score, without significant increase in serum or urinary calcium (173). However, with the possibility of unacceptable serum (173) and/or urinary (219) calcium elevation looming, it is more rational to follow the guidelines of the Institute of Medicine for adequate calcium intake in the general population and monitor PHPT patients closely, at least during the first year of calcium supplementation (94, 173).

Vitamin D insufficiency appears with an increased prevalence among PHPT patients compared with general population (220). However, it seems that lower total 25OH-vitamin D levels described in patients with PHPT don't agree with their free 25OH-vitamin D levels because the latter may be similar (221) or even higher than healthy controls (222). Numerous articles have been published regarding both the role of vitamin D status of PHPT patients in the severity of bone disease and the effect of vitamin D supplementation of deficient PHPT patients in the aspects of the disease. In recent studies, vitamin D deficiency significantly increased the risk of postoperative hypocalcemia/hypoparathyroidism, of higher requirements in calcium supplementation, and of longer hospitalization (223, 224). In addition, it has been associated with increased parathyroid gland weight, higher PTH, ALP, and calcium levels (225), as well as more end-organ involvement, including reduced BMD (226) and deteriorated hip geometry in postmenopausal PHPT women (227). On the contrary, Walker et al reported that vitamin D levels weren't associated with nephrolithiasis, osteoporosis, fractures, serum or urinary calcium, biochemical bone turnover markers, or BMD (228). Moreover, the same research group documented no significant differences regarding bone microarchitecture and strength as assessed by HRpQCT among PHPT patients with vitamin D deficiency (25OH-vitamin D <20 ng/mL), insufficiency (20-29 ng/mL), and repletion (≥ 30 ng/mL) (229).

Clinical studies investigating the effect of vitamin D supplementation in PHPT patients also have yielded controversial results, regarding significant improvement of biochemical aspects of the disease. A meta-analysis of 10 observational studies (174) including patients with mild PHPT demonstrated no significant changes in PTH, serum calcium and phosphate, and urinary calcium excretion in patients receiving vitamin D supplements. Hence, Song et al, in their meta-analysis, concluded that supplementation of vitamin D in deficient patients with PHPT leads to a significant reduction of PTH and ALP levels, without causing hypercalcemia or hypercalciuria (175). Another meta-analysis confirmed that supplementation with vitamin D in deficient patients with mild PHPT is safe and isn't associated with worsening of hypercalcemia or hypercalciuria (230). However, lacking conclusive data about the effect of vitamin D supplementation on the outcome of the disease, it is prudent to follow the recommendations for well-monitored vitamin D supplementation in PHPT who are deficient, with a goal of 25OH-vitamin D levels between 20 to 30 ng/mL (226).

##### Combined treatment

Many research groups have studied the effect of combination treatment in PHPT, both the combination of a pharmacological agent with surgery and the coadministration of 2 different agents. As for the first, recent studies examining the effect of adding bisphosphonates after PTX have controversial results. A randomized, placebo-controlled study (231) compared the effects of surgery alone with the effects of surgery combined with zoledronic acid on bone metabolism in PHPT patients with osteoporosis. The evaluation 2 years after PTX showed significantly higher BMD both at the femoral neck and lumbar spine, and lower bone turnover markers in the zoledronic acid group compared to placebo group. A retrospective cohort study including 1737 patients (232) showed that although treatment with bisphosphonates after PTX is related to greater increase in BMD than PTX alone, it seems that adding bisphosphonates diminishes the beneficial effect of PTX alone on fracture risk. On the other hand, in a retrospective analysis (233), Choe et al demonstrated no additional benefit in BMD with combination treatment over PTX alone in patients with PHPT and osteoporosis. As previously mentioned, a few studies suggest that preoperative bisphosphonates seem to have a protective effect of development of HBS (158, 234, 235).

Another combination therapy that have been studied is coadministration of an antiresorptive agent and cinacalcet. Faggiano et al retrospectively evaluated the efficacy of combination of alendronate and cinacalcet in bone disease in 10 patients >50 years old with overt PHPT and reduced BMD (171). Patients experienced a rapid decrease of hypercalcemia and hypercalciuria, with normalization of serum calcium levels, decrease of serum PTH, and stabilization of the results after 24 months of follow-up. When authors compared the combination group with a similar group receiving only cinacalcet, they observed that the beneficial effect on biochemical abnormalities of the disorder was similar. However, the combination group experienced a significant increase in BMD at lumbar spine (9.6%), whereas cinacalcet-alone group had no changes in BMD. Leere et al recently compared the efficacy of denosumab alone with combination with cinacalcet in PHPT patients in a randomized, double-blind, placebo-controlled, phase 3 trial (172). They concluded that both denosumab alone and combination treatment are associated with similar significant increases in lumbar spine aBMD, total hip aBMD, and spine vBMD. Compared to the combination or placebo groups, the denosumab group experienced a substantially greater mean rise in ultradistal forearm vBMD but there were no significant differences in the ultradistal forearm between groups regarding cortical width or cortical or trabecular vBMD. They proved that denosumab is effective in reducing bone turnover, and if combined with cinacalcet, can safely improve the biochemical profile of patients with PHPT.

In summary, it seems that although bisphosphonate therapy following PTX may be associated with BMD improvements, this effect is not translated into fracture risk decrease, possibly due to the inhibition of bone turnover and a reduction in bone formation that follows PTX. On the contrary, PTX alone is superior when it comes to fracture risk. Although data are limited, the combination therapy of bisphosphonates with cinacalcet could achieve both stabilization of BMD by bisphosphonate treatment and calcium-lowering effects of cinacalcet.

### Parathyroid Carcinoma

Less than 1% of all sporadic PHPT cases are caused by the rare parathyroid carcinoma (236). It may occur sporadically or as part of a genetic syndrome associated with parathyroid tumor (HPT-JT syndrome, MEN1, MEN2A, isolated familial hyperparathyroidism); up to 15% of cases of hereditary HPT-JT syndrome suffer from parathyroid cancer (236, 237). Patients tend to be younger than their counterparts with benign adenomas (238). The etiology is largely unknown, although a variety of mutated genes have been identified, such as HPRT2/CDC73, mTOR, and KMT2D (236). Although specific risk factors for the sporadic form are yet to be identified, some of the reported cases have a history of neck radiation, secondary/tertiary hyperparathyroidism, or adenoma/hyperplasia of parathyroid gland(s) (239-243). Parathyroid carcinoma is characterized by indolent course and low potential of distant metastasis. The most common clinical presentation includes functional tumors with overt hyperparathyroidism- and hypercalcemia-associated symptoms and features (general symptoms, bone and renal involvement, cardiac arrhythmias, neurocognitive impairment). However, normal serum calcium levels in the context of normocalcemic PHPT, don't exclude the possibility of parathyroid malignancy (244). As previously mentioned, most cases of PHPT are recognized early because of the inclusion of calcium and PTH levels measurement in the pro-symptomatic laboratory screening in developed regions. However, multiple cases with pathological fracture as the first presentation of the disease have been described (245-248), reflecting the severity of HPT and related bone disease. The nonfunctioning clinical variant is extremely rare and challenging to diagnose because it is characterized by normal PTH and calcium levels and, therefore, is usually diagnosed late in the course of the disease because of local or distant spread or to diagnostic postoperative histological examination (249). Although definite diagnosis is most often made after surgical resection of the tumor, markedly elevated PTH and calcium levels (PTH > 1000 pg/mL and hypercalcemia >14 mg/dL (246)), severe end-organ involvement and a large parathyroid lesion with malignant-like ultrasonographic features with or without hoarseness and dysphagia should raise suspicion of parathyroid carcinoma (250, 251). Fine-needle aspiration is not recommended because the results usually don't differentiate malignant from benign tumors and moreover increase the risk of tumor seeding (252). “En bloc” resection of the parathyroid tumor represents the gold standard treatment, yet not always curable, as it tends to recur locally, requiring multiple surgical procedures (251). The NEKAR study is a retrospective international cohort study that evaluated the recurrence-free survival of 83 patients with parathyroid carcinoma (253). Skeletal complications were recognized in approximately 23% of patients, with renal symptoms being the most common, affecting approximately 40% of them. Recurrence of the tumor was reported in 32 cases, despite the overall good prognosis of the malignancy. The efficacy of classic adjuvant therapies, such as radiotherapy and chemotherapy, in cases of persistent, recurrent, or metastatic disease is disappointing. In case of nonresectable tumor, medical management of hypercalcemia is crucial because it represents the major cause of mortality (252).

#### Bone disease in parathyroid carcinoma

In general, bone involvement in parathyroid carcinoma patients doesn't differ significantly from other forms of PHPT. However, the majority of these tumors are hormonally functional and lead to symptoms and complications of profound hypercalcemia. Hypercalcemic crisis is more common due to functioning parathyroid carcinoma than benign parathyroid adenomas and is a medical emergency requiring immediate intervention (237). Parathyroid carcinoma at presentation is usually already accompanied by end-organ complications (renal and bone disease) in up to 80% to 90% of patients. The clinical entity of osteitis fibrosa cystica is more often encountered in this group of patients, with more than 40% of them presenting with the characteristic subperiosteal resorption, “salt and pepper” skull, and brown tumors, which may mimic skeletal metastases (236, 254-258). If left untreated, pathological fractures occur in almost 90% of patients (254). In a large single-center retrospective study including Chinese patients with PHPT (259), parathyroid carcinoma patients presented with significantly higher serum calcium and PTH levels and bone turnover markers, and lower BMD at total hip and femoral neck, compared to patients with benign adenoma/hyperplasia.

#### Effect of surgical treatment

Surgery is the first-line treatment for parathyroid carcinoma. The gold standard approach, when malignancy is suspected, is the radical resection of the primary lesion with gross clear margins and excision of any involved structures. However, when the recognition of the malignancy isn't possible pre- or intraoperatively, the management strategy is more complicated, reexploration surgery may be considered, and risk of recurrence is increased (236, 260). HBS is common, especially after total resection of the tumor; thus, serum calcium and PTH levels should be monitored closely (251). It has been proven that skeletal lesions may be reversible after successful complete resection of the tumor: biochemical indices of HPT are normalized after management of hungry bone syndrome; markers of bone formation and BMD especially at hip and lumbar spine increase early after surgery; radiographic findings may be reversed, and in a few cases, brown tumors may also regress (141, 142).

#### Effect of pharmacological treatment

Parathyroid carcinoma patients’ morbidity and mortality arise primarily from hypercalcemia. Patients with severe hypercalcemia and/or hypercalcemic crisis need to be medically stabilized before any surgical operation. In addition, nonoperative tumors or widely metastatic disease bring out the need for medical management of chronic hypercalcemia and the related symptoms. Except from hydration and loop diuretics, multiple agents with transient efficacy regarding hypercalcemia and no efficacy regarding tumor burden have been used: potent bisphosphonates as pamidronate and zoledronate, calcitonin, octreotide, and corticosteroids may achieve only temporary control of hypercalcemia, whereas other agents such as mithramycin, plicamycin, and gallium nitrate are abandoned because of renal toxicity (252). Denosumab has shown sustained efficacy in controlling hypercalcemia in a parathyroid carcinoma patient with severe hypercalcemia and resistance or contraindication in other available agents (196).

##### Cinacalcet

Encouraging data concerning the efficacy of calcimimetic therapy with cinacalcet derive from a multicenter, open-label, single-arm dose-titration study that included 29 inoperable parathyroid carcinoma patients (261). Cinacalcet dose progressively increased up to a tolerated maximum of 360 mg daily, divided into 4 equal doses. A marked and sustained decrease in serum calcium was observed (from 12.7 ± 0.8 mg/dL to 9.9 ± 0.9 mg/dL), although neither PTH levels nor ALP showed significant alteration. In addition, for unresectable/metastatic or recurrent disease, ultrasound-guided percutaneous injection of 98% ethanol into malignant lesions has been shown to temporarily decrease serum PTH and calcium levels (252, 262).

##### Immunotherapy

Immunotherapy is a novel and promising treatment of hypercalcemia in metastatic patients that emerged in 1999, from the work of Bardwell and Harvey (263). They used synthetic human and bovine PTH peptides to trigger the generation of antibodies in a patient with parathyroid carcinoma and metastases, severe bone disease, extreme hypercalcemia, and resistance to conventional treatment. They expected that these antibodies would cross-react with human PTH. This approach aimed to immunologically block the effects of high PTH levels in this patient. Indeed, autoantibody production was reported 1 month after initial immunization, with increasing titers after repeated doses of immunogenic peptides. Marked clinical and biochemical improvement was observed, with total serum calcium concentrations decreasing from 0.5 mmol/L to 1.7 mmol/L over the 6-month period of treatment. Later, Betea et al followed the same protocol in a 50-year-old woman with refractory parathyroid carcinoma and pulmonary metastases (264). They described a decrease and even normalization of calcium and PTH levels after fourth immunization and maintenance of the results for more than 1 year of follow-up. Moreover, they reported a progressive significant decrease in the size of lung metastases to the time of eighth immunization. In a more recent case report, surgery and anti-hPTH immunotherapy were used to treat a patient with CDC73-associated metastatic parathyroid cancer. A 12-year-long remission of diffuse parathyroid carcinoma has been documented after 5 cycles of treatment and subsequent surgery (265).

Immunotherapy is a promising individualized treatment option driven by cancer genomics. The past several decades report only a few case reports that described the administration of immune checkpoint inhibitors in patients with metastatic parathyroid cancer. In the first case, a 46-year-old woman with metastatic disease presented 12 years after the initial diagnosis and resection of parathyroid carcinoma, was treated with pembrolizumab, an anti-PD-1 antibody, leading to biochemical response. Subsequently, she underwent surgical resection of oligometastatic disease of abdominal lymph nodes, experiencing at least 3 years of progression-free survival (PFS) (266). The second case report described a 60-year-old male patient with Lynch syndrome, which is characterized by microsatellite instability that leads to a load of tumor mutations. A 60% decrease in pulmonary metastases and a full biochemical response were documented with pembrolizumab given over the course of only 4 months, due to severe immune-related colitis. The patient's PFS was at least 2 years (267). The third and most recent case described is a 64-year-old female patient with widespread brain and skeletal metastatic disease, 6 months after the first surgical resection, and refractory hypercalcemia (268). After molecular genomics confirmed a high tumor mutation burden, nivolumab immunotherapy was administered, resulting in resolution of hypercalcemia and PFS of at least 1 year.

### Secondary and Tertiary Hyperparathyroidism (SHPT, THPT)

PTH possesses a crucial role in calcium homoeostasis, by aiming to restore serum calcium levels in the state of hypocalcemia through its renal, intestinal, and bone actions. The compensatory rise of PTH levels intended to rectify the lower bioavailability of calcium and/or vitamin D is referred to as SHPT. It may result from failure of 1 or more components of the calcium homoeostatic mechanisms. Although kidney disease is the most common cause of SHPT (269), any disorder that leads to hypocalcemia or vitamin D deficiency may result in elevated levels of PTH: reduced calcium intake or absorption, reduced absorption, synthesis or function of vitamin D and its active metabolites, hepatic failure, and impaired function of PTH are some examples of SHPT etiology. The diagnosis is supported by a detailed clinical history and examination, plus laboratory evaluation of calcium metabolism indices (serum albumin-adjusted calcium and ionized calcium, phosphate, PTH, 25OH-hydroxyvitamin D, ALP). By definition, hyperparathyroidism is characterized by increased levels of PTH. High or low/low-normal serum calcium levels are usually the key for differential diagnosis between PHPT and SHPT, respectively. Moreover, because of the phosphaturic impact of PTH, both PHPT and SHPT are typically linked to low-normal serum phosphate levels in the presence of intact renal function. However, the biochemical values assessed may differ depending on the underlying cause of SHPT (4). Although most signs and symptoms experienced by patients with SHPT are related to their underlying conditions, hypocalcemia (acute or chronic) can also be part of the clinical presentation, especially in severe cases, and patients may experience numbness, tingling, cramps, or even seizures and tetany, as well as complications of long-standing hyperparathyroidism. Chronic kidney disease is an area of major clinical and scientific interest regarding bone metabolism and development of therapeutic options.

Long-standing CKD leads to disruption of the normal regulation and to prolonged stimulation of parathyroid cell proliferation, due to high phosphate, low 1,25-dihydroxyvitamin D, and intermittent hypocalcemia. In addition, the activation of the pro-proliferative signaling pathways of mTOR, TGF-α-EGFR, NF-κB, and Cox-PGE, as well as the disruption of cell division gatekeepers contribute to parathyroid cell proliferation in CKD (270). In SHPT, the diffuse hyperplasia affects all 4 parathyroid glands in contrast to PHPT, where usually a single adenoma is the underlying cause. Hyperplasia causes certain cells to evade cell-cycle regulation and multiply rapidly, resulting in adenomatous growth (formation of tiny nodules that are monoclonal in origin). In rare cases, progressively, a single nodule takes up the entire gland (271). In this condition, which is termed THPT, reduced negative feedback of calcium on PTH secretion, often due to a decreased expression of CaSR in the parathyroid glands, leads to further PTH secretion and calcium elevation, sometimes up to the point of overt hypercalcemia. Tertiary hyperparathyroidism may persist after renal transplantation. A retrospective observational study demonstrated that among 607 patients with successful renal transplantation, 155 (60%) had an elevated PTH 1 year after transplantation and some had also increased serum calcium levels (272). Following renal transplantation, ongoing high levels of PTH can worsen hypercalcemia with intact kidney increasing conversion of 25OH-vitamin D to active 1,25-dihydroxyvitamin D (273).

THPT must be distinguished from PHPT because of their biochemical and clinical similarities: elevated PTH accompanied by elevated serum calcium levels are observed in both situations. However, phosphate levels are mostly elevated in CKD, even though they may be low after kidney transplantation. Signs and symptoms, like with PHPT, are related to the level of PTH and calcium elevation and include soft tissue or vascular calcifications, skeletal pain, decreased BMD, fractures, muscle weakness, nephrolithiasis, peptic ulcer disease, pancreatitis, and changes in mental status (274). Other causes apart from renal disease are uncommon in the pathogenesis of THPT (269). However, it is worth mentioning that other disorders with the same biochemical profile (preexisting or uncorrected PHPT, unmonitored calcitriol oversupplementation, lithium administration) must be excluded. There is still the need for development of evidence-based guidelines for the treatment of THPT and for conduction of large trials comparing possible interventions.

#### Chronic kidney disease and bone

SHPT is the primary cause of morbidity and mortality in patients with CKD, along with cardiovascular and mineral metabolism complications (275). Pre-pro-PTH (115 amino acids long) is the main PTH translational product, which is further enzymatically cleaved to the 84 amino acid long intact PTH (iPTH) and it is then released into the bloodstream and goes through a number of proteolytic changes (276). The concentration of iPTH, which represents the hormone's physiologically active component, is less impacted by glomerular filtration rate (GFR) than its breakdown products (276). Serum iPTH is a valid marker for evaluating SHPT in CKD patients (277). The compensatory elevation of PTH is multifactorial: as GFR declines, circulating calcitriol decreases, both because of renal mass decrease and to inhibition of 1a-hydroxylase by hyperphosphatemia, thus promoting hyperuricemia and metabolic acidosis; PTH1R and a-Klotho receptor (a co-receptor of fibroblast growth factor 23 [FGF23]) expression is decreased in the kidney; phosphate renal excretion is diminished and hypocalcemia is developed with further reduction of GFR; thus, PTH synthesis and secretion is stimulated; resistance to the phosphaturic hormone FGF23 may further increase hyperphosphatemia; iPTH half-life increases and C-terminal fragments of the hormone tend to accumulate; resistance to the circulating calcium and calcitriol at the level of parathyroid glands develops because of hyperuricemia, and may contribute to further PTH increase; multiple signaling pathways lead to parathyroid cell proliferation (270, 275, 278, 279). At early stages of CKD, the action of PTH on both bone and kidney seems to be impaired, resulting in PTH hyporesponsiveness and higher concentrations of serum PTH to balance calcium homeostasis (279). This phenomenon is attributed to multiple factors, including decreased calcitriol levels, phosphate retention, downregulation of PTH1R, and uremic toxins (279).

**Figure 4.**
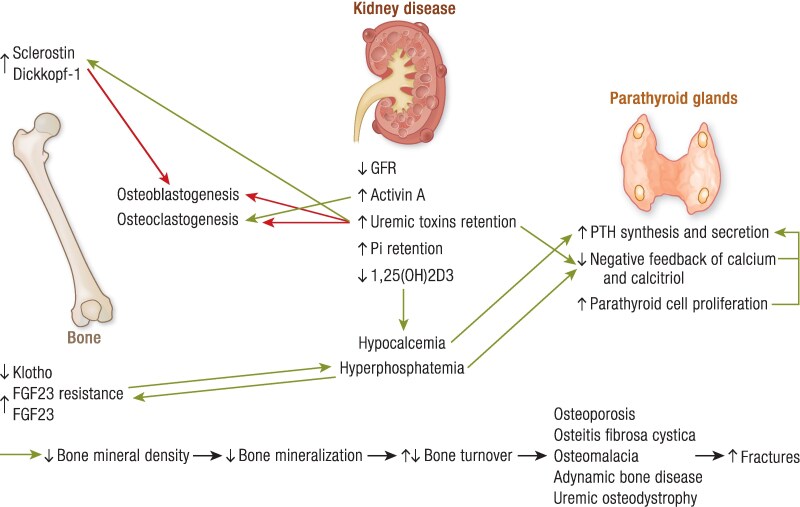
Cascade of events leading to CKD bone and 1,25(OH)_2_D_3_ mineral disorder. During the progression of kidney disease, the elevation of FGF23 and PTH fail to decompensate the decreased renal phosphate excretion; thus, serum phosphate rises. Inhibition of 1-a-hydroxylase synthesis of 1,25-(OH)_2_ vitamin D by FGF23, which leads to a decrease in intestinal calcium absorption, along with the retention of uremic toxins that inhibits osteoclastogenesis and osteoblastogenesis, result in hypocalcemia. Hypocalcemia and hyperphosphatemia further stimulate PTH and FGF23 secretion, creating a vicious circle. Resistance of parathyroid cells to calcitriol- and calcium-mediated regulation of PTH secretion contributes to aggravation of the condition. With time, chronic stimulation of parathyroid cells leads to parathyroid cell proliferation, hyperplasia, or even nodular hyperplasia. PTH stimulates osteoblasts and osteoclasts and increases bone remodeling. However, in CKD, bone remodeling may be decreased due to other mechanisms, such is elevation of sclerostin, Dickkopf-1, activin A, and uremic toxins. BMD is decreased and, depending on the level of bone mineralization and bone turnover state, bone disease may vary from osteitis fibrosa cystica with high bone turnover to adynamic bone disease with low bone turnover. 1,25(OH)_2_D_3_, calcitriol; FGF23, fibroblast growth factor 23; GFR, glomerular filtration rate; Pi, inorganic phosphate; PTH, parathyroid hormone.

Recent data suggest that bone disorder is present from early stages of CKD. Increased levels of Wnt signaling inhibitors (DKK-1, sclerostin, secreted frizzled-related protein), increased activin A (a member of the TGF-β superfamily that is secreted by renal fibroblasts and stimulates osteoclastogenesis), operate along with the accumulation of protein-bound and gut-derived uremic toxins, leading to osteoblast and osteoclast dysfunction and low bone turnover (280). High accumulation of protein-bound uremic toxins seems to inhibit muscle function as well (281). Subsequently, a mild increase of PTH may be understood as an adaptive mechanism to maintain balance in mineral metabolism and bone turnover (280). In late CKD stages, high serum PTH levels overcome peripheral PTH resistance and lead to high-turnover bone disease, albeit the bone newly formed is of poor quality and quantity (281). As CKD progresses, uremic products’ elevation further exacerbates bone turnover and muscle catabolism. Both quantity and quality of bone is reduced as osteoblast and osteoclast lineages are affected (281). The pathophysiology of SHPT and bone disease resulting from CKD is summarized in Fig. 4. Diagnosis relies on the findings of elevated iPTH levels accompanied by low or low-normal calcium levels, normal or elevated serum phosphate, normal or low vitamin D levels and high serum ALP (275). As CKD progresses, PTH levels steadily rise (277, 282). Researchers have described a linear negative connection between serum PTH and serum calcium in grade 3 CKD (277, 283) and between serum PTH and serum 25OH-vitamin D throughout the stages of CKD, which was significant in stages 3b, 4, and 5. The lack of 1,25-dihydroxy vitamin D measurement highlights as a limitation of the study (277). In addition, in grades 4 and 5, a substantial correlation between iPTH and creatinine has been described, whereas in grade 4, a significant negative correlation between iPTH and eGFR has been also reported (277). Although the incidence of SHPT increases with CKD stage, it is thought that SHPT and impaired bone turnover develops from stage 3 CKD and the risk is progressively higher as CKD progresses (284, 285), nowadays, it has been suggested that CKD-mineral and bone disorder (MBD) starts earlier in the course of CKD (277).

As already discussed with PHPT, clinical findings of SHPT tend to be more subtle in the last few decades, due to early medical intervention, careful monitoring, and better control of the biochemical aspects in CKD. However, bone involvement occurs in almost all CKD patients, particularly in the late stages and dialysis stage, and sometimes even after renal transplantation. Bone pain and deformities, myopathy, growth abnormalities, and fractures are some of the skeletal manifestations related to SHPT in CKD (275). The previous term “renal osteodystrophy” refers to the skeletal component of the now-termed CKD-MBD and includes a range of abnormal bone metabolism and morphology due to CKD. Its main manifestations include *osteitis fibrosa cystica* or hyperparathyroidism with high bone turnover, osteomalacia, or adynamic bone disease with low bone turnover or mixed disorder (uremic osteodystrophy) (280, 286). Adynamic bone disease is the most prevalent type of bone disorder in CKD-MBD, even in early stages of CKD. Older age, higher prevalence of diabetes mellitus, malnutrition, gonadal dysfunction, calcium overload, and the use of antiparathyroid medications are some of the causes of the increasing prevalence of adynamic bone disease (280). It is characterized by decreased bone turnover with a predominance of bone resorption and delayed remodelling, which permits more secondary mineralization, leading to a decreased bone toughness and increased risk of fractures. Bone turnover markers (ALP, bone-specific alkaline phosphatase [BSAP], P1NP, DKK-1, tartrate-resistant acid phosphatase 5b [TRAP5b]) are decreased; however it should be noted that only BSAP, intact P1NP, and TRAP5b are reliable in patients with CKD because they are not renally cleared. Serum ALP and intact or bio-intact PTH levels mirror quite well the underlying bone turnover state; combined measurement of these 2 biomarkers can be used to evaluate bone disease in CKD patients and drive the therapeutic and follow-up strategies (280).

PTH excess is believed to decrease bone strength through cortical bone thinning. Kazama et al compared parathyroid function and cortical bone thickness in biopsied iliac samples in 47 dialysis patients (287). They concluded that in patients with intact PTH levels >1000 pg/mL there was an inverse correlation of PTH with cortical bone thickness. In addition, higher PTH levels were related to more irregular cancellous bone surface profiles. Bacchetta et al showed, using HR-pQCT imaging, that the trabecular microarchitecture is also affected (288). Of note, they reported a moderate but significant trabecular microarchitecture impairment in early predialysis CKD patients. It is well accepted that both very low (less than 2 times the upper limit of normal) and very high (more than 9 times the upper limit of normal) PTH levels, that are respectively associated with low or high-turnover bone disease, increase mortality in dialysis patients (289).

Although the gold standard for the diagnosis of renal osteodystrophy is the histomorphometric analysis obtained with bone biopsy, this is usually not part of the clinical practice because it is an invasive procedure (275). BMD assessed by DXA is markedly decreased at radius and to a lower extent at vertebrae (290). A study in hemodialysis patients with SHPT showed that BMD at distal 1/3 of radius was significantly lower than in controls, whereas it was negatively correlated with PTH and significantly decreased with duration of dialysis (291).

Fracture is an important clinical outcome in CKD patients, presenting with a significantly elevated rate in comparison to the general population, and is related to excess morbidity and mortality (292, 293). Except from SHPT, decreased calcitriol levels, hypocalcemia, phosphate retention, changes in bone architecture, nutritional disturbance, increased oxidative stress, hemodialysis-associated amyloidosis, and adynamic bone are some of the suggested pathophysiological conditions related to fracture susceptibility in this group of patients (285). The risk of fragility fracture is increased across the spectrum of the disease, affecting both predialysis (294) and dialysis patients (295) in a progressive manner; more specifically, the risk is up to 5-fold higher in patients with GFR of <15 mL/min/1.73 m^2^ compared to those with GFR >60 mL/min/1.73 m^2^ (280). The risk of hip and nonvertebral fractures is higher than the risk of vertebral fractures among CKD patients (296, 297). In contrast to what has been previously demonstrated (275, 298, 299), the last decade, well-supported data including a meta-analysis (300-304), suggest that BMD can discriminate fracture risk among CKD patients as well, with hip BMD being the most reliable for fracture prediction among hemodialysis patients (305). Therefore, BMD has been adopted by Kidney Disease Improving Global Outcomes guidelines to evaluate patients with CKD grade 3a-5 (289). In addition to BMD, TBS score is another useful tool toward frailty assessment and fracture risk prediction (306, 307).

#### Effect of pharmacological treatment on CKD-MBD-related bone disease

Advances in the treatment of SHPT in CKD aim at slowing disease progression and minimizing complications, through maintaining serum phosphorus, calcium, and PTH within accepted ranges. Phosphate balance, especially for dialysis patients, may require more measures than restriction of dietary phosphate and protein intake. Inorganic phosphate binders and agents that directly block intestinal phosphate absorption are used for reducing intestinal ingestion of the mineral in persistent hyperphosphatemia (275). Controlling hyperphosphatemia inhibits PTH secretion in CKD patients with mild to moderate disease as well (308). In addition, lowering the calcium concentration of dialysis fluid between 1.25 and 1.50 mmol/L was reported to improve bone and mineral parameters and may help prevent hypercalcemia in patients with CKD (289). Apart from the management of biochemical SHPT/THPT with vitamin D analogues and calcimimetics in CKD, pharmacological treatment with antiosteoporotic agents such as bisphosphonates, denosumab, raloxifene, and teriparatide have been administered when bone disease is advanced.

##### Vitamin D and analogues

More than 50% of hemodialysis patients have total serum 25-hydroxy vitamin D deficiency (309). Native vitamin D supplementation (vitamin D2, vitamin D3) has been demonstrated to be effective in controlling SHPT of CKD in early-stage patients. Six-month supplementation with ergocalciferol was reported to increase serum 25-hydroxyvitamin D levels in patients on hemodialysis with vitamin D insufficiency or deficiency but had no effect on SHPT (310). Long-term cholecalciferol supplementation has been reported to significantly improve vitamin D insufficiency/deficiency in predialysis CKD patients, improve calcidiol, and to a lesser extent calcitriol concentrations, and decrease PTH levels without adverse effects on serum minerals (311). In a recent systematic review and meta-analysis of 128 RCTs (312), Yeung et al concluded that the administration of vitamin D in patients with stage 3, 4, and 5 CKD and CKD-MBD lowers serum PTH and ALP; however, it increases serum calcium levels and moreover, the results regarding the effects on fracture risk are uncertain. There is still no universal approach to the management of vitamin D deficiency and SHPT. Although guidelines vary regarding the optimal levels and the goal of supplementation, it is generally accepted that vitamin D deficiency should be prevented; the National Kidney Foundation recommends that CKD patients maintain 25-hydroxyvitamin D levels above 30 ng/mL and below 60 ng/mL and suggests the daily intake of 4000 IU of vitamin D as the maximum dose (313).

Conversion of 25-hydroxyvitamin D to 1,25-dihydroxy-vitamin D may be diminished due to renal impairment and the related decreased 1a-hydroxylase activity, as well as due to FGF23 increase and uremia. Vitamin D receptor activators (VDRAs) are a cornerstone in the control of SHPT in CKD, giving that active vitamin D deficiency has a major role in the pathogenesis of the disorder. Calcitriol, the first IV synthetic physiological VDRA, has been successfully used to suppress PTH secretion by reducing gene transcription in parathyroid cells and inhibiting parathyroid gland hyperplasia and by promoting negative feedback in the production and secretion of PTH, through VDR and CaSR expression (314, 315). After calcitriol, multiple active vitamin D analogs have been developed (alfacalcidol, paricalcitol, 22-oxacalcitriol, doxercalciferol). Of note, in hemodialysis patients with SHPT, 22-oxacalcitriol exerted greater suppression on bone turnover markers, such as BSAP, pyridinoline, osteocalcin, and NTX, as compared to calcitriol (316). On the other hand, doxercalciferol was equivalent to calcitriol in suppressing bone formation markers without correcting mineralization defects in pediatric patients under peritoneal dialysis (317). Despite their similar efficacy in controlling SHPT and reducing the incidence of cardiovascular events and proteinuria in predialysis CKD patients (318), vitamin D analog administration is associated with a 6.6-fold greater risk for hypercalcemia compared to placebo (319). Therefore, VDRA administration requires frequent calcium monitoring and is not routinely recommended in all predialysis patients but only in those with severe and progressive SHPT (289, 313, 320).

In conclusion, vitamin D deficiency should be prevented in CKD patients even though native vitamin D supplementation has a minor effect in CKD-MBD-related bone damage. Conversely, VDRAs play a key role in controlling SHPT, inhibiting PTH and bone turnover markers; however, they increase the risk of hypercalcemia.

##### Calcimimetics

Calcimimetics allosterically modify the CaSR in the parathyroid gland, increasing sensitivity to calcium levels, thus reducing PTH. Calcimimetics can reduce serum PTH, calcium, and phosphorus levels; increase BSAP; and diminish osteocalcin and the rate of parathyroidectomy in SHPT CKD patients without increasing the rate of total adverse events (321). Oral cinacalcet therapy with or without vitamin D analogs are recommended in patients with end-stage CKD (289), as multiple studies have proved its efficacy and safety in controlling SHPT in patients undergoing dialysis (322-324). The indications for cinacalcet therapy in parathyroid disorders are included in Table 4. The multicenter study BONAFIDE (325) assessed the skeletal response to cinacalcet in adult dialysis patients with SHPT and high turnover bone disease. Long-term treatment with cinacalcet resulted in decreased PTH and bone turnover, and generally improved bone histology. Although the large control clinical trial EVOLVE didn't meet the primary endpoints of reduction of all-cause mortality and major cardiovascular event risk (326), the secondary analyses showed that cinacalcet demonstrated significant reduction in the risk of severe unremitting SHPT in dialysis patients. Moreover, post hoc unadjusted intention-to-treat analyses showed that cinacalcet has no effect on the risk of clinical fractures, although it did show a substantial reduction in the risk of fracture when analyses were adjusted for age: it favored the older ones (327). In a retrospective cohort study including 25 hemodialysis patients with SHPT (328), 1-year treatment with cinacalcet increased BMD at the femoral neck by 7.3%, whereas in the control group, there was a reduction of 6.2%. In the cinacalcet group, serum ALP levels but not PTH levels were significantly negatively correlated with the changes in BMD. Furthermore, a randomized, double-blind, placebo-controlled study (329) has shown that cinacalcet is also effective in controlling SHPT in nondialysis patients. In addition to the predialysis state, there are some preliminary data suggesting that treatment with cinacalcet is beneficial in controlling persistent SHPT after renal transplantation (330). Hence, more data are required to receive approval for extended use in these latter conditions.

The relatively low compliance of patients seen with cinacalcet due to the gastrointestinal side effects, may be overcome with the intravenous administration of etelcalcetide, a novel second generation calcimimetic drug, recently approved for the management of SHPT in hemodialysis CKD patients (331, 332). Etelcalcetide acts as direct CaSR agonist, has longer half-life and is administered IV 3 times per week at the end of the dialysis treatment. Numerous studies (333-338) have demonstrated the long-term efficacy of this calcimimetic agent in reducing PTH, FGF23, calcium, and phosphorus levels in dialysis SHPT patients at all grades of disease severity. Compared to cinacalcet, etelcalcetide has demonstrated similar or even superior PTH- and calcium-reducing potency (339, 340). Therefore, despite the long-term safety profile of the drug (338, 341), clinicians have to exercise vigilance regarding the possible side effect of hypocalcemia and the associated prolongation of the QT interval (339). Concerning bone microarchitecture, etelcalcetide reduced bone turnover, improved mineralization, decreased marrow fibrosis, and preserved cortical bone structure and bone strength by lowering PTH in subtotal nephrectomy rats with established SHPT (342). Its novel-found ability of increasing sclerostin levels remains to be clarified and associated or not with alterations in bone metabolism (343). In a recently published prospective trial, 22 patients with SHPT on hemodialysis ≥1 year received etelcalcetide for 36 weeks and were evaluated regarding their spine areal BMD, trabecular bone quality, bone turnover, and bone material properties using DXA, TBS, high-resolution pQCT, and bone biopsy, respectively (344). Treatment with etelcalcetide was associated with significant PTH decrease, BMD increase at all sites, spine TBS increase, and decrease of bone formation rate, maintaining the mechanical properties of the radius (stiffness and failure load).

Evocalcet is a novel oral calcimimetic agent, developed and approved in Japan for the management of SHPT in dialysis patients. It has demonstrated a favorable profile regarding gastrointestinal side effects and exhibits PTH-lowering effects with lower doses (345). Administration of evocalcet in single doses of 1 to 20 mg produces a dose-dependent effect on serum PTH, calcium and phosphorus levels (346). Subsequent trials have confirmed the long-term efficacy and safety of the agent both in hemodialysis and peritoneal dialysis SHPT patients (347-349).

Calcimimetics have a pivotal role in the management of end-stage CKD bone disease, due to their efficacy in decreasing PTH, calcium and phosphorus levels and reducing bone turnover, both in dialysis and predialysis patients. They should be used with caution because of the possible side effect of adynamic bone disease and hypocalcemia. There are few data concerning their effect on BMD and fracture risk.

##### Bisphosphonates

Because of their low lipophilicity, bisphosphonates are poorly absorbed from the gastrointestinal tract (1%-5%), having a further reduced bioavailability if not taken as indicated (fasting with a glass of water and least 30 minutes before any food or fluids) (350). They are absorbed through paracellular transport and then partially bound in the hydroxyapatite in bone, which are liberated again after bone resorption and partially eliminated unchanged by renal excretion (350, 351). Trace amounts of bisphosphonates may be found in the urine for up to 8 years following treatment discontinuation (352), with alendronate having a half-life of more than 10 years in bone (351). In CKD, bisphosphonates have a progressively prolonged half-life, possibly leading to accumulation in soft tissue, with ambiguous clinical impact (353). Although animal and numerous clinical studies have reported that bisphosphonates may cause direct nephrotoxicity (acute tubular necrosis, acute renal impairment, nephrotic syndrome, focal glomerulosclerosis), especially if administered IV (354-358), it is an uncommon adverse event and is minimized with adjustment of the dosage and prolongation of the infusion time (350). Their use is generally contraindicated in GFR <35 mL/min/1.73 m^2^ and it has been proposed to diminish their dosage by 50% when given in patients with CKD stage 4-5 (359). Antiresorptive drugs may encourage the development of adynamic bone disease, which may increase fracture risk, a concern that has to be addressed when treating patients with end-stage CKD. Amerling et al found adynamic bone disease on bone biopsy evaluation in all of 13 patients with osteoporosis and CKD stage 2-4 that they studied (360). In animal models with CKD, the rate of bone turnover has no significant impact on the amounts of zoledronate accumulation or retention (361).

Although the presence of renal impairment has historically been a universal exclusion criterion, numerous patients with CKD (as calculated by the Cockcroft-Gault method) have been included in large research studies on the effectiveness of bisphosphonates, proving generally a safe profile (362, 363). In a recent systematic review (364), 6 trials including CKD patients (n = 1013), compared bisphosphonates to placebo in terms of their effects on BMD, fractures, and safety. Two of these studies were carried out in CKD stages 3-4, having inconclusive results, and 4 were carried out in renal transplant recipients; 3 of them demonstrated that alendronate or pamidronate reduced bone loss after transplantation, whereas the fourth showed no benefit on lumbar BMD but a slight increase in total femur BMD with ibandronate. A post hoc analysis of 3 phase III multicenter, randomized, double-blind, controlled trials including 852 patients with CKD and eGFR ≥ 30 mL/min/1.73 m^2^ showed that treatment with risedronate (either 2.5 mg once daily or 17.5 mg once weekly) is safe and effective regardless of the CKD stage, leading to significant increase in lumbar spine BMD and a significant decrease in bone turnover markers (urine NTX, urine CTX, and BSAP) after 48 weeks (365). Of note, these changes didn't vary significantly between the different CKD stages. Another smaller post hoc analysis including 420 patients with osteoporosis and eGFR ≥30 mL/min/1.73 m^2^ demonstrated that a treatment regimen of once-monthly risedronate 75 mg is also safe and has favorable effect in lumbar BMD and bone turnover markers in a comparable extent between the CKD stages (366).

When 13 dialysis patients with SHPT (PTH > 500 pg/mL) and osteopenia received 60 mg of pamidronate IV every 2 months for 1 year in a prospective study, they exhibited increase in both lumbar and femoral neck BMD at 6 and 12 months of treatment (367). PTH increased at 3 months in all patients and decreased markedly in 10 patients after increasing the calcitriol doses, whereas no significant decrease in calcium, phosphate, and ALP levels was observed. In a systematic review of 7 randomized placebo-controlled trials, including 7428 participants with CKD stage 1-4 (368), ibandronate was proven safe and effective in increasing BMD at the hip and lumbar spine throughout the spectrum of CKD and in decreasing the risk of all fractures, albeit nonsignificantly, except for stage 3b, where the risk was increased. In an RCT including 31 hemodialysis patients with moderately elevated iPTH levels (100-300 pg/mL), 6-week alendronate therapy (40 mg once weekly) was well tolerated and preserved hip BMD, whereas it led to a significant reduction of osteocalcin levels only after 1 month of treatment (369). In conclusion, according to Kidney Disease Improving Global Outcomes guidelines (289), patients with osteoporosis and/or high fracture risk and CKD stages 1-3b with normal PTH should be treated similarly to the general population, whereas in CKD stages 3-5 and CKD-MBD, patients’ treatment should be individualized depending on biochemical indices and possibly bone biopsy results.

After renal transplantation, in patients with persistent SHPT (n = 24), antiresorptive therapy with alendronate inhibited bone turnover and decreased bone turnover markers; nevertheless, PTH levels increased, bone mass remained unchanged, whereas the incidence of new clinical fractures increased (370). BMD also remained unchanged after ibandronate treatment in a retrospective, matched case-controlled study of 60 renal transplant recipients (371). In another retrospective study including 85 patients after transplantation (372), bisphosphonate treatment increased BMD both in femoral neck and in lumbar spine; however, treatment with denosumab was comparatively superior.

In summary, post hoc analyses and multiple studies have shown that bisphosphonate treatment is safe when individualized, may have favorable effect on BMD and reduce turnover in CKD patients, in dialysis SHPT patients, as well as in renal transplant recipients. However, data regarding fracture risk are sparse.

##### Denosumab

Using eGFR, a post hoc analysis of the FREEDOM study included 7801 patients CKD stage 1-4 (88% were stage 1-2). Patients receiving denosumab had higher BMD at lumbar spine, femoral neck, and total hip, and reduced risk of fracture across kidney function compared to placebo, both at the end of the 3-year follow-up and at the 10-year extension study (373, 374). It has been proven that renal function has no discernible impact on the pharmacokinetics of denosumab (375). A retrospective longitudinal study has been recently published, assessing the efficacy and safety of denosumab in postmenopausal women with mild-to-moderate CKD and PHPT (188). The researchers demonstrated that denosumab treatment was safe, significantly increased BMD at all skeletal sites after 24 months of treatment and mediated a significant calcium-lowering effect. Simonini et al described the results of 12 trials that examined the effects of denosumab (60 mg every 6 months) in dialysis patients (n = 461) (376). Each study involved a small number of patients (between 12 and 124) and had a short follow-up period (between 6 and 24 months); therefore, the efficacy of denosumab in fracture risk couldn't be assessed. In all studies included, except from 1, denosumab was found to have a positive impact on BMD, regardless of the bone turnover status; however this effect was more pronounced in individuals with elevated serum bone turnover markers (ALP and TRAP5b). The positive effect of denosumab in adynamic bone disease may imply that in spite of antiresorptive treatment, osteoblasts may regain their functional ability (376). Possible mechanisms suggested include the promotion of PTH secretion and bone metabolism by a drop in serum calcium or the direct promotion of bone modeling by denosumab, as observed in primate models of osteoporosis (376, 377). Iseri et al retrospectively studied 124 dialysis patients for a maximum of 5 years of denosumab therapy. They concluded that both areal and volumetric BMD and the estimated strength parameters of the whole hip were significantly higher during a period of 3 to 4 years, ultimately reaching a plateau with denosumab treatment (378); these gains were diminished following the cessation of treatment.

A few studies have been conducted focusing on the effect of denosumab on bone mass of CKD patients with SHPT. An open-label prospective clinical study (379) showed that denosumab is effective in increasing BMD in patients with SHPT and low bone mass undergoing dialysis, both at the femoral neck and lumbar spine. In addition, patients taking denosumab experienced decreased levels of PTH, calcium, and bone pain. Hypocalcemia is a major side effect of denosumab, particularly seen in patients with SHPT and high bone turnover. Bird et al recently highlighted the great and clinically significant risk of severe hypocalcemia in older dialysis patients (191). Specifically, they reported that dialysis patients aged >65 years treated with denosumab for osteoporosis had a significantly higher risk of severe (<1.8 mmol/L or 7.5 mg/dL) and very severe (<1.6 mmol/L or 6.5 mg/dL) hypocalcemia compared to their counterparts treated with oral bisphosphonates (41.1% vs 2% and 10.9% vs 0.4%, respectively). Thus, close monitoring of serum calcium is recommended to prevent severe hypocalcemia and provide adequate treatment (379, 380).

Wada et al (381). described the administration of denosumab in a renal transplant recipient with persistent SHPT and severe bone disease. They proved that denosumab was effective in improving bone loss soon after initiation and allowed the administration of vitamin D in high dose to control SHPT and prevent hypocalcemia.

In conclusion, denosumab is effective in increasing BMD at all sites in CKD patients, regardless of their bone turnover status. In SHPT, denosumab increases femoral neck and lumbar spine BMD and decreases PTH; however, it carries the risk of severe hypocalcemia. Larger studies are urgently required to evaluate the efficacy and safety of denosumab in SHPT CKD patients, as well as to address the concern of increased fracture risk after discontinuation of denosumab in such patients.

##### Raloxifene

Because SERMs seem to have a prolonged plasma elimination half-life in patients with CKD, they need to be used carefully (382). However, they are less likely to cause adynamic bone disease in such patients than bisphosphonates (383). They are safe and effective in increasing BMD at both the hip and the spine and in reducing the risk for vertebral fractures among individuals with CKD (382). In a prospective, blind, placebo-controlled, randomized study, including 25 postmenopausal women on chronic dialysis with severe osteopenia or osteoporosis (384), patients with CKD who received raloxifene 60 mg per day exhibited significantly increased lumbar spine BMD compared to controls, 1 year after initiation of treatment, whereas femoral neck BMD did not change significantly. In addition, serum pyridinoline levels significantly decreased after 6 months. In a more recent systematic review and meta-analysis of 5 RCTs and prospective studies with a total of 244 participants with end-stage CKD and a median duration of raloxifene treatment of 12 months, there was a significant improvement in lumbar BMD in the raloxifene group compared with the placebo group (385). No significant difference concerning the improvement of femoral neck BMD, PTH, calcium, phosphorus, or BSAP was reported. Moreover, it has been documented that raloxifene treatment doesn't affect PTH levels in end-stage CKD and dialysis patients with SHPT (386).

In conclusion, although raloxifene seems to increase lumbar BMD in CKD patients, it has no effect on femoral BMD and biochemical indices of CKD-MBD. These data are of low quality, and as raloxifene has been associated with increased risk of thromboembolism, larger studies are needed to determine whether it is safe and effective in end-stage CKD patients.

##### Teriparatide

Teriparatide, a recombinant human PTH 1-34 (rhPTH [1-34]), is a bone-forming agent approved for osteoporosis treatment in postmenopausal women, as it increases BMD and decreases fracture risk by stimulating osteoblast activity (387). Given its hepatic clearance and its anabolic effects, it could be a safe and valuable therapeutic agent in CKD-MBD, with greatest theoretical benefit in those with adynamic bone disease (or by surrogate measures, those with low bone remodeling markers). However, in CKD patients, who typically have lower BMD at the radius, teriparatide-related reduction in radial cortical BMD may lead to clinical implications of increased fracture risk (although this has not been demonstrated in studies). Additionally, it is unclear whether teriparatide has anabolic effect in CKD patients with PTH resistance or SHPT.

A subanalysis of the Fracture Prevention Trial (388), which included 736 postmenopausal women with osteoporosis, creatinine concentrations ≤2.0 mg/dL, and normal serum PTH and calcium levels, found that a median 19-month course of teriparatide treatment (both at a dose of 20 mcg/day and 40 mcg/day) mediated statistically significant increases in lumbar spine and femoral neck BMD and significantly raised P1NP serum concentrations across renal function (eGFR from 30 to ≥80 mL/min/1.73 m^2^). Furthermore, it resulted in reductions in the incidence of both vertebral and nonvertebral fractures, in both groups of patients with renal impairment (GFR < 80 mL/min) and of those without (GFR ≥ 80 mL/min). All groups of renal function experienced similar rates of adverse side events, with the exception of patients with moderate renal impairment who had greater rates of hyperuricemia and hypercalcemia. Similar results were found in a small Japanese post hoc analysis of a prospective, multicenter study including 33 patients with CKD stage 4 (n = 30) or 5 (n = 3), of a total of 1882 patients (389). However, according to another subanalysis (390), sequential teriparatide followed by alendronate might not be as successful in preventing nonvertebral fractures in patients with CKD stage 3b-4 (112 patients) as alendronate monotherapy was, although it was more efficacious than alendronate alone at preventing vertebral fractures in patients with CKD stage 1-2 (556 patients). Although the mechanism mediating these different effects remains uncertain, it is implied that cooccurrence of SHPT in CKD stage 3b-4 group (increased serum osteocalcin, P1NP, and TRAP5b levels) might be responsible for this difference.

Although teriparatide treatment can increase trabecular BMD and decrease fracture risk, it is discouraged in cases of CKD-MBD and SHPT. There are only a few reports of its use in CKD and dialysis patients. In a rat model of adenine-induced CKD and SHPT (391), the researchers compared the CKD group (vehicle administration), the teriparatide group (30 μg/kg, 3 times/week), the etelcalcetide group (0.6 mg/kg, daily), and the combination group (teriparatide and etelcalcetide) 8 weeks after the initiation of treatment; teriparatide and combination group exhibited restoration in trabecular bone volume and increased bone strength, the teriparatide group exhibited dramatically reduced bone resorption markers and increased trabecular bone volume, whereas the combination group exhibited significantly decreased bone marrow fibrosis and lipogenesis, increased bone formation, bone volume, and cortical bone thickness and strength. The effect of teriparatide treatment on serum blood urea nitrogen and creatinine was insignificant. In a previous CKD stage 4 rat model study, it was clearly demonstrated that teriparatide has anabolic effect also in SHPT cases, based on dynamic bone histomorphometric data and bone volume, mineralization, and microstructure data (392). These results suggest that teriparatide may sustain the metabolic turnover from a condition of bone resorption dominance to a state of bone formation dominance, both in patients with controlled SHPT under etelcalcetide treatment and in SHPT conditions.

In a small multicenter prospective observational study, during a 12-month period, 15 hemodialysis patients with low PTH (iPTH < 60 pg/mL) and BSAP (<20 ng/mL), which meet the criteria for adynamic bone disease, received a weekly subcutaneous injection of teriparatide 56.5 μg following the first dialysis session of the week (393). At 6 and 12 months, the teriparatide group's lumbar spine BMD rose by 3.7% and 2.5%, respectively. In comparison to the control group, the teriparatide group's change rates of both BMDs tended to be higher. At 1 month following therapy, bone formation markers (BSAP and osteocalcin) rose and remained high until 6 months, whereas bone resorption markers (TRAP5b and NTX) tended to decline from baseline to 6 months in the teriparatide group, before eventually reverting to baseline. Following bone turnover activation, endogenous PTH levels are increased (393). Palcu et al reported a case of a dialysis patient with low bone turnover (proven by histomorphometry) and multiple fractures, including painful pelvic fractures (394). Treatment with daily 20 μg of teriparatide for 24 months improved the patient's physical function, without affecting serum calcium and phosphate levels or the lumbar spine BMD, whereas the femoral neck and total hip BMD increased by 4% and the static and dynamic characteristics of bone improved as shown by an additional transiliac bone biopsy. However, data regarding safety and efficacy of teriparatide in CKD-MBD still remain weak and sparse.

Teriparatide has a favorable profile in terms of improving trabecular BMD, enhancing bone formation markers, and decreasing bone resorption markers in CKD patients. Therefore, it may be able to enhance turnover state in favor of bone formation, which is very helpful in adynamic bone disease. However, the conflicting findings of fracture risk might indicate that the beneficial effect of teriparatide in lowering fracture risk is hampered by the rising prevalence of SHPT as CKD worsens.

##### Romosozumab

Van Buchem disease (sclerosteosis) is a hereditary sclerosing bone dysplasia, caused by loss-of-function mutations of the SOST gene, which codes the protein sclerostin (395). The unravelling of the pathophysiology and the long-term observation of the disease has led to the development of romosozumab, an anti-sclerostin antibody medication, that has recently been approved for the treatment of severe osteoporosis in postmenopausal women (396). Sclerostin downregulates osteoblastogenesis and stimulates osteoclastogenesis and therefore, the inhibition of its actions has a dual positive effect on bone metabolism (397). As mentioned, elevation of sclerostin is 1 of the pathophysiological components of bone turnover dysregulation in CKD-BMD and thus might be considered a possible target for therapy.

Preliminary data have emerged regarding the efficacy and safety of romosozumab in patients with CKD, both at earlier stages and at the hemodialysis stage. A post hoc analysis of 2 randomized, multicenter, phase 3 clinical trials (FRAME and ARCH study) investigated its effect in postmenopausal women with osteoporosis and mild-to-moderate CKD (eGFR 30-90 mL/min/1.73 m^2^) (398). In this analysis, authors reported that romosozumab 210 mg, administered subcutaneously once monthly for 1 year, is effective in osteoporosis treatment for patients with mild-to-moderate CKD, compared with placebo or alendronate; significant improvement of BMD at all skeletal sites was recorded across all levels of kidney function. In addition, the relative risk of new vertebral and nonvertebral fracture was significantly decreased both in FRAME and ARCH study. It must be noted that kidney function remained unchanged during the 1-year period of romosozumab treatment in both studies. However, patients with reduced eGFR in these studies did not have other biochemical features of CKD-MBD. This makes the study less representative of patients with CKD-MBD, but perhaps more representative of age-associated declines in eGFR alone.

In a recent single-center, nonrandomized observational study, researchers evaluated the efficacy and safety of 1-year romosozumab administration (210 mg monthly), in 76 hemodialysis patients with osteoporosis, high risk of fracture, and PTH levels of 152.3 ± 172.0 pg/mL (399). They reported an increase of BMD at lumbar spine and femoral neck in a time-dependent manner, reaching 15.3% and 7.2% increases, respectively, during 1 year of romosozumab treatment. The concomitant increases in bone ALP and total P1NP and the decrease in serum TRAP5b reflected that BMD increase is attributed both to stimulation of bone formation and inhibition of bone resorption early during the treatment. The fracture rate between the treated and the untreated group was not significantly different (3.1% and 5.5%, respectively). However, given that the romosozumab group had a significantly lower baseline femoral neck BMD, the lack of significant difference between the groups may reflect a protective role of romosozumab regarding fractures. No symptomatic hypocalcemia was recorded. In addition, Suzuki et al have demonstrated the clinical effectiveness of romosozumab in a young patient with severe osteoporosis and high bone turnover, who was on hemodialysis because of autosomal dominant polycystic kidney disease (400). After 10 months of romosozumab treatment, in a once-monthly dose regimen, they reported a significant improvement of bone markers and BMD. Nevertheless, it is still unknown whether coadministration of romosozumab with a calcimimetic drug in hemodialysis patients is safe and effective. Ogata et al administered romosozumab to a postmenopausal woman with osteoporosis and SHPT who had been in long hemodialysis and was receiving evocalcet (401). After 1 year of treatment (210 mg once per month), the patient's BMD markedly rose by 32.1% at the spine, 34.9% at the right femoral neck, and 19.2% at the left femoral neck. No cardiovascular disease (CVD) incidents were reported during the course of romosozumab treatment. However, 4 months following the start of romosozumab, albumin-corrected blood calcium levels dropped from 8.6 to 7.9 mg/dL, and intact PTH levels rose from 136 to 685 pg/mL in response.

In a prospective observational study, researchers enrolled 13 treatment-naïve patients with osteoporosis who were on dialysis (402). First, they treated them with romosozumab (210 mg subcutaneously once monthly) for 1 year and subsequently with denosumab (60 mg subcutaneously every 6 months) for 1 year, and they observed significant increases in BMD at all sites, both at the first (9.0% at lumbar spine, 2.5% at total hip, and 4.7% at femoral neck) and the second year of treatment (14.9% at lumbar spine, 5.4% at total hip, and +6.5% at femoral neck). No fractures were recorded during the time of observation. Nonetheless, the potential increase of CVD risk associated with the use of romosozumab requires caution when administering the medication, especially in patients with positive CVD history.

In conclusion, data from the large studies of romosozumab imply its safety and positive effect in increasing BMD at all sites and in reducing fracture risk in CKD patients. Preliminary studies suggest that this effect may be extended to SHPT. However, more data are needed to meet the prerequisite for approval for the use in this group of patients.

#### Effect of PTX on SHPT-related bone disease

PTX is usually a treatment strategy of last resort, after pharmacotherapy has failed (Table 5) (403). It is not yet completely understood whether PTX or renal transplantation should be chosen first in severe disease or poorly treated patients (275). Successful PTX can yield a marked, immediate, and persistent reduction in PTH levels (404, 405) and may ameliorate clinical symptoms such as bone pain (406). Along with PTH reduction, it has been demonstrated that PTX leads to significant decrease of bone resorption markers (calcitonin, type I collagen cross-linked C-telopeptides, TRAP5b) and significantly increases bone formation markers (osteocalcin, ALP) compared to preoperative levels in dialysis patients, suggesting that PTX could promote osteoblast activity and reduce osteoclast activity (407). BMD both at femoral neck and lumbar spine is notably improved after PTX (408) and the improvements are sustained for up to 1 year postsurgically (404). Siqueira et al in their study observed an increase in caloric intake, body weight, body mass index, handgrip strength, along with partial correction of mineral disorder and bone markers, 6 months after PTX in 30 patients with CKD and SHPT (409). It remains to be investigated whether these body composition changes are clearly associated with PTX and whether they contribute to reduction of frailty and risk of falls in these patients. Recently, the PROCEED pilot open-label randomized trial compared the effect of total PTX vs oral cinacalcet on BMD in 65 dialysis patients with advanced SHPT (410). After 12 months, total parathyroidectomy increased the BMD in both femoral neck and lumbar spine and reduced the osteopenia/osteoporosis proportion of participants significantly more than cinacalcet. Notably, no changes in BMD of the distal radius was observed with either intervention.

**Table 5.** Indications for parathyroidectomy in dialysis patients with SHPT (405)

Indications for parathyroidectomy in dialysis patients with SHPT as proposed by Rodriguez-Ortiz et al
Hyperparathyroidism resistant to calcimimetic administration*^a^* Severe refractory hyperphosphatemia Severe hyperparathyroidism in dialysis without response to medical treatment Cases of calciphylaxis with PTH levels above 500 pg/mL that do not rapidly respond to calcimimetics Complications derived from SHPT*^b^* Primary hyperparathyroidism in patients with CKD

Abbreviations: CKD, chronic kidney disease; SHPT, secondary hyperparathyroidism.

^a^Failure to reduce PTH levels by vitamin D analogs or calcimimetic therapy administration.

^b^For example, tendinous rupture, severe bone pain, or refractory anemia.

Further well-designed, long studies, with a larger number of patients to compare the long-term effects of the various treatment strategies on BMD and fracture risk are missing.

#### Effect of renal transplantation on bone disease

Bone disease in renal transplant recipients is a complex multifactorial entity. Although it seems reasonable that the rapid improvement of kidney function after transplantation leads to inversion of pathophysiologic mechanisms of osteodystrophy, posttransplant bone and mineral disease may result not only from the underlying bone disease before transplantation, but also from long-term use of immunosuppressant therapy and from failure of normalization of kidney function or progressive loss of function of the kidney transplant (411, 412). In addition, advanced age, postmenopausal state, previous diabetes mellitus, long time on dialysis, or pretransplant stress fracture may exacerbate the already negative effect of persistent SHPT on bone metabolism after renal transplantation.

The biochemical markers of SHPT demonstrate distinct patterns after kidney transplantation: PTH levels decrease significantly during the first 3 months and tend to stabilize at elevated levels after 1 year; low calcium levels tend to increase and stabilize at the higher end of the normal range within 6 months; phosphate decreases rapidly after transplant to within or below normal levels (413); and FGF23 levels decrease but appear persistently increased as an adaptive mechanism to hyperphosphatemia due to reduced kidney function (411). Hyperparathyroidism may persist in up to 60% of transplant recipients the first year of transplantation, especially in those with previous SHPT or THPT and long duration of dialysis (411). In addition, de novo hyperparathyroidism may rise as a compensatory response to phosphate and calcium metabolism dysregulation, in cases of decline in transplant function (411).

Previous studies have shown that bone mass loss develops rapidly within the first 6 months after kidney transplantation and is followed by a slowdown (273). The frequency of high-turnover bone disease decreases after transplantation and low bone turnover and mineralization are the most common findings (411). More specifically, data obtained through bone biopsy have shown decreased bone formation and cortical thickness 6 months after transplantation, and these findings seem to be maintained even 10 years after transplantation (411). Sun et al, in a recent single-center cohort study (412), reported an increase of low bone turnover 1 year after transplantation by demonstrating a significant decrease of bone turnover markers (osteocalcin, NTX, and CTX levels). They concluded that the prevalence of femoral neck and lumbar spine bone loss increased, and they proved a positive correlation of this loss with higher levels of PTH and bone turnover markers both before and after kidney transplantation. In a prospective single-center cohort study, Ferreira et al analyzed the evolution of biochemical and histological parameters pre- and 1 year after renal transplantation in 69 patients (414). On the contrary to what was observed by Sun et al (412), they reported no difference in bone mineralization and volume. Regarding biochemical aspects, sclerostin and FGF23 serum levels decreased in all patients (median reduction of 62% and 91.1%, respectively); PTH, BALP, and a-Klotho levels decreased in 89.8%, 68.1%, and 34.8% of patients, respectively. Regarding histological findings, researchers observed lower cortical bone porosity and significantly decreased cortical thickness; the number of patients with high-remodeling bone disease dramatically decreased, whereas the number of patients with low-remodeling bone disease significantly increased, 1 year after transplantation (20 vs 7 patients and 15 vs 31 patients, respectively).

Transplant recipients have a 4-fold increased risk of fractures than the general population (273). Moreover, kidney transplant recipients face an increased fracture risk (about 30% increase) during the first 3 years after transplantation compared to that of patients undergoing dialysis; up to one quarter of them may suffer a fracture in the first 5 years following kidney transplantation (411). It seems that peripheral sites are affected more than central skeleton (411). In a recent meta-analysis of 28 studies and a total of 310 530 kidney transplant recipients, researchers concluded that the incidence of fracture was 10% (415). As results of earlier research suggest (416, 417), the higher incidence of posttransplant fracture was associated with the administration of steroid and immunosuppressive drugs, the recipient's age, female sex, pretransplant diabetes mellitus, pretransplant fracture, longer dialysis time, acute rejection, and deceased donator. In this setting, early steroid withdrawal (418) and vitamin D supplementation may halt bone loss but the administration of the new therapeutic options of bisphosphonates, denosumab, teriparatide, and cinacalcet must be very cautious and selective. PTX may improve BMD in symptomatic patients with persistent SHPT after transplantation (419).

## Hypoparathyroidism

HypoPTH is a rare disorder characterized by deficient production or biological activity of PTH that results in hypocalcemia and hyperphosphatemia. The prevalence of HypoPTH is estimated to be 37/100 000 habitants per year in the United States (420). HypoPTH may be an acquired or inherited condition, with postsurgical hypoparathyroidism being the most common form. Neck surgery is responsible for about 75% of HypoPTH cases (421). The next most frequent cause is immunity dysregulation, either affecting only parathyroid glands or multiple endocrine glands (autoimmune polyglandular syndromes) (422). Other causes include infiltrative diseases and rare genetic disorders (421, 423, 424). However, it is important to exclude magnesium disorders early in our differential diagnosis process, as both hypo- and hypermagnesemia can lead to functional HypoPTH (421). The diagnosis is based on the finding of low ionized or corrected for albumin calcium levels, accompanied by undetectable, low, or inappropriately normal PTH levels (425). Under normal circumstances, low calcium levels trigger the production and excretion of PTH from parathyroid glands, via activation of CaSRs. PTH deficiency inhibits mobilization of calcium from bone, reabsorption of calcium from kidneys, and absorption of calcium from gastrointestinal tract through 1,25-dihydroxyvitamin D synthesis. As a result, hypocalcemia is established, 1,25-dihydroxyvitamin D is decreased because PTH can't stimulate 1-a hydroxylase, phosphate levels rise because of the lack of phosphaturic action of PTH, and the fraction of filtered calcium at the glomerulus is inappropriately increased.

Clinical presentation of HypoPTH usually includes neuromuscular signs and symptoms resulting from hypocalcemia, ranging from mild perioral numbness, paresthesias in the extremities, muscle cramps, and QT interval prolongation, to severe manifestations of arrhythmias, heart failure, seizures, and laryngospasm (421, 423). However, some patients, especially those with genetic forms of HypoPTH, may be asymptomatic, thus they may be diagnosed due to routine biochemical screening or due to concomitant findings. Typical facial features, chronic candidiasis, or other autoimmune endocrinopathies may direct the diagnosis to DiGeorge or APECED (autoimmune polyendocrinopathy and candidiasis with ectodermal dystrophy syndrome), respectively. Absent or low levels of PTH result in reduced bone turnover and subsequent rise of BMD at both cortical and trabecular sites. However, bone disease derives from increased bone mineralization and altered skeletal microarchitecture (30). Whether these alterations relate to an increased fracture risk remains to be clarified.

Preventing signs and symptoms of hypocalcemia, maintaining serum calcium levels in the low normal range or slightly below normal, preventing hypercalciuria and hypercalcemia, keeping the calcium-phosphate product below 55 mg^2^/dL^2^, and preventing renal (nephrocalcinosis/nephrolithiasis) and other extraskeletal calcifications are the main objectives of treatment (426). Conventional therapy of HypoPTH includes not only the emergency management of acute and severe hypocalcemia with IV administration of calcium gluconate, but also the management of chronic hypocalcemia with calcium, calcitriol, as well as magnesium supplements, whenever required. The normalization of serum calcium levels through traditional treatment involving calcium and active vitamin D fails to fully restore normal calcium homeostasis physiology. Instead, it leads to hypercalciuria and hyperphosphatemia because it lacks renal calcium reabsorption and phosphaturia typically stimulated by PTH. Consequently, this standard approach heightens the risk of extraskeletal calcifications and long-term kidney complications. Moreover, the absence of PTH results in abnormal bone remodeling and contributes to reduced quality of life for many hypoparathyroidism patients despite adhering to conventional therapy. These challenges have prompted scientific exploration into alternative therapeutic avenues. Since the mid-1990s, synthetic PTH (1-34) has been proposed for the management of refractory HypoPTH, with promising results. rhPTH (1-84) has been approved for the treatment of adults with HypoPTH refractory to conventional therapy, with the exception of patients who have autosomal dominant hypocalcemia (425).

### Postsurgical HypoPTH and nonsurgical HypoPTH (NsHypoPTH)

HypoPTH most commonly occurs after irreversible damage or removal of parathyroid glands during neck surgery for thyroid goiter, head and neck cancer, or hyperparathyroidism. There are multiple definitions of postsurgical HypoPTH (PH) reported in literature (427). According to Asari et al (428), the term refers to a decrease of PTH with or without symptoms related to hypocalcemia, or to the neuromuscular symptoms related to postoperative calcium levels between 1.9 and 2.1 mmol/L. However, it is best defined as an undetectable or inappropriately low postoperative PTH combined with hypocalcemia, with or without hypocalcemic symptoms (429). It is classified into two subtypes, depending on the duration of symptoms: the transient HypoPTH persists for a period of ≤12 months, appears within 48 hours of surgery while permanent HypoPTH persists for >12 months, accounting for the minority of cases (430). If PTH values are >10 pg/mL 12-24 hours postoperatively, the development of permanent HypoPTH is very unlikely (430). Although it usually occurs early postoperatively, cases of HypoPTH presentation years after neck surgery have been described (431, 432). Hypocalcemia is the most frequent complication after thyroidectomy, and it is estimated that transient HypoPTH occurs in 25.4-83% of patients worldwide after neck surgery, whereas permanent HypoPTH occurs in 0.12-4.6% of patients (420). The profound variation in incidence highlights the need for increase of awareness of the condition (427). Extensive or repeated neck operations, Graves’ disease history, total thyroidectomy, bilateral neck exploration for PHPT, failure to identify >2 parathyroid glands during surgery or presence of parathyroid glands in the specimens, postoperative complications, younger age, vitamin D deficiency, a lower preoperative and a lower postoperative corrected calcium, as well as a larger decline in postoperative calcium and PTH are well recognized risk factors for PH (421, 433). The milestone of preventative methods is intraoperative localization and mobilization of parathyroid glands, while autotransplantation of the removed gland may significantly decrease the incidence of PH (434).

Nonsurgical HypoPTH (NsHypoPTH) includes all the other nonoperative causes of HypoPTH. In clinical practice, because of limited access to genetic screening, patients with NsHypoPTH are considered as a heterogenous group, including acquired (autoimmune, after radiation, infiltration) and genetic disorders (syndromic, hereditary isolated HypoPTH). Idiopathic HypoPTH refers to HypoPTH with no identifiable etiology.

### Bone Involvement in HypoPTH

Direct histomorphometric analysis of bone by transiliac bone biopsy offers more data regarding bone turnover in HypoPTH patients. The first histomorphometric study (435), involving 12 HypoPTH patients treated with varying doses of vitamin D, suggested that PTH deficiency is associated with significant reduction in bone remodeling activity, as reflected by extended quiescent period, decreased activation frequency, and decreased resorption and formation rate compared to controls. However, the amount and microarchitecture of the cancellous bone were normal. Rubin et al, in a larger 2-dimensional histomorphometric study (436), confirmed that HypoPTH is characterized by profound decrease of bone resorption rate at all sites (cancellous, endocortical, and intracortical). In addition, they reported significantly reduced osteoid width, osteoid surface, and mineral apposition at all sites. Structural analysis revealed greater cancellous bone volume and trabecular width in HypoPTH patients, whereas there was a nonsignificant tendency of HypoPTH patients to have increased cortical width. The same research group (437) extended their observation to a 3-dimensional histomorphometric analysis using micro-CT. They reported that cancellous bone architecture is markedly altered in HypoPTH: bone surface density, cancellous bone volume, trabecular thickness and number, and connectivity density are significantly greater in HypoPTH subjects, implying a “plate-like” trabecular structure. Low bone turnover in HypoPTH is correlated with reduced bone formation (procollagen type 1 amino-terminal propeptide, osteocalcin, bone-specific ALP) and resorption (C-telopeptide, TRAP5b) markers (30, 422).

Reduced bone remodeling resulting in positive bone balance explains the increased BMD and abnormal microstructure at all sites. One of the first studies to address this used single photon absorptiometry to assess bone mass in 19 PH female patients and compare them with controls (438). It concluded that, several years after surgery, bone mass was 10% to 32% greater in PH patients than in age- and sex-matched controls. The Canadian Multicentre Osteoporosis Study (439) compared 60 HypoPTH patients treated with conventional therapy with controls. Areal BMD obtained by DXA was found to be above average at the lumbar spine, hip, distal 1/3 of radius, and ultradistal radius in HypoPTH women of any age, at lumbar spine, hip, and femoral neck in young men, and at lumbar spine and ultradistal radius in men ≥ 50 years old. The increase in BMD seems to be most notable at the lumbar spine than at the femoral neck (440), and possibly is correlated to the suppressed bone remodeling, as supported by the significantly lower levels of bone specific ALP in HypoPTH patients (441). Greater insight into bone microarchitecture in HypoPTH has been obtained by HRpQCT; vBMD findings are in line with previous aBMD results. Both cortical and trabecular vBMD obtained by HRpQCT was reported to be increased in HypoPTH patients compared to controls (439, 442). Cortical porosity was diminished at both sites in HypoPTH women, whereas ultimate stress and failure load were similar in both groups. However, more recent publications have revealed different findings (426). HypoPTH patients had higher overall volumetric densities, but their cortical densities were lower than those of historical controls; they showed increases in cortical area, thickness, and porosity. Trabeculae were less sparse and more abundant in HypoPTH compared to controls. The different porosity measurement techniques used in each study may be responsible for the discrepancies in cortical density and porosity. Cipriani et al (137) examined TBS in 52 HypoPTH subjects, compared to 27 patients with PHPT, following correction of PTH deficiency or excess, respectively. At baseline, TBS was normal in patients with HypoPTH and significantly greater than in PHPT, but only subjects with HypoPTH on rhPTH(1-84) improved their microarchitecture texture as evaluated by TBS.

Fracture data in HypoPTH population are sparse and diverse. Dense hypermature bone suggests that HypoPTH patients are potentially more prone to fracture than euparathyroid individuals. Starr et al (443) used microindentation to measure cortical material bone strength in 17 HypoPTH patients. The results showed lower bone material strength index in HypoPTH patients compared to matched controls, implying that PTH deficiency may lead to bone matrix abnormalities that may reduce the ability of bone to resist microfractures. Large registry studies in Denmark (444) showed a significantly increased risk of fractures at the upper extremities, whereas reported no difference in overall fracture rate between NsHypoPTH patients and controls. A small cohort (445) showed an increase in morphometric vertebral fractures in postmenopausal women with HypoPTH, despite normal or increased BMD. Recently, more studies have generated more data toward an elevated vertebral fracture risk in HypoPTH. A large study assessing 50 postmenopausal HypoPTH patients by vertebral fracture assessment (446), reported a 16% prevalence of vertebral fracture in HypoPTH women in comparison to 7.5% in control subjects. Saha et al (447). observed an even higher vertebral fracture prevalence (31%) among 152 HypoPTH patients, although TBS was low only in one fourth of them. On the other hand, a large-population retrospective study (448) reported a reduced risk of upper extremities and proximal humerus fractures in PH patients compared with controls, although fractures at other specific skeletal sites did not differ between groups. In addition, a recent Korean retrospective cohort study analyzed data from 115 821 thyroid cancer patients who underwent total thyroidectomy (449). After controlling for covariates, postoperatively, the HypoPTH group had a significantly lower incidence of any fractures than the intact parathyroid function group (HR 0.83; 95% CI). This difference was attributed only to the significantly lower risk of vertebral fractures between the 2 groups.

Chan et al (441) demonstrated that patients with idiopathic HypoPTH experience a similar increase in BMD at lumbar spine and proximal femur obtained by DXA, and similar ALP decrease compared to patients with PH. As mentioned, data regarding fracture risk in HypoPTH patients are ambiguous (445, 448, 450, 451). A recent systematic review and meta-analysis (452) concluded that the increased risk of vertebral fractures concerns only patients with NsHypoPTH. More specifically, they are at almost 2-fold increased risk of a vertebral skeletal event. Patients with PH are not at an increased or decreased risk of any fractures than controls. In conclusion, these conflicting data may remain unresolved, since HypoPTH is an uncommon disorder and bone fracture a rare event.

### Effect of Treatment on Bone Disease in HypoPTH

#### Conventional treatment

The conventional therapy of HypoPTH consists of calcium and active vitamin D supplementation. The usually required amount of calcium supplements (mainly carbonate or citrate) varies from 1 to 3 grams per day, divided into multiple doses. Regarding vitamin D supplementation, calcitriol is the most widely used form of active vitamin D, usually required in a dose of 0.25 to 2 μg/day. Native vitamin D, cholecalciferol, may also be administered in doses up to 2000 IU/day, in order to reach adequate serum levels of 25-hydroxyvitamin D (434). In addition, treatment with thiazide diuretics combined with a low-sodium diet may contribute to hypercalciuria control. Phosphate binders and/or a low-phosphate diet may be advised to control persistent hyperphosphatemia (453). However, in some cases, conventional treatment is inadequate to control the biochemical parameters of the disorder, even with high doses of calcium and calcitriol supplementation. In addition, there is always the concern of hypercalciuria and the related renal and ectopic calcifications with prolonged administration of the supplements in large doses. These disadvantages, along with the limited ability of the conventional treatment to improve the quality of life (454, 455) and reverse bone abnormalities in HypoPTH (436, 456), brought out the need for a new more physiological treatment.

#### PTH (1-84)

Intact PTH became a focus of therapeutic interest as a replacement hormone therapy in HypoPTH because the full-length peptide is exactly what is missing. Several investigative groups have studied rhPTH (1− 84) over the past years. Rubin et al (457) demonstrated that subcutaneous administration of 100 μg intact PTH every other day for 2 years significantly reduced the need in calcium and vitamin D supplements in HypoPTH patients, without altering serum and urinary calcium levels. In addition, BMD increased at the lumbar spine, whereas it decreased at the distal 1/3 of radius and remained unchanged at the femoral neck. In a double-blind study with a 24-week duration (458), daily treatment with 100 µg PTH (1-84) reduced the daily need of calcium and active vitamin D supplementation by 75% and 73%, respectively, although hypercalcemia occurred frequently during the period of down-titration of the supplements. It seems that PTH (1-84) treatment maintains its efficacy and safety in reducing the need for supplements and increasing lumbar spine BMD for up to 4 years (459). In addition, it increases bone turnover, as reflected by the elevation of both formation and resorption markers (458). The improvement of bone turnover was later confirmed in percutaneous iliac crest bone biopsies by histomorphometric and 3-dimensional assessment with micro-CT (460, 461). Researchers found that PTH (1-84) treatment led to reduction of trabecular width and trabecular bone tissue density, whereas increased trabecular number, cortical porosity, connectivity density, mineralizing surface, and biochemical markers of bone turnover. Of interest, van Dijk Christiansen et al recently reported that rhPTH (1-84) replacement therapy for 6 months in patients with chronic HypoPTH although did not affect the overall cortical microstructure (cortical porosity, thickness, pore density, and mean pore diameter), it promoted the intracortical remodeling, and in specific, its transition from erosion to formation (462). The more interesting was that this effect persisted for 30 months and was reversible when treatment was withdrawn.

The pivotal study that led to the approval of recombinant human PTH (1-84) [rhPTH (1-84)] by the US Food and Drug Administration and European Medicines Agency in 2015 for treatment of patients with refractory HypoPTH (463) was the double-blind, placebo-controlled, randomized, phase 3 REPLACE trial (464). A total of 134 HypoPTH patients were enrolled in the 24-week study, 90 of whom received rhPTH (1-84) daily, in a dose that could be titrated up from 50 μg to 75 μg and then 100 μg. Fifty-three percent of patients in the treatment group achieved a ≥50% reduction from baseline in their daily dose of calcium and active vitamin D supplements, while maintaining serum calcium levels within a desirable range. The adverse events of hypocalcemia, muscle spasm, numbness, and headache were similar to placebo. This study was followed by 2 open-label extension trials (465, 466), which confirmed the safety and efficacy of the drug for up to 5 years, in reducing the daily doses of oral calcium and vitamin D as well as decreasing phosphate levels, calcium-phosphorus product levels, and 24-hour urinary calcium levels. In addition, Rubin et al investigated the long-term effects of rhPTH (1-84) treatment for up to 10 years and documented a significant and sustained reversal of baseline defects in bone parameters of HypoPTH subjects. By performing transiliac crest bone biopsies, they observed significant increases in cancellous bone volume, trabecular number, intra-trabecular tunneling, bone formation and mineralization and cortical porosity, to levels that even exceed those of healthy controls, after an average of 8 years of treatment in 13 HypoPTH patients (467). The same research team later conducted a study evaluating the effects of PTH (1-84) therapy on skeletal microarchitecture in HypoPTH patients (426). The majority of the patients treated were administered rhPTH (1-84) at a dose of 100 μg every other day for 4 years. Using DXA, the authors demonstrated increases in BMD at sites of trabecular bone (spine and femoral neck) and declines at sites of cortical bone (total hip and 1/3 radius). Moreover, they confirmed these results using HRpQCT, showing that vBMD declined in cortical but not in trabecular sites. The differences between DXA/HRpQCT results and those from bone biopsy reflect that iliac crest probably do not provide a reliable representation of all the other skeletal sites. In addition, the deterioration of cortical BMD was associated with significant decrease of bone stiffness at the tibia and failure load at the radius and tibia. In their latest published study, 27 HypoPTH patients were treated with rhPTH (1-84) for up to 12 years (468). The prolonged use of PTH (1-84) was associated with reduction in the need for calcium and calcitriol supplementation and reduction of urinary calcium excretion and nephrolithiasis. The reduction in pill burden is very important for some patients’ quality of life, but also probably attribute to the preservation of renal function and reduction of nephrolithiasis. PTH (1-84) was also associated with increase of aBMD at lumbar spine and decrease of aBMD at 1/3 radius, decrease of vBMD at tibia (maximum of −20%, 5 years after treatment) and radius, and increase of cortical porosity at both tibia and radius. Failure load decreased but still remained higher than that of normal controls at year 12.

Recently, a meta-analysis of data from the largest collection to date of HypoPTH patients (469), highlighted the safety and efficacy of PTH (1-84) in normalizing serum phosphate and urinary calcium excretion, decreasing calcium-phosphate product, minimizing the doses of conventional therapy and improving quality of life. Despite the heterogeneity between existing studies, PTH (1-84) increased bone turnover markers and bone remodeling, accompanied by an increase in lumbar spine BMD. It remains unclear whether the biochemical and densitometric outcomes will be related to fracture benefits. Although the use of PTH (1-34) for osteoporosis is approved for a maximum of 24 months’ duration because of the “black-box” warning because of the history of rat osteosarcoma, in HypoPTH there hasn't been defined a maximum duration of therapy for PTH 1-84 (425).

#### PTH (1-34)

Teriparatide, a rhPTH (1-34), is an approved treatment for postmenopausal osteoporosis, and it is currently being studied as a possible off-label treatment for postoperative HypoPTH. The investigation team of Winer et al first established the basis for the experimental use of PTH (1-34) in HypoPTH. They showed that treatment with teriparatide in a once-daily regimen in children and adults with HypoPTH was beneficial in improving biochemical indices and bone turnover and that its efficacy was even greater in a twice-daily dosing regimen (470-472). Of note, long-term administration of the twice-daily regimen was proven to be well-tolerated and superior to conventional treatment in maintaining normal calcium levels and skeletal development (473, 474). Forty-two patients with PH and refractory hypocalcemia were enrolled in a prospective open-label multicenter study to assess the efficacy of a twice-daily regimen of PTH (1-34) (475, 476). It was reported that treatment with 20 μg PTH (1-34) subcutaneously twice-daily rapidly raised serum calcium levels and maintained them within the normal range throughout therapy, 2 years later; the need for calcium and calcitriol supplements reduced; phosphate levels significantly dropped, whereas calcium-phosphorus product increased and stayed consistent for the course of the trial. Concurrently, PTH (1-34) therapy led to a significant improvement in the patients’ quality of life. Nonetheless, the effects and dose-response of treatment may be altered according to the specific cause of HypoPTH (477). Moreover, Winer et al introduced a pump delivery system of continuous administration of teriparatide, that is closer to a physiological method of replacement. It requires a smaller daily dose compared to multiple-daily regimen to exhibit its actions, makes it easier for the patient to comply with therapy and was able to normalize 24-hour urinary calcium excretion (478, 479). Concerning the effect on bone structure and turnover, Gafni et al (480), in a small trial with 5 hypoparathyroid patients, demonstrated using histomorphometry that daily teriparatide treatment dramatically increased trabecular, endocortical, and intracortical remodeling, leading to increased cancellous bone volume, trabecular number, intra-trabecular tunneling, and cortical porosity, and decreased trabecular separation. BMD changes varied depending on site, with total body and radial BMD being decreased, whereas total hip *Z*-scores being increased and spine and femoral neck *Z*-scores remaining unchanged. However, there are no data regarding the effect of teriparatide on bone strength and fracture risk.

#### PTH (1-84) vs PTH (1-34)

There hasn't been a published head-to-head analysis of the pharmacodynamics of the 2 PTH variants. Based on the meta-analysis by Puliani et al (469), treatment with PTH (1-34) had no significant differences compared to PTH (1-84), except for the nonefficacy of teriparatide in reducing calcium-phosphorus product levels. In a recent systematic review and meta-analysis of 7 RCTs, authors reported that in opposition to PTH (1-34), PTH (1-84) therapy leads to down-titration or cessation of calcium and active vitamin D supplements (481). In general, PTH therapy was associated with reductions in serum phosphate, but increased incidence of hypercalcemia.

Recently, Charoenngam et al published the first real-world data regarding the clinical and biochemical results of patients with HypoPTH who underwent a shift in treatment from conventional therapy to subcutaneous injections of rhPTH (1-84) or rhPTH (1-34), and subsequently to continuous pump therapy administration of rhPTH (1-84) or rhPTH (1-34) (482). The predicted total daily doses resulting from the initial basal rates of delivery would be roughly 80% of the prior rhPTH (1-84)/rhPTH (1-34) dose needs of single/multiple daily injections. Based on the patient's symptoms, blood calcium concentrations, and 24-hour urine calcium levels, the infusion rates were titrated on each outpatient visit. Twelve patients were included in total (1 with idiopathic HypoPTH and 11 with PH). Throughout the observation time (0.2-6.7 years), researchers recorded a reduction in serum phosphate concentrations and the return of urinary calcium excretion and serum calcium concentrations to normal. The frequency of hypocalcemia in response to multiple daily PTH injections and PTH pump therapy was lower than the frequency of hypocalcemia in response to single daily PTH injections (not statistically significant difference).

#### TransCon PTH

Although PTH (1-84) therapy has obtained a significant reduction in the need for calcium and active vitamin D supplementation, once-daily administration is not effective in reducing the incidence of hypercalcemia, hypocalcemia, hypercalciuria, and hyperphosphatemia. This may be attributed to the short half-life (approximately 3 hours) of subcutaneously administered PTH (1-84) (483). As mentioned, the effect of PTH (1-34) is increased when administered twice daily compared with a once-daily regimen, and continuous subcutaneous infusion was proven to be superior compared with twice-daily regimen on all endpoints (471, 472, 478). Therefore, it seems that regimens that mimic endogenous PTH secretion have better results regarding normalization of serum calcium, urine calcium and bone remodeling. To achieve a prolonged half-life of PTH in a way that resembles the regimen of continuous sc. administration, TransCon PTH was developed. TransCon PTH is an inactive prodrug of PTH (1-34), that has been manufactured by binding the TransCon linker to the mPEG carrier that prevents PTH from binding to its receptor and from being cleaved through kidneys and enzymatic process. Physiologic temperature and pH control the enzyme-independent cleavage of TransCon linker and then active PTH is released in a controlled manner, achieving prolonged half-life of approximately 2.5 days (483). Holten-Andersen et al when administered TransCon PTH once daily to intact rats and monkeys and to thyroparathyroidectomized rats, reported maintained and steady systemic concentration of PTH within the normal range and sustainable, dose-dependent increases in serum calcium levels, and concomitant decreases in serum phosphate levels (483). During its successful once-daily administration in healthy adults in the phase 1 clinical trial (484), TransCon PTH was proven to be well tolerated, without any serious related adverse events. In addition, it was shown to have a prolonged half-life (approximately 60 hours) and a dose-dependent effect on serum calcium and phosphate and urine excretion of calcium and phosphate. These results were confirmed in the phase 2 PaTH Forward trial that included 58 patients with HypoPTH (485). As early as week 4, the majority of treated patients experienced independence from oral vitamin D and calcium supplementation and, by week 26, they experienced normalization of serum calcium and calcium renal reabsorption. Regarding the impact of TransCon PTH on bone, it has been demonstrated that P1NP peaked 26 weeks and CTX peaked 12 weeks after initiation of PTH therapy, and thereafter trended downward through week 110 (despite the increase of daily TransCon PTH dose). In general, TransCon PTH is able to increase both P1NP and CTX bone markers, reflecting the potential restoration of the physiologic activity of PTH. The results of the phase 3 clinical trial confirmed its safety profile and supported the generalization of the results to a broader population of HypoPTH patients (61 adult patients) (486). Individualized titration of the dose of TransCon (ranging from 6 to 60 μg/day) was associated with marked and maintained improvement of serum calcium levels within the normal range, normalization of urine calcium excretion, serum phosphate, and calcium-phosphate product, and with independence from active vitamin D and therapeutic doses of calcium supplementation. However, more data from ongoing open-label extensions are needed to evaluate its long-term effects, including the effects on bone parameters and fracture risk.

## Pseudohypoparathyroidism

Pseudohypoparathyroidism with its types PHP1A, PHP1B, and PHP1C, was first described by Albright as a disorder caused by resistance of target tissues to PTH and characterized by hypocalcemia and hyperphosphatemia (487). Resistance to other hormones that act through G protein-coupled receptors could also be observed (such as TSH and GnRH), which may result in relevant clinical phenotypes.

Patients with PHP1A and PHP1C may also present with a spectrum of clinical features, among them short stature from premature closure of growth plates, ectopic ossifications, a stocky build, and round face. This heterogenous phenotype is described as Albright hereditary osteodystrophy (AHO). There are also patients that display AHO features in the absence of hormone resistance; depending on the number and the severity of AHO clinical features, the disorder can be classified as pseudopseudohypoparathyroidism (PPHP), progressive osseous heteroplasia, or osteoma cutis, which are recently encompassed by the term “disorders related to pseudohypoparathyroidism” (487).

PHP is inherited in an autosomal dominant manner, though de novo, sporadic, as well as epigenetic alterations within or upstream of genes involved in the PTH/PTHrP signaling (ie, GNAS, PRKAR1A, PDE4D, or PDE3A) also constitute common pathogenetic mechanisms.

Clinical presentation varies considerably among different sexes, and the genotype-phenotype correlation is poor even among the same sex, attributed to, among other causes, the inheritance of the responsive gene through parental imprinting. Moreover, the clinical and biochemical characteristics may remain unnoticed during infancy and early childhood and develop later in childhood, leading to delayed diagnosis. Interestingly, in PHP1A, PTH resistance evolves over life (up to 22 years old) (488).

### Bone Involvement in Rare Forms of PseudoHypoparathyroidism

Brachydactyly type E is the most common bone deformity among patients with PHP1A (70%-80%), whereas in PHP1B and PPHP, it presents only in 15% to 33% and <30% of patients, respectively (487). It is manifested as shortened metacarpal and metatarsal bones, with the fifth, fourth, and third metacarpals and the first and fourth distal phalanges being the most affected bones of the hand. Short stature is also a common skeletal manifestation of PHP. It is noteworthy that different distinct patterns of skeletal growth have been recognized in different PHP subtypes. Recently, Hanna et al, collecting data from 306 patients with PHP1A/PPHP and 220 patients with PHP1B, reported that 64% of PHP1A and 59% of PPHP adult patients had short stature, albeit some of them had normal height during adulthood (489). Patients with PHP1B had normal adult height. Moreover, a shortened or absent pubertal growth spurt remains a common feature across all PHP variants, postulating a crucial role of the impaired Gsa expression in stem cell-like chondrocytes, which might hinder the increase in growth velocity during puberty. Apart from brachydactyly and short stature, various other bone deformities such as Madelung deformity, spinal stenosis, craniofacial abnormalities (class III malocclusion with maxillary retrusion), acro-osteolysis, metaphyseal enchondromas, craniosynostosis, cortical irregularity of long bones (such as short humerus and curved radius), and ectopic ossifications have been described in various types of PHP and related disorders (490). Ectopic ossifications due to de novo formation of ectopic bone in dermis and subcutaneously are commonly found in patients with progressive osseous heteroplasia, PPHP/AHO, and PHP1A, in 100%, 80% to 100%, and 30% to 70%, respectively, whereas these are very uncommon among PHP1B patients (491). Bone deformities might not be visible in early life and develop over time. Thus, the performance of clinical and X-ray examination early in life is necessary to suspect and finally put the diagnosis of PHP. Several mechanisms have been postulated to explain the abovementioned bone manifestations in PHP. Brachydactyly and short stature have been attributed to impaired PTH/PTHrP signaling pathway in chondrocytes because of the monoallelic Gsα gene expression, leading to accelerated closure of growth plates. A similar defect of PTH/PTHrP signaling in mesenchymal stem cells could promote ectopic ossification (492).

Because GNAS has a biallelic expression in bone tissues, it should be expected that bone tissue sensitivity to PTH could be affected in PHP patients. However, bone responsiveness to PTH is still controversial in different types of PHP1. The higher bone turnover markers in patients with PHP1 compared to NsHypoPTH point toward a partial response of bone to PTH in PHP1. Of interest, in patients with different types of PHP1, the changes in bone turnover were associated with changes in PTH (493). Duan et al also reported that the changes in bone turnover in PHP1 patients were associated with changes in PTH, meaning that correction of the high PTH concentration is necessary to improve abnormal bone metabolism secondary to high bone turnover (493).

There are no data on the prevalence of osteoporosis in patients with PHP. Studies regarding BMD in patients with PHP are sparse and have produced inconsistent results, showing similar, decreased, or increased bone mass compared with normal controls. Comparing BMD of 31 patients with PHP, 62 patients with NsHypoPTH, and age and gender-matched healthy controls, Underbjerg et al reported significantly lower vBMD (total and trabecular) at the spine and hip in PHP compared to NsHypoPTH, as assessed by QCT scan (450). Performing HR-pQCT scan analysis, authors also found that patients with PHP have lower trabecular area and trabecular number at the tibia, in comparison to both NsHypoPTH and healthy controls, indicating a preferential negative effect of the chronically elevated PTH to trabecular bone. The specific genetic cause of PHP was not verified in most of the PHP patients included in the study; thus, differences resulting from the genetic background could not be excluded. Looking specifically at 48 patients with PHP1B, Chu et al reported that BMD was lower in all measured sites (femoral neck, lumbar spine, and total hip) compared to patients with NsHypoPTH. However, *Z*-score was higher in lumbar spine when compared to total hip and femoral neck within the PHP1B group (494). In line with the previous results, Kanatani et al have reported that patients with PHP1B (n = 8) have significantly lower BMD *Z*-scores of the femoral neck than patients with NsHypoPTH (n = 5) and PH (n = 14) (495). *Osteitis fibrosa cystica* results from longstanding exposure of bone to increased PTH levels. Wang et al summarized 13 cases with PHP who developed *osteitis fibrosa cystica* (492). The vast majority of them were diagnosed with PHP1B and were women. Further research is warranted to understand the exact pathogenetic mechanism, especially in PHP1B. Higher BMD than normal has been described in patients with PHP1B, as well. The increased bone mass in such cases was attributed to the slightly increased PTH, which exerted rather anabolic than catabolic effects. Interestingly, histomorphometric analysis revealed increased bone formation in both endocortical and trabecular surface (496).

Chen et al, studying a case of PHP with the XLαs (extra-large isoform of alpha-subunit of Gsα) nonsense mutation, found that impaired osteoclast formation was mainly responsible for the increased bone mass due to decreased RANKL/OPG ratio and increased sclerostin and DKK-1 expression (497). PHP1A is also characterized by either normal or increased bone mass in spine, femoral neck, total hip and distal radius (498). The increased bone mass in patients with PHP was not accompanied by accordingly lower fracture risk.

Despite the limited number of studies and the small number of participants due to the rare nature of the disease, the available data point out that PTH is a negative predictor of BMD mainly in PHP1B patients, with an heterogenous response to its longstanding high levels in different sites of skeleton. Even fewer data have been reported concerning the risk of fractures in patients with PHP. There are only 2 epidemiological studies comparing PHP, NsHypoPTH, and matched controls, which conclude that there is not increased fracture risk for patients with PHP at any skeletal site (444, 499). Thus, because of the lack of evidence of increased fracture risk, it is not recommended to perform DXA scan routinely in PHP patients (487).

PHP treatment should be aimed at maintenance of serum PTH and calcium within normal levels in order to normalize bone metabolism. In specific, PTH levels should be kept in the upper normal limit to avoid either hypercalciuria and/or nephrocalcinosis because of excessive PTH suppression or osteoporosis from long-standing, insufficient inhibition of PTH. Calcium and vitamin D supplementation is very important, whereas active vitamin D analogs should be administered in cases of progressive increases of PTH, regardless of the presence of hypocalcemia. PTH, calcium, phosphorus, and vitamin D should be checked regularly (at least yearly in adults and every 6 months in children) (487).

In case osteoporosis is diagnosed, the patient should be followed and treated according to the standard of care. Additional causes that could be related to bone loss in such patients (ie, GH deficiency, hypogonadism, longstanding elevated PTH levels) should be treated as well. Since PTH resistance is the hallmark of PHP, administration of PTH or PTH analogs cannot be used. Of interest, patients with PHP1B have improved their BMD in both femoral neck and lumbar spine with intake of calcium and active analogs of vitamin D (494, 500). In case tertiary hyperparathyroidism is developed, surgical removal of the hyperplastic parathyroid is recommended (501). Oral cinacalcet can be administered when patients are unsuitable for surgery (502).

## Conclusions

Both the excess of PTH in PHPT and the lack of PTH in HypoPTH adversely affect bone quality, reflecting the significant role of PTH actions on bone metabolism and remodeling. Fortunately, early diagnosis nowadays has led to fewer cases of severe bone involvement. Bone disease is a hallmark in PHPT because continuous exposure of the skeleton to high PTH levels leads to increased bone remodeling, impaired mineralization, decreased BMD at both cortical and trabecular sites, and increased fracture risk. Although successful PTX remains the mainstay for treatment and is effective in normalizing biochemical indices with evidence of restoration of bone aspects, cinacalcet is a valuable therapeutic alternative for patients contraindicated for surgery or those with persistent or recurrent disease. However, it has no effect on BMD. On the other hand, it has been reported that antiresorptive agents (ie, bisphosphonates, denosumab) decrease bone resorption, restore BMD to an extent, but are less effective regarding calcium and PTH excess. Nevertheless, large-scale and long-term well-designed RCTs to investigate the short- and long-term benefits of PTX compared to nonsurgical treatments with regard to bone quantity and quality, as well as fracture risk are still needed.

CKD is an area of major clinical and scientific interest regarding bone metabolism. It includes a wide range of skeletal manifestations, ranging from hyperparathyroidism with high bone turnover to adynamic bone disease with low bone turnover or mixed disorder (uremic osteodystrophy). Cinacalcet has proved its efficacy in BMD improvement in CKD patients, while data suggest also a fracture risk reduction. Recently, novel oral calcimimetic agents (ie, etelcalcetide, evocalcet) provided promising effects on CKD-MBD. Moreover, small studies conducted in CKD patients with SHPT have assessed the safety and the efficacy of denosumab and romosozumab in increasing BMD and showed improvement of bone loss. However, large RCTs are required to better establish their efficacy in preservation of bone mass and reduction of fracture risk in CKD patients with SHPT. Furthermore, the conduct of larger well-designed trials comparing various interventions (ie, calcimimetics, antiosteoporotic agents, and PTX) will provide high-quality evidence for the development of guidelines for the treatment of advanced CKD-MBD.

On the other hand, HypoPTH, mainly represented by PH, leads to reduced bone turnover and elevated BMD. The impairment of bone microarchitecture arises from the accumulation of nonrenewable hypermature bone. However, data on bone strength and fracture risk remain controversial. Treatment with human recombinant PTH (1-84) is associated with bone remodeling restoration and BMD increase at trabecular sites. TransCon PTH demonstrates promising effects in terms of calcium normalization and restoration of BMD toward age- and sex-matched norms. Yet, a regimen that could replace PTH in a way that simulates tonic, circadian, and pulsatility patterns that characterize the normal physiological state is missing.

Data on the prevalence of osteoporosis and fracture risk in patients with PHP are sparse and have produced inconsistent results. PHP treatment is aimed at maintenance of serum PTH and calcium within normal levels to normalize bone metabolism.

More research is required to unfold the complex mechanisms and effects of chronic PTH deficiency or partial unresponsiveness on bone metabolism and microarchitecture, aiming to determine individualized fracture risk and a potent treatment for fracture prevention.

## Abbreviations

aBMD: areal bone mineral density
AHO: Albright hereditary osteodystrophy
ALP: alkaline phosphatase
BMD: bone mineral density
BSAP: bone-specific alkaline phosphatase
CaSR: calcium-sensing receptor
Ca^2+^: ionized calcium
CKD: chronic kidney disease
CT: computed tomography
CTX: C-telopeptide
CVD: cardiovascular disease
DKK-1: Dickkopf1
DXA: dual energy X-ray absorptiometry
FGF23: fibroblast growth factor 23
FHH: familial hypocalciuric hypercalcemia
GFR: glomerular filtration rate
HBS: hungry bone syndrome
HPHPT: hypercalcemic primary hyperparathyroidism
HPT-JT: hyperparathyroidism-jaw tumor syndrome
HR: hazard ratio
HRpQCT: high-resolution peripheral quantitative computed tomography
HypoPTH: hypoparathyroidism
iPTH: intact PTH
MBD: mineral and bone disorder
MCP-1: monocyte chemoattractant protein-1
MEN: multiple endocrine neoplasia
MTC: medullary thyroid cancer
NPHPT: normocalcemic primary hyperparathyroidism
NsHypoPTH: nonsurgical hypoparathyroidism
NTX: N-terminal telopeptide
P1NP: procollagen type 1 N-terminal propeptide
PFS: progression-free survival
PH: postsurgical hypoparathyroidism
PHPT: primary hyperparathyroidism
PKA: protein kinase A
PPHP: pseudopseudohypoparathyroidism
PTH1R: PTH/PTH-related protein receptor 1
PTHrP: PTH-related protein
PTX: parathyroidectomy
RCT: randomized controlled trial
rhPTH: recombinant human PTH
SERM: selective estrogen receptor modulator
SHPT: secondary hyperparathyroidism
THPT: tertiary hyperparathyroidism
TBS: Trabecular Bone Score
TRAP5b: tartrate-resistant acid phosphatase 5b
vBMD: volumetric bone mineral density
VDRA: vitamin D receptor activator

## References

[1] Tan RSG, Lee CHL, Dimke H, Todd Alexander R. The role of calcium-sensing receptor signaling in regulating transepithelial calcium transport. Exp Biol Med. 2021;246(22):2407‐2419. 10.1177/15353702211010415 PMC8606958 33926258

[2] Goltzman D . Physiology of parathyroid hormone. Endocrinol Metab Clin North Am. 2018;47(4):743‐758. 30390810 10.1016/j.ecl.2018.07.003

[3] Michels TC, Kelly KM. Parathyroid disorders. Am Fam Physician. 2013;88(4):249‐257. 23944728

[4] Fraser WD . Hyperparathyroidism. Lancet. 2009;374(9684):145‐158. 19595349 10.1016/S0140-6736(09)60507-9

[5] Tregear GW, Van Rietschoten J, Greene E, et al Bovine parathyroid hormone: minimum chain length of synthetic peptide required for biological activity. Endocrinology. 1973;93(6):1349‐1353. 4796246 10.1210/endo-93-6-1349

[6] Jüppner H, Abou-Samra AB, Freeman M, et al A G protein-linked receptor for parathyroid hormone and parathyroid hormone-related peptide. Science. 1991;254(5034):1024‐1026. 1658941 10.1126/science.1658941

[7] Martin TJ, Sims NA, Seeman E. Physiological and pharmacological roles of PTH and PTHrP in bone using their shared receptor, PTH1R. Endocr Rev. 2021;42(4):383‐406. 33564837 10.1210/endrev/bnab005

[8] Ferrandon S, Feinstein TN, Castro M, et al Sustained cyclic AMP production by parathyroid hormone receptor endocytosis. Nat Chem Biol. 2009;5(10):734‐742. 19701185 10.1038/nchembio.206 PMC3032084

[9] Atkins D, Hunt H, Ingleton PM, Martin TJ. Rat osteogenic sarcoma cells: isolation and effects of hormones on the production of cyclic AMP and cyclic GMP. Endocrinology. 1977;101(2):555‐561. 195798 10.1210/endo-101-2-555

[10] Rubin CS, Rangel-Aldao R, Sarkar D, Erlichman J, Fleischer N. Characterization and comparison of membrane-associated and cytosolic cAMP-dependent protein kinases. Physicochemical and immunological studies on bovine cerebral cortex protein kinases. J Biol Chem. 1979;254(10):3797‐3805. 220219

[11] Chen X, Dai JC, Orellana SA, Greenfield EM. Endogenous protein kinase inhibitor γ terminates immediate-early gene expression induced by cAMP-dependent protein kinase (PKA) signaling. J Biol Chem. 2005;280(4):2700‐2707. 15557275 10.1074/jbc.M412558200

[12] Hong AR, Lee JH, Kim JH, Kim SW, Shin CS. Effect of endogenous parathyroid hormone on bone geometry and skeletal microarchitecture. Calcif Tissue Int. 2019;104(4):382‐389. 30659307 10.1007/s00223-019-00517-0

[13] Kearns AE, Khosla S, Kostenuik PJ. Receptor activator of nuclear factor κB ligand and osteoprotegerin regulation of bone remodeling in health and disease. Endocr Rev. 2008;29(2):155‐192. 18057140 10.1210/er.2007-0014 PMC2528846

[14] Mcsheehy PMJ, Chambers TJ. Osteoblastic cells mediate osteoclastic responsiveness to parathyroid hormone. Endocrinology. 1986;118(2):824‐828. 3455914 10.1210/endo-118-2-824

[15] Xiong J, O’Brien CA. Osteocyte RANKL: new insights into the control of bone remodeling. J Bone Miner Res. 2012;27(3):499‐505. 22354849 10.1002/jbmr.1547 PMC3449092

[16] O’Brien CA, Nakashima T, Takayanagi H. Osteocyte control of osteoclastogenesis. Bone. 2013;54(2):258‐263. 22939943 10.1016/j.bone.2012.08.121 PMC3538915

[17] Khosla S . Minireview: the OPG/RANKL/RANK system. Endocrinology. 2001;142(12):5050‐5055. 11713196 10.1210/endo.142.12.8536

[18] Xiong J, Piemontese M, Thostenson JD, Weinstein RS, Manolagas SC, O’Brien CA. Osteocyte-derived RANKL is a critical mediator of the increased bone resorption caused by dietary calcium deficiency. Bone. 2014;66:146‐154. 24933342 10.1016/j.bone.2014.06.006 PMC4125539

[19] Xiong J, Onal M, Jilka RL, Weinstein RS, Manolagas SC, O’Brien CA. Matrix-embedded cells control osteoclast formation. Nat Med. 2011;17(10):1235‐1241. 21909103 10.1038/nm.2448 PMC3192296

[20] Nakashima T, Hayashi M, Fukunaga T, et al Evidence for osteocyte regulation of bone homeostasis through RANKL expression. Nat Med. 2011;17(10):1231‐1234. 21909105 10.1038/nm.2452

[21] Huang JC, Sakata T, Pfleger LL, et al PTH differentially regulates expression of RANKL and OPG. J Bone Miner Res. 2004;19(2):235‐244. 14969393 10.1359/JBMR.0301226

[22] Ma YL, Cain RL, Halladay DL, et al Catabolic effects of continuous human PTH (1–38) in vivo is associated with sustained stimulation of RANKL and inhibition of osteoprotegerin and gene-associated bone formation. Endocrinology. 2001;142(9):4047‐4054. 11517184 10.1210/endo.142.9.8356

[23] Lee SK, Lorenzo JA. Parathyroid hormone stimulates TRANCE and inhibits osteoprotegerin messenger ribonucleic acid expression in murine bone marrow cultures: correlation with osteoclast-like cell formation. Endocrinology. 1999;140(8):3552‐3561. 10433211 10.1210/endo.140.8.6887

[24] Szymczak J, Bohdanowicz-Pawlak A. Osteoprotegerin, RANKL, and bone turnover in primary hyperparathyroidism: the effect of parathyroidectomy and treatment with alendronate. Horm Metab Res. 2013;45(10):759‐764. 23888411 10.1055/s-0033-1349842

[25] Nakchbandi IA, Lang R, Kinder B, Insogna KL. The role of the receptor activator of nuclear factor-kappaB ligand/osteoprotegerin cytokine system in primary hyperparathyroidism. J Clin Endocrinol Metab. 2008;93(3):967‐973. 18073309 10.1210/jc.2007-1645 PMC2266956

[26] Stilgren LS, Rettmer E, Eriksen EF, Hegedüs L, Beck-Nielsen H, Abrahamsen B. Skeletal changes in osteoprotegerin and receptor activator of nuclear factor-κb ligand mRNA levels in primary hyperparathyroidism: effect of parathyroidectomy and association with bone metabolism. Bone. 2004;35(1):256‐265. 15207766 10.1016/j.bone.2004.03.012

[27] Li X, Qin L, Bergenstock M, Bevelock LM, Novack DV, Partridge NC. Parathyroid hormone stimulates osteoblastic expression of MCP-1 to recruit and increase the fusion of Pre/osteoclasts. J Biol Chem. 2007;282(45):33098‐33106. 17690108 10.1074/jbc.M611781200

[28] Patel H, Trooskin S, Shapses S, Sun W, Wang X. Serum monocyte chemokine protein-1 levels before and after parathyroidectomy in patients with primary hyperparathyroidism. Endocr Pract. 2014;20(11):1165‐1169. 24936562 10.4158/EP14104.OR

[29] Dempster DW, Parisien M, Silverberg SJ, et al On the mechanism of cancellous bone preservation in postmenopausal women with mild primary hyperparathyroidism ^1^. J Clin Endocrinol Metab. 1999;84(5):1562‐1566. 10323380 10.1210/jcem.84.5.5652

[30] Silva BC, Bilezikian JP. Skeletal abnormalities in hypoparathyroidism and in primary hyperparathyroidism. Rev Endocr Metab Disord. 2021;22(4):789‐802. 33200346 10.1007/s11154-020-09614-0

[31] Miao D, He B, Karaplis AC, Goltzman D. Parathyroid hormone is essential for normal fetal bone formation. J Clin Invest. 2002;109(9):1173‐1182. 11994406 10.1172/JCI14817 PMC150965

[32] Ren Y, Liu B, Feng Y, et al Endogenous PTH deficiency impairs fracture healing and impedes the fracture-healing efficacy of exogenous PTH(1–34). PLoS One. 2011;6(7):e23060. 21829585 10.1371/journal.pone.0023060 PMC3146536

[33] Wronski TJ, Yen CF, Qi H, Dann LM. Parathyroid hormone is more effective than estrogen or bisphosphonates for restoration of lost bone mass in ovariectomized rats. Endocrinology. 1993;132(2):823‐831. 8425497 10.1210/endo.132.2.8425497

[34] Shen V, Dempster DW, Birchman R, Xu R, Lindsay R. Loss of cancellous bone mass and connectivity in ovariectomized rats can be restored by combined treatment with parathyroid hormone and estradiol. J Clin Invest. 1993;91(6):2479‐2487. 8514860 10.1172/JCI116483 PMC443308

[35] Borba VZC, Mañas NCP. The use of PTH in the treatment of osteoporosis. Arq Bras Endocrinol Metabol. 2010;54(2):213‐219. 20485911 10.1590/s0004-27302010000200018

[36] Jilka RL . Molecular and cellular mechanisms of the anabolic effect of intermittent PTH. Bone. 2007;40(6):1434‐1446. 17517365 10.1016/j.bone.2007.03.017 PMC1995599

[37] Compston JE . Skeletal actions of intermittent parathyroid hormone: effects on bone remodelling and structure. Bone. 2007;40(6):1447‐1452. 17045858 10.1016/j.bone.2006.09.008

[38] Rubin MR, Bilezikian JP. Parathyroid hormone as an anabolic skeletal therapy. Drugs. 2005;65(17):2481‐2498. 16296873 10.2165/00003495-200565170-00005

[39] Wang YH, Liu Y, Rowe DW. Effects of transient PTH on early proliferation, apoptosis, and subsequent differentiation of osteoblast in primary osteoblast cultures. Am J Physiol Endocrinol Metab. 2007;292(2):E594‐E603. 17032929 10.1152/ajpendo.00216.2006

[40] Bellido T, Ali AA, Plotkin LI, et al Proteasomal degradation of runx2 shortens parathyroid hormone-induced anti-apoptotic signaling in osteoblasts. J Biol Chem. 2003;278(50):50259‐50272. 14523023 10.1074/jbc.M307444200

[41] Jilka RL, Weinstein RS, Bellido T, Roberson P, Parfitt AM, Manolagas SC. Increased bone formation by prevention of osteoblast apoptosis with parathyroid hormone. J Clin Invest. 1999;104(4):439‐446. 10449436 10.1172/JCI6610 PMC408524

[42] Hisa I, Inoue Y, Hendy GN, et al Parathyroid hormone-responsive Smad3-related factor, Tmem119, promotes osteoblast differentiation and interacts with the bone morphogenetic protein-Runx2 pathway. J Biol Chem. 2011;286(11):9787‐9796. 21239498 10.1074/jbc.M110.179127 PMC3058974

[43] Krishnan V, Moore TL, Ma YL, et al Parathyroid hormone bone anabolic action requires Cbfa1/Runx2-dependent signaling. Mol Endocrinol. 2003;17(3):423‐435. 12554794 10.1210/me.2002-0225

[44] Poole KES, Van Bezooijen RL, Loveridge N, et al Sclerostin is a delayed secreted product of osteocytes that inhibits bone formation. FASEB J. 2005;19(13):1842‐1844. 16123173 10.1096/fj.05-4221fje

[45] Bellido T, Ali AA, Gubrij I, et al Chronic elevation of parathyroid hormone in mice reduces expression of sclerostin by osteocytes: a novel mechanism for hormonal control of osteoblastogenesis. Endocrinology. 2005;146(11):4577‐4583. 16081646 10.1210/en.2005-0239

[46] Mirza FS, Padhi ID, Raisz LG, Lorenzo JA. Serum sclerostin levels negatively correlate with parathyroid hormone levels and free estrogen Index in postmenopausal women. J Clin Endocrinol Metab. 2010;95(4):1991‐1997. 20156921 10.1210/jc.2009-2283 PMC2853994

[47] van Lierop AH, Witteveen JE, Hamdy NAT, Papapoulos SE. Patients with primary hyperparathyroidism have lower circulating sclerostin levels than euparathyroid controls. Eur J Endocrinol. 2010;163(5):833‐837. 20817762 10.1530/EJE-10-0699

[48] Ardawi MSM, Al-Sibiany AM, Bakhsh TM, Rouzi AA, Qari MH. Decreased serum sclerostin levels in patients with primary hyperparathyroidism: a cross-sectional and a longitudinal study. Osteoporos Int. 2012;23(6):1789‐1797. 22041864 10.1007/s00198-011-1806-8

[49] Anastasilakis AD, Polyzos SA, Avramidis A, Toulis KA, Papatheodorou A, Terpos E. The effect of teriparatide on serum Dickkopf-1 levels in postmenopausal women with established osteoporosis. Clin Endocrinol (Oxf). 2010;72(6):752‐757. 19832854 10.1111/j.1365-2265.2009.03728.x

[50] Yao GQ, Wu JJ, Troiano N, Insogna K. Targeted overexpression of Dkk1 in osteoblasts reduces bone mass but does not impair the anabolic response to intermittent PTH treatment in mice. J Bone Miner Metab. 2011;29(2):141‐148. 20602130 10.1007/s00774-010-0202-3 PMC3457021

[51] Wang Y, Liu J. Severe bone disease caused by primary hyperparathyroidism: a case report and review of the literature. J Int Med Res. 2020;48(10):030006052096648. 10.1177/0300060520966484 PMC7645399 33100067

[52] Lundgren E, Hagström EG, Lundin J, et al Primary hyperparathyroidism revisited in menopausal women with Serum calcium in the upper normal range at population-based screening 8 years ago. World J Surg. 2002;26(8):931‐936. 12045863 10.1007/s00268-002-6621-0

[53] Mazeh H, Sippel RS, Chen H. The role of gender in primary hyperparathyroidism: same disease, different presentation. Ann Surg Oncol. 2012;19(9):2958‐2962. 22535262 10.1245/s10434-012-2378-3

[54] Cosman F . Estrogen protection against bone resorbing effects of parathyroid hormone infusion: assessment by use of biochemical markers. Ann Intern Med. 1993;118(5):337‐343. 8430979 10.7326/0003-4819-118-5-199303010-00003

[55] Rubin MR, Lee KH, McMahon DJ, Silverberg SJ. Raloxifene lowers Serum calcium and markers of bone turnover in postmenopausal women with primary hyperparathyroidism. J Clin Endocrinol Metab. 2003;88(3):1174‐1178. 12629102 10.1210/jc.2002-020667

[56] Selby PL, Peacock M. Ethinyl estradiol and norethindrone in the treatment of primary hyperparathyroidism in postmenopausal women. N Engl J Med. 1986;314(23):1481‐1485. 3754618 10.1056/NEJM198606053142304

[57] Zhu CY, Sturgeon C, Yeh MW. Diagnosis and management of primary hyperparathyroidism. JAMA. 2020;323(12):1186. 32031566 10.1001/jama.2020.0538

[58] Thakker RV . Genetics of parathyroid tumours. J Intern Med. 2016;280(6):574‐583. 27306766 10.1111/joim.12523

[59] Marini F, Giusti F, Cioppi F, et al Bone and mineral metabolism phenotypes in MEN1-related and sporadic primary hyperparathyroidism, before and after parathyroidectomy. Cells. 2021;10(8):1895. 34440663 10.3390/cells10081895 PMC8391385

[60] Arshad MF, McAllister J, Merchant A, et al Urinary calcium indices in primary hyperparathyroidism (PHPT) and familial hypocalciuric hypercalcaemia (FHH): which test performs best? Postgrad Med J. 2021;97(1151):577‐582. 32892159 10.1136/postgradmedj-2020-137718

[61] Bilezikian JP, Khan AA, Silverberg SJ, et al Evaluation and management of primary hyperparathyroidism: summary statement and guidelines from the fifth international workshop. J Bone Miner Res. 2022;37(11):2293‐2314. 36245251 10.1002/jbmr.4677

[62] Cormier C, Koumakis E. Bone and primary hyperparathyroidism. Joint Bone Spine. 2022;89(1):105129. 33484857 10.1016/j.jbspin.2021.105129

[63] Gasser RW . Clinical aspects of primary hyperparathyroidism: clinical manifestations, diagnosis, and therapy. Wien Med Wochenschr. 2013;163(17-18):397‐402. 23990260 10.1007/s10354-013-0235-z

[64] Wilhelm SM, Wang TS, Ruan DT, et al The American Association of Endocrine Surgeons guidelines for definitive management of primary hyperparathyroidism. JAMA Surg. 2016;151(10):959. 27532368 10.1001/jamasurg.2016.2310

[65] Van den Bruel A, Bijnens J, Van Haecke H, et al Preoperative imaging for hyperparathyroidism often takes upper parathyroid adenomas for lower adenomas. Sci Rep. 2023;13(1):7568. 37160895 10.1038/s41598-023-32707-0 PMC10169799

[66] Christiansen P, Steiniche T, Brixen K, et al Primary hyperparathyroidism: biochemical markers and bone mineral density at multiple skeletal sites in danish patients. Bone. 1997;21(1):93‐99. 9213014 10.1016/s8756-3282(97)00078-1

[67] Steiniche T, Christiansen P, Vesterby A, et al Primary hyperparathyroidism: bone structure, balance, and remodeling before and 3 years after surgical treatment. Bone. 2000;26(5):535‐543. 10773596 10.1016/S8756-3282(00)00260-X

[68] Valdemarsson S, Lindergård B, Tibblin S, Bergenfelz A. Increased biochemical markers of bone formation and resorption in primary hyperparathyroidism with special reference to patients with mild disease. J Intern Med. 1998;243(2):115‐122. 9566639 10.1046/j.1365-2796.1998.00241.x

[69] Vu TDT, Wang XF, Wang Q, et al New insights into the effects of primary hyperparathyroidism on the cortical and trabecular compartments of bone. Bone. 2013;55(1):57‐63. 23541782 10.1016/j.bone.2013.03.009 PMC4308951

[70] Charopoulos I, Tournis S, Trovas G, et al Effect of primary hyperparathyroidism on volumetric bone mineral density and bone geometry assessed by peripheral quantitative computed tomography in postmenopausal women. J Clin Endocrinol Metab. 2006;91(5):1748‐1753. 16492695 10.1210/jc.2005-2102

[71] Silverberg SJ, Shane E, de la Cruz L, et al Skeletal disease in primary hyperparathyroidism. J Bone Miner Res. 2009;4(3):283‐291. 10.1002/jbmr.5650040302 2763869

[72] Romagnoli E, Cipriani C, Nofroni I, et al “Trabecular bone score” (TBS): an indirect measure of bone micro-architecture in postmenopausal patients with primary hyperparathyroidism. Bone. 2013;53(1):154‐159. 23228370 10.1016/j.bone.2012.11.041

[73] Silverberg SJ, Shane E, Jacobs TP, Siris E, Bilezikian JP. A 10-year prospective study of primary hyperparathyroidism with or without parathyroid surgery. N Engl J Med. 1999;341(17):1249‐1255. 10528034 10.1056/NEJM199910213411701

[74] Parisien M, Mellish RWE, Silverberg SJ, et al Maintenance of cancellous bone connectivity in primary hyperparathyroidism: trabecular strut analysis. J Bone Miner Res. 2009;7(8):913‐920. 10.1002/jbmr.5650070808 1442205

[75] Stein EM, Silva BC, Boutroy S, et al Primary hyperparathyroidism is associated with abnormal cortical and trabecular microstructure and reduced bone stiffness in postmenopausal women. J Bone Miner Res. 2013;28(5):1029‐1040. 23225022 10.1002/jbmr.1841 PMC3631282

[76] Schoeb M, Winter EM, Sleddering MA, et al Bone material strength Index as measured by impact microindentation is low in patients with primary hyperparathyroidism. J Clin Endocrinol Metab. 2021;106(7):e2527‐e2534. 33780545 10.1210/clinem/dgab207 PMC8266436

[77] Zanocco KA, Yeh MW. Primary hyperparathyroidism. Endocrinol Metab Clin North Am. 2017;46(1):87‐104. 28131138 10.1016/j.ecl.2016.09.012

[78] Bilezikian JP, Brandi ML, Eastell R, et al Guidelines for the management of asymptomatic primary hyperparathyroidism: summary statement from the Fourth International Workshop. J Clin Endocrinol Metab. 2014;99(10):3561‐3569. 25162665 10.1210/jc.2014-1413 PMC5393490

[79] Hansen S, Beck Jensen JE, Rasmussen L, Hauge EM, Brixen K. Effects on bone geometry, density, and microarchitecture in the distal radius but not the tibia in women with primary hyperparathyroidism: a case-control study using HR-pQCT. J Bone Miner Res. 2010;25(9):1941‐1947. 20499376 10.1002/jbmr.98

[80] Silva BC, Boutroy S, Zhang C, et al Trabecular bone score (TBS)—a novel method to evaluate bone microarchitectural texture in patients with primary hyperparathyroidism. J Clin Endocrinol Metab. 2013;98(5):1963‐1970. 23526463 10.1210/jc.2012-4255 PMC3644593

[81] Narayanan N, Palui R, Merugu C, et al The risk of fractures in primary hyperparathyroidism: a meta-analysis. JBMR Plus. 2021;5(4):e10482. 33869997 10.1002/jbm4.10482 PMC8046118

[82] Eller-Vainicher C, Filopanti M, Palmieri S, et al Bone quality, as measured by trabecular bone score, in patients with primary hyperparathyroidism. Eur J Endocrinol. 2013;169(2):155‐162. 23682095 10.1530/EJE-13-0305

[83] Tabacco G, Naciu AM, Messina C, et al DXA-Based Bone strain Index: a new tool to evaluate bone quality in primary hyperparathyroidism. J Clin Endocrinol Metab. 2021;106(8):2304‐2312. 33963754 10.1210/clinem/dgab317 PMC8599893

[84] Castellano E, Attanasio R, Boriano A, et al Sex difference in the clinical presentation of primary hyperparathyroidism: influence of menopausal Status. J Clin Endocrinol Metab. 2017;102(11):4148‐4152. 28938410 10.1210/jc.2017-01080

[85] De Lucia F, Minisola S, Romagnoli E, et al Effect of gender and geographic location on the expression of primary hyperparathyroidism. J Endocrinol Invest. 2013;36(2):123‐126. 22718266 10.3275/8455

[86] Khosla S, Melton LJ, Wermers RA, Crowson CS, O’Fallon WM, Riggs BL. Primary hyperparathyroidism and the risk of fracture: a population-based study. J Bone Miner Res. 1999;14(10):1700‐1707. 10491217 10.1359/jbmr.1999.14.10.1700

[87] Vestergaard P . Cohort study of risk of fracture before and after surgery for primary hyperparathyroidism. BMJ. 2000;321(7261):598‐602. 10977834 10.1136/bmj.321.7261.598 PMC27473

[88] Vestergaard P, Mosekilde L. Fractures in patients with primary hyperparathyroidism: nationwide follow-up study of 1201 patients. World J Surg. 2003;27(3):343‐349. 12607064 10.1007/s00268-002-6589-9

[89] Khosla S, Melton J. Fracture risk in primary hyperparathyroidism. J Bone Miner Res. 2002;17(Suppl 2):N103‐N107. 12412786

[90] Ejlsmark-Svensson H, Rolighed L, Harsløf T, Rejnmark L. Risk of fractures in primary hyperparathyroidism: a systematic review and meta-analysis. Osteoporos Int. 2021;32(6):1053‐1060. 33527175 10.1007/s00198-021-05822-9

[91] Vanitcharoenkul E, Singsampun N, Unnanuntana A, Sirinvaravong S. Osteitis *Fibrosa cystica* and pathological fractures—the classic but neglected skeletal manifestation of primary hyperparathyroidism: a case report. BMC Musculoskelet Disord. 2021;22(1):443. 33990191 10.1186/s12891-021-04326-1 PMC8122575

[92] Yang Q, Sun P, Li J, et al Skeletal lesions in primary hyperparathyroidism. Am J Med Sci. 2015;349(4):321‐327. 25798829 10.1097/MAJ.0000000000000441

[93] Bandeira F, Cusano NE, Silva BC, et al Bone disease in primary hyperparathyroidism. Arq Bras Endocrinol Metabol. 2014;58(5):553‐561. 25166047 10.1590/0004-2730000003381 PMC4315357

[94] Makras P, Anastasilakis AD. Bone disease in primary hyperparathyroidism. Metabolism. 2018;80:57‐65. 29051042 10.1016/j.metabol.2017.10.003

[95] Schini M, Jacques RM, Oakes E, Peel NFA, Walsh JS, Eastell R. Normocalcemic hyperparathyroidism: study of its prevalence and natural history. J Clin Endocrinol Metab. 2020;105(4):e1171‐e1186. 32072184 10.1210/clinem/dgaa084 PMC7069345

[96] Naciu AM, Tabacco G, Falcone S, et al Bone quality as measured by trabecular bone score in normocalcemic primary hyperparathyroidism. Endocr Pract. 2021;27(10):992‐997. 33962077 10.1016/j.eprac.2021.04.884

[97] Palermo A, Naciu AM, Tabacco G, et al Clinical, biochemical, and radiological profile of normocalcemic primary hyperparathyroidism. J Clin Endocrinol Metab. 2020;105(7):dgaa174. 32271382 10.1210/clinem/dgaa174

[98] Lowe H, McMahon DJ, Rubin MR, Bilezikian JP, Silverberg SJ. Normocalcemic primary hyperparathyroidism: further characterization of a new clinical phenotype. J Clin Endocrinol Metab. 2007;92(8):3001‐3005. 17536001 10.1210/jc.2006-2802

[99] Silverberg SJ, Bilezikian JP. “Incipient” primary hyperparathyroidism: a “forme fruste” of an old disease. J Clin Endocrinol Metab. 2003;88(11):5348‐5352. 14602772 10.1210/jc.2003-031014

[100] Maruani G, Hertig A, Paillard M, Houillier P. Normocalcemic primary hyperparathyroidism: evidence for a generalized target-tissue resistance to parathyroid hormone. J Clin Endocrinol Metab. 2003;88(10):4641‐4648. 14557434 10.1210/jc.2002-021404

[101] Tabacco G, Naciu AM, Messina C, et al DXA-based bone strain index in normocalcemic primary hyperparathyroidism. Osteoporos Int. 2023;34(5):999‐1003. 36640186 10.1007/s00198-023-06669-y

[102] Kontogeorgos G, Trimpou P, Laine CM, Oleröd G, Lindahl A, Landin-Wilhelmsen K. Normocalcaemic, vitamin D-sufficient hyperparathyroidism—high prevalence and low morbidity in the general population: a long-term follow-up study, the WHO MONICA project, Gothenburg, Sweden. Clin Endocrinol (Oxf). 2015;83(2):277‐284. 25988687 10.1111/cen.12819 PMC4744766

[103] Muñoz de Nova JL, Sampedro-Nuñez M, Huguet-Moreno I, Marazuela Azpiroz M. A practical approach to normocalcemic primary hyperparathyroidism. Endocrine. 2021;74(2):235‐244. 34386939 10.1007/s12020-021-02845-4

[104] Rao DS, Wilson RJ, Kleerekoper M, Parfitt AM. Lack of biochemical progression or continuation of accelerated bone loss in mild asymptomatic primary hyperparathyroidism: evidence for biphasic disease course. J Clin Endocrinol Metab. 1988;67(6):1294‐1298. 3192682 10.1210/jcem-67-6-1294

[105] Bollerslev J, Jansson S, Mollerup CL, et al Medical observation, compared with parathyroidectomy, for asymptomatic primary hyperparathyroidism: a prospective, randomized trial. J Clin Endocrinol Metab. 2007;92(5):1687‐1692. 17284629 10.1210/jc.2006-1836

[106] Rao DS, Phillips ER, Divine GW, Talpos GB. Randomized controlled clinical trial of surgery *Versus* No surgery in patients with mild asymptomatic primary hyperparathyroidism. J Clin Endocrinol Metab. 2004;89(11):5415‐5422. 15531491 10.1210/jc.2004-0028

[107] Adami S, Braga V, Squaranti R, Rossini M, Gatti D, Zamberlan N. Bone measurements in asymptomatic primary hyperparathyroidism. Bone. 1998;22(5):565‐570. 9600793 10.1016/s8756-3282(98)00042-8

[108] Karlafti E, Dontas I, Lambrinoudaki I, et al Site specific differences in vBMD and geometry in postmenopausal women with primary hyperparathyroidism. Endocrine. 2023;83(1):205‐213. 37597095 10.1007/s12020-023-03491-8

[109] Jung KY, Hong AR, Lee DH, et al The natural history and hip geometric changes of primary hyperparathyroidism without parathyroid surgery. J Bone Miner Metab. 2017;35(3):278‐288. 27038988 10.1007/s00774-016-0751-1

[110] Rubin MR, Bilezikian JP, McMahon DJ, et al The natural history of primary hyperparathyroidism with or without parathyroid surgery after 15 years. J Clin Endocrinol Metab. 2008;93(9):3462‐3470. 18544625 10.1210/jc.2007-1215 PMC2567863

[111] Thakker R V, Newey PJ, Walls G V, et al Clinical practice guidelines for multiple endocrine neoplasia type 1 (MEN1). J Clin Endocrinol Metab. 2012;97(9):2990‐3011. 22723327 10.1210/jc.2012-1230

[112] Maraghelli D, Giusti F, Marini F, Brandi ML. Bone tissue and mineral metabolism in hereditary endocrine tumors: clinical manifestations and genetic bases. Orphanet J Rare Dis. 2020;15(1):102. 32326947 10.1186/s13023-020-01380-1 PMC7181496

[113] Eller-Vainicher C, Chiodini I, Battista C, et al Sporadic and MEN1-related primary hyperparathyroidism: differences in clinical expression and severity. J Bone Miner Res. 2009;24(8):1404‐1410. 19309299 10.1359/jbmr.090304

[114] Kann PH, Bartsch D, Langer P, et al Peripheral bone mineral density in correlation to disease-related predisposing conditions in patients with multiple endocrine neoplasia type 1. J Endocrinol Invest. 2012;35(6):573‐579. 21791969 10.3275/7880

[115] Yavropoulou MP, Vlachou S, Tsoli M, Fostira F, Kaltsas G, Kassi E. Management and long-term follow-up of hyperparathyroidism in multiple endocrine neoplasia type 1: single center experience. J Clin Med. 2022;11(7):1967. 35407574 10.3390/jcm11071967 PMC8999236

[116] Burgess JR . Osteoporosis in multiple endocrine neoplasia type 1. Archives of Surgery. 1999;134(10):1119. 10522858 10.1001/archsurg.134.10.1119

[117] Wang W, Nie M, Jiang Y, et al Impaired geometry, volumetric density, and microstructure of cortical and trabecular bone assessed by HR-pQCT in both sporadic and MEN1-related primary hyperparathyroidism. Osteoporos Int. 2020;31(1):165‐173. 31642976 10.1007/s00198-019-05186-1

[118] Lourenço DM, Coutinho FL, Toledo RA, Montenegro FL, Correia-Deur JE, Toledo SP. Early-onset, progressive, frequent, extensive, and severe bone mineral and renal complications in multiple endocrine neoplasia type 1-associated primary hyperparathyroidism. J Bone Miner Res. 2010;25(11):2382‐2391. 20499354 10.1002/jbmr.125

[119] Song A, Chen R, Guan W, et al Trabecular bone score as a more sensitive tool to evaluate bone involvement in MEN1-related primary hyperparathyroidism. J Clin Endocrinol Metab. 2023;109(1):135‐142. 37539859 10.1210/clinem/dgad460

[120] Kanazawa I, Canaff L, Abi Rafeh J, et al Osteoblast menin regulates bone mass in vivo. J Biol Chem. 2015;290(7):3910‐3924. 25538250 10.1074/jbc.M114.629899 PMC4326801

[121] Aziz A, Miyake T, Engleka KA, Epstein JA, McDermott JC. Menin expression modulates mesenchymal cell commitment to the myogenic and osteogenic lineages. Dev Biol. 2009;332(1):116‐130. 19464283 10.1016/j.ydbio.2009.05.555

[122] Malone JP, Srivastava A, Khardori R. Hyperparathyroidism and multiple endocrine neoplasia. Otolaryngol Clin North Am. 2004;37(4):715‐736. 15262511 10.1016/j.otc.2004.02.005

[123] Li I, Hartley IR, Klubo-Gwiedzdzinska J, et al Fracture risk in pediatric patients with MEN2B. J Clin Endocrinol Metab. 2022;107(12):e4371‐e4378. 36056624 10.1210/clinem/dgac500 PMC10233495

[124] Newey PJ, Hannan FM, Wilson A, Thakker R V. Genetics of monogenic disorders of calcium and bone metabolism. Clin Endocrinol (Oxf). 2022;97(4):483‐501. 34935164 10.1111/cen.14644 PMC7614875

[125] English KA, Lines KE, Thakker RV. Genetics of hereditary forms of primary hyperparathyroidism. Hormones. 2024;23(1):3‐14. 38038882 10.1007/s42000-023-00508-9 PMC10847196

[126] Tőke J, Czirják G, Enyedi P, Tóth M. Rare diseases caused by abnormal calcium sensing and signalling. Endocrine. 2021;71(3):611‐617. 33528764 10.1007/s12020-021-02620-5 PMC8016752

[127] Christensen SE, Nissen PH, Vestergaard P, et al Skeletal consequences of familial hypocalciuric hypercalcaemia *vs.* Primary hyperparathyroidism. Clin Endocrinol (Oxf). 2009;71(6):798‐807. 19250271 10.1111/j.1365-2265.2009.03557.x

[128] Isaksen T, Nielsen CS, Christensen SE, Nissen PH, Heickendorff L, Mosekilde L. Forearm bone mineral density in familial hypocalciuric hypercalcemia and primary hyperparathyroidism: a comparative study. Calcif Tissue Int. 2011;89(4):285‐294. 21785908 10.1007/s00223-011-9517-x

[129] Mouly C, Vargas-Poussou R, Lienhardt A, et al Clinical characteristics of familial hypocalciuric hypercalcaemia type 1: a multicentre study of 77 adult patients. Clin Endocrinol (Oxf). 2020;93(3):248‐260. 32347971 10.1111/cen.14211

[130] Jakobsen NFB, Rolighed L, Moser E, Nissen PH, Mosekilde L, Rejnmark L. Increased trabecular volumetric bone mass density in familial hypocalciuric hypercalcemia (FHH) type 1: a cross-sectional study. Calcif Tissue Int. 2014;95(2):141‐152. 24894639 10.1007/s00223-014-9877-0

[131] Vargas-Poussou R, Mansour-Hendili L, Baron S, et al Familial hypocalciuric hypercalcemia types 1 and 3 and primary hyperparathyroidism: similarities and differences. J Clin Endocrinol Metab. 2016;101(5):2185‐2195. 26963950 10.1210/jc.2015-3442

[132] Hannan FM, Howles SA, Rogers A, et al Adaptor protein-2 sigma subunit mutations causing familial hypocalciuric hypercalcaemia type 3 (FHH3) demonstrate genotype–phenotype correlations, codon bias and dominant-negative effects. Hum Mol Genet. 2015;24(18):5079‐5092. 26082470 10.1093/hmg/ddv226 PMC4550820

[133] Ye Z, Silverberg SJ, Sreekanta A, et al The efficacy and safety of medical and surgical therapy in patients with primary hyperparathyroidism: a systematic review and meta-analysis of randomized controlled trials. J Bone Miner Res. 2022;37(11):2351‐2372. 36053960 10.1002/jbmr.4685

[134] Edwards ME, Rotramel A, Beyer T, et al Improvement in the health-related quality-of-life symptoms of hyperparathyroidism is durable on long-term follow-up. Surgery. 2006;140(4):655‐664. 17011914 10.1016/j.surg.2006.06.016

[135] Yeh MW, Zhou H, Adams AL, et al The relationship of parathyroidectomy and bisphosphonates with fracture risk in primary hyperparathyroidism. Ann Intern Med. 2016;164(11):715‐723. 27043778 10.7326/M15-1232

[136] Miyaoka D, Imanishi Y, Kato E, et al Effects of denosumab as compared with parathyroidectomy regarding calcium, renal, and bone involvement in osteoporotic patients with primary hyperparathyroidism. Endocrine. 2020;69(3):642‐649. 32621048 10.1007/s12020-020-02401-6

[137] Cipriani C, Abraham A, Silva BC, et al Skeletal changes after restoration of the euparathyroid state in patients with hypoparathyroidism and primary hyperparathyroidism. Endocrine. 2017;55(2):591‐598. 27757772 10.1007/s12020-016-1101-8 PMC5407087

[138] Jones AR, Simons K, Harvey S, Grill V. Bone mineral density compared to trabecular bone score in primary hyperparathyroidism. J Clin Med. 2022;11(2):330. 35054024 10.3390/jcm11020330 PMC8781599

[139] VanderWalde LH . The effect of parathyroidectomy on bone fracture risk in patients with primary hyperparathyroidism. Archives of Surgery. 2006;141(9):885. 16983032 10.1001/archsurg.141.9.885

[140] Seib CD, Meng T, Suh I, et al Risk of fracture among older adults with primary hyperparathyroidism receiving parathyroidectomy vs nonoperative management. JAMA Intern Med. 2022;182(1):10‐18. 34842909 10.1001/jamainternmed.2021.6437 PMC8630642

[141] Agarwal G, Mishra SK, Kar DK, et al Recovery pattern of patients with osteitis fibrosa cystica in primary hyperparathyroidism after successful parathyroidectomy. Surgery. 2002;132(6):1075‐1085. 12490858 10.1067/msy.2002.128484

[142] de Oliveira FM, Makimoto TE, Scalissi NM, Marone MMS, Maeda SS. Regression of orbital brown tumor after surgical removal of parathyroid adenoma. Arch Endocrinol Metab. 2015;59(5):455‐459. 26331231 10.1590/2359-3997000000088

[143] Fedhila M, Belkacem Chebil R, Marmouch H, et al Brown tumors of the jaws: a retrospective study. Clin Med Insights Endocrinol Diabetes. 2023;16:11795514231210143. 37942058 10.1177/11795514231210143 PMC10629299

[144] Koumakis E, Souberbielle JC, Sarfati E, et al Bone mineral density evolution after successful parathyroidectomy in patients with normocalcemic primary hyperparathyroidism. J Clin Endocrinol Metab. 2013;98(8):3213‐3220. 23783096 10.1210/jc.2013-1518

[145] Ambrogini E, Cetani F, Cianferotti L, et al Surgery or surveillance for mild asymptomatic primary hyperparathyroidism: a prospective, randomized clinical trial. J Clin Endocrinol Metab. 2007;92(8):3114‐3121. 17535997 10.1210/jc.2007-0219

[146] Pappachan JM, Lahart IM, Viswanath AK, et al Parathyroidectomy for adults with primary hyperparathyroidism. Cochrane Database Syst Rev. 2023;3(3):CD013035. 36883976 10.1002/14651858.CD013035.pub2 PMC9995748

[147] Frey S, Gérard M, Guillot P, et al Parathyroidectomy improves bone density in women with primary hyperparathyroidism and preoperative osteopenia. J Clin Endocrinol Metab. 2024;109(6):1494‐1504. 38152848 10.1210/clinem/dgad718

[148] Lundstam K, Heck A, Godang K, et al Effect of surgery versus observation: skeletal 5-year outcomes in a randomized trial of patients with primary HPT (the SIPH study). J Bone Miner Res. 2017;32(9):1907‐1914. 28543873 10.1002/jbmr.3177

[149] Lundstam K, Pretorius M, Bollerslev J, et al Positive effect of parathyroidectomy compared to observation on BMD in a randomized controlled trial of mild primary hyperparathyroidism. J Bone Miner Res. 2023;38(3):372‐380. 36593641 10.1002/jbmr.4763

[150] Pretorius M, Lundstam K, Heck A, et al Mortality and morbidity in mild primary hyperparathyroidism: results from a 10-year prospective randomized controlled trial of parathyroidectomy versus observation. Ann Intern Med. 2022;175(6):812‐819. 35436153 10.7326/M21-4416

[151] Kongsaree N, Thanyajaroen T, Dechates B, Therawit P, Mahikul W, Ngaosuwan K. Skeletal effect of parathyroidectomy on patients with primary hyperparathyroidism: a systematic review and meta-analysis. J Clin Endocrinol Metab. 2024;109(10):e1922‐e1935. 38739762 10.1210/clinem/dgae326

[152] Cironi KA, Issa PP, Albuck AL, et al Comparison of medical management versus parathyroidectomy in patients with mild primary hyperparathyroidism: a meta-analysis. Cancers (Basel). 2023;15(12):3085. 37370696 10.3390/cancers15123085 PMC10296026

[153] Silva AM, Vodopivec D, Christakis I, et al Operative intervention for primary hyperparathyroidism offers greater bone recovery in patients with sporadic disease than in those with multiple endocrine neoplasia type 1–related hyperparathyroidism. Surgery. 2017;161(1):107‐115. 27842919 10.1016/j.surg.2016.06.065

[154] Cristina EV, Alberto F. Management of familial hyperparathyroidism syndromes: MEN1, MEN2, MEN4, HPT-jaw tumour, familial isolated hyperparathyroidism, FHH, and neonatal severe hyperparathyroidism. Best Pract Res Clin Endocrinol Metab. 2018;32(6):861‐875. 30665551 10.1016/j.beem.2018.09.010

[155] Witteveen JE, van Thiel S, Romijn JA, Hamdy NAT. THERAPY OF ENDOCRINE DISEASE: hungry bone syndrome: still a challenge in the post-operative management of primary hyperparathyroidism: a systematic review of the literature. Eur J Endocrinol. 2013;168(3):R45‐R53. 23152439 10.1530/EJE-12-0528

[156] Salman MA, Rabiee A, Salman AA, et al Role of vitamin D supplements in prevention of hungry bone syndrome after successful parathyroidectomy for primary hyperparathyroidism: a prospective study. Scand J Surg. 2021;110(3):329‐334. 33019891 10.1177/1457496920962601

[157] Carsote M, Nistor C. Forestalling hungry bone syndrome after parathyroidectomy in patients with primary and renal hyperparathyroidism. Diagnostics. 2023;13(11):1953. 37296804 10.3390/diagnostics13111953 PMC10252569

[158] Mayilvaganan S, Vijaya Sarathi H, Shivaprasad C. Preoperative zoledronic acid therapy prevent hungry bone syndrome in patients with primary hyperparathyroidism. Indian J Endocrinol Metab. 2017;21(1):76‐79. 28217502 10.4103/2230-8210.196023 PMC5240085

[159] Pal R, Gautam A, Bhadada SK. Role of bisphosphonates in the prevention of postoperative hungry bone syndrome in primary hyperparathyroidism: a meta-analysis and need for randomized controlled trials. Drug Res. 2021;71(2):108‐109. 10.1055/a-1325-0351 33296924

[160] Leere JS, Karmisholt J, Robaczyk M, Vestergaard P. Contemporary medical management of primary hyperparathyroidism: a systematic review. Front Endocrinol (Lausanne). 2017;8:79. 28473803 10.3389/fendo.2017.00079 PMC5397399

[161] Bandeira F, Nóbrega JdM, de Oliveira LB, Bilezikian J. Medical management of primary hyperparathyroidism. Arch Endocrinol Metab. 2022;66(5):689‐693. 36382758 10.20945/2359-3997000000558 PMC10118813

[162] Eller-Vainicher C, Palmieri S, Cairoli E, et al Protective effect of denosumab on bone in older women with primary hyperparathyroidism. J Am Geriatr Soc. 2018;66(3):518‐524. 29364518 10.1111/jgs.15250

[163] Peacock M, Bolognese MA, Borofsky M, et al Cinacalcet treatment of primary hyperparathyroidism: biochemical and bone densitometric outcomes in a five-year study. J Clin Endocrinol Metab. 2009;94(12):4860‐4867. 19837909 10.1210/jc.2009-1472

[164] Shoback DM, Bilezikian JP, Turner SA, McCary LC, Guo MD, Peacock M. The calcimimetic cinacalcet normalizes Serum calcium in subjects with primary hyperparathyroidism. J Clin Endocrinol Metab. 2003;88(12):5644‐5649. 14671147 10.1210/jc.2002-021597

[165] Peacock M, Bilezikian JP, Bolognese MA, et al Cinacalcet HCl reduces hypercalcemia in primary hyperparathyroidism across a wide Spectrum of disease severity. J Clin Endocrinol Metab. 2011;96(1):E9‐E18. 20943783 10.1210/jc.2010-1221 PMC3203649

[166] Gallagher JC, Nordin BEC. TREATMENT WITH ŒSTROGENS OF PRIMARY HYPERPARATHYROIDISM IN POST-MENOPAUSAL WOMEN. The Lancet. 1972;299(7749):503‐507. 10.1016/s0140-6736(72)90173-0 4110019

[167] Grey AB . Effect of hormone replacement therapy on bone mineral density in postmenopausal women with mild primary hyperparathyroidism. Ann Intern Med. 1996;125(5):360‐368. 8702086 10.7326/0003-4819-125-5-199609010-00002

[168] Orr-Walker BJ, Evans MC, Clearwater JM, Horne A, Grey AB, Reid IR. Effects of hormone replacement therapy on bone mineral density in postmenopausal women with primary hyperparathyroidism. Arch Intern Med. 2000;160(14):2161. 10904459 10.1001/archinte.160.14.2161

[169] Zanchetta JR, Bogado CE. Raloxifene reverses bone loss in postmenopausal women with mild asymptomatic primary hyperparathyroidism. J Bone Miner Res. 2001;16(1):189‐190. 11149484 10.1359/jbmr.2001.16.1.189

[170] Akbaba G, Isik S, Ates Tutuncu Y, Ozuguz U, Berker D, Guler S. Comparison of alendronate and raloxifene for the management of primary hyperparathyroidism. J Endocrinol Invest. 2013;36(11):1076‐1082. 24081023 10.3275/9095

[171] Faggiano A, Di Somma C, Ramundo V, et al Cinacalcet hydrochloride in combination with alendronate normalizes hypercalcemia and improves bone mineral density in patients with primary hyperparathyroidism. Endocrine. 2011;39(3):283‐287. 21445714 10.1007/s12020-011-9459-0

[172] Leere JS, Karmisholt J, Robaczyk M, et al Denosumab and cinacalcet for primary hyperparathyroidism (DENOCINA): a randomised, double-blind, placebo-controlled, phase 3 trial. Lancet Diabetes Endocrinol. 2020;8(5):407‐417. 32333877 10.1016/S2213-8587(20)30063-2

[173] Jorde R, Szumlas K, Haug E, Sundsfjord J. The effects of calcium supplementation to patients with primary hyperparathyroidism and a low calcium intake. Eur J Nutr. 2002;41(6):258‐263. 12474069 10.1007/s00394-002-0383-1

[174] Shah VN, Shah CS, Bhadada SK, Rao DS. Effect of 25 (OH) D replacements in patients with primary hyperparathyroidism (PHPT) and coexistent vitamin D deficiency on serum 25(OH) D, calcium and PTH levels: a meta-analysis and review of literature. Clin Endocrinol (Oxf). 2014;80(6):797‐803. 24382124 10.1111/cen.12398

[175] Song A, Zhao H, Yang Y, et al Safety and efficacy of common vitamin D supplementation in primary hyperparathyroidism and coexistent vitamin D deficiency and insufficiency: a systematic review and meta-analysis. J Endocrinol Invest. 2021;44(8):1667‐1677. 33453021 10.1007/s40618-020-01473-5

[176] Dandurand K, Ali DS, Khan AA. Primary hyperparathyroidism: a narrative review of diagnosis and medical management. J Clin Med. 2021;10(8):1604. 33918966 10.3390/jcm10081604 PMC8068862

[177] Khan AA, Bilezikian JP, Kung AWC, et al Alendronate in primary hyperparathyroidism: a double-blind, randomized, placebo-controlled trial. J Clin Endocrinol Metab. 2004;89(7):3319‐3325. 15240609 10.1210/jc.2003-030908

[178] Rossini M, Gatti D, Isaia G, Sartori L, Braga V, Adami S. Effects of oral alendronate in elderly patients with osteoporosis and mild primary hyperparathyroidism. J Bone Miner Res. 2001;16(1):113‐119. 11149474 10.1359/jbmr.2001.16.1.113

[179] Chandran M, Bilezikian JP, Lau J, et al The efficacy and safety of cinacalcet in primary hyperparathyroidism: a systematic review and meta-analysis of randomized controlled trials and cohort studies. Rev Endocr Metab Disord. 2022;23(3):485‐501. 35041148 10.1007/s11154-021-09694-6

[180] Rodan GA . Bisphosphonates and primary hyperparathyroidism. J Bone Miner Res. 2002;17(Suppl 2):N150‐N153. 12412793

[181] Misiorowski W . [Alendronate increases bone mineral density in patients with symptomatic primary hyperparathyroidism]. Endokrynol Pol. 2005;56(6):871‐875. 16821204

[182] Cesareo R, Di Stasio E, Vescini F, et al Effects of alendronate and vitamin D in patients with normocalcemic primary hyperparathyroidism. Osteoporos Int. 2015;26(4):1295‐1302. 25524023 10.1007/s00198-014-3000-2

[183] Reasner CA, Stone MD, Hosking DJ, Ballah A, Mundy GR. Acute changes in calcium homeostasis during treatment of primary hyperparathyroidism with risedronate. J Clin Endocrinol Metab. 1993;77(4):1067‐1071. 8408454 10.1210/jcem.77.4.8408454

[184] Tournis S, Fakidari E, Dontas I, et al Effect of parathyroidectomy versus risedronate on volumetric bone mineral density and bone geometry at the tibia in postmenopausal women with primary hyperparathyroidism. J Bone Miner Metab. 2014;32(2):151‐158. 23700284 10.1007/s00774-013-0473-6

[185] Cefalu CA . Is bone mineral density predictive of fracture risk reduction? Curr Med Res Opin. 2004;20(3):341‐349. 15025843 10.1185/030079903125003062

[186] Rajput S, Dutta A, Rajender S, Mithal A, Chattopadhyay N. Efficacy of antiresorptive agents bisphosphonates and denosumab in mitigating hypercalcemia and bone loss in primary hyperparathyroidism: a systematic review and meta-analysis. Front Endocrinol (Lausanne). 2023;14:1098841. 36817591 10.3389/fendo.2023.1098841 PMC9931892

[187] Deeks ED . Denosumab: a review in postmenopausal osteoporosis. Drugs Aging. 2018;35(2):163‐173. 29435849 10.1007/s40266-018-0525-7

[188] Gronskaya S, Belaya Z, Rozhinskaya L, et al Denosumab for osteoporosis in patients with primary hyperparathyroidism and mild-to-moderate renal insufficiency. Endocrine. 2023;81(2):368‐378. 37133642 10.1007/s12020-023-03381-z

[189] Thongprayoon C, Acharya P, Acharya C, et al Hypocalcemia and bone mineral density changes following denosumab treatment in end-stage renal disease patients: a meta-analysis of observational studies. Osteoporos Int. 2018;29(8):1737‐1745. 29713798 10.1007/s00198-018-4533-6

[190] Kim KJ, Hong N, Lee S, Kim M, Rhee Y. A simple-to-use score for identifying individuals at high risk of denosumab-associated hypocalcemia in postmenopausal osteoporosis: a real-world cohort study. Calcif Tissue Int. 2020;107(6):567‐575. 32920682 10.1007/s00223-020-00754-8

[191] Bird ST, Smith ER, Gelperin K, et al Severe hypocalcemia with denosumab among older female dialysis-dependent patients. JAMA. 2024;331(6):491‐499. 38241060 10.1001/jama.2023.28239 PMC10799290

[192] Kuchay MS, Mathew A, Kaur P, Mishra SK. Denosumab can be used successfully as a bridge to surgery in patients with severe hypercalcemia due to primary hyperparathyroidism. Arch Endocrinol Metab. 2021;65(5):669‐673. 34591412 10.20945/2359-3997000000408 PMC10528581

[193] Rajan R, Cherian K, Kapoor N, Paul T. Denosumab as a bridge to surgery in a patient with severe hypercalcemia due to primary hyperparathyroidism in the setting of renal dysfunction. Indian J Endocrinol Metab. 2019;23(2):269‐270. 31161118 10.4103/ijem.IJEM_678_18 PMC6540887

[194] Li Y, Fan CY, Manni A, Simonds WF. Pitfalls of using denosumab preoperatively to treat refractory severe hypercalcaemia. BMJ Case Rep. 2020;13(4):e233665. 10.1136/bcr-2019-233665 PMC7213692 32350052

[195] Polyzos SA, Makras P, Tournis S, Anastasilakis AD. Off-label uses of denosumab in metabolic bone diseases. Bone. 2019;129:115048. 31454537 10.1016/j.bone.2019.115048

[196] Konrade I, Dambrova G, Dambrova M. Denosumab-induced hypophosphataemia in a case of normocalcaemic primary hyperparathyroidism. Intern Med J. 2017;47(8):974‐975. 28782221 10.1111/imj.13508

[197] Duntas LH, Stathatos N. Cinacalcet as alternative treatment for primary hyperparathyroidism: achievements and prospects. Endocrine. 2011;39(3):199‐204. 21442382 10.1007/s12020-011-9452-7

[198] Minezaki M, Takashi Y, Ochi K, et al Reduction in parathyroid adenomas by cinacalcet therapy in patients with primary hyperparathyroidism. J Bone Miner Metab. 2021;39(4):583‐588. 33409573 10.1007/s00774-020-01190-2

[199] Giusti F, Cianferotti L, Gronchi G, et al Cinacalcet therapy in patients affected by primary hyperparathyroidism associated to multiple endocrine neoplasia syndrome type 1 (MEN1). Endocrine. 2016;52(3):495‐506. 26224587 10.1007/s12020-015-0696-5

[200] Nagano N . Pharmacological and clinical properties of calcimimetics: calcium receptor activators that afford an innovative approach to controlling hyperparathyroidism. Pharmacol Ther. 2006;109(3):339‐365. 16102839 10.1016/j.pharmthera.2005.06.019

[201] Sajid-Crockett S, Singer FR, Hershman JM. Cinacalcet for the treatment of primary hyperparathyroidism. Metabolism. 2008;57(4):517‐521. 18328354 10.1016/j.metabol.2007.11.014

[202] Iglesias P, Ais G, González A, et al Acute and one-year effects of cinacalcet in patients with persistent primary hyperparathyroidism after unsuccessful parathyroidectomy. Am J Med Sci. 2008;335(2):111‐114. 18277118 10.1097/MAJ.0b013e3181379f3e

[203] Bell D, Hale J, Go C, et al A single-centre retrospective analysis of cinacalcet therapy in primary hyperparathyroidism. Endocr Connect. 2021;10(11):1435‐1444. 34647901 10.1530/EC-21-0258 PMC8630765

[204] Khan A, Bilezikian J, Bone H, et al Cinacalcet normalizes serum calcium in a double-blind randomized, placebo-controlled study in patients with primary hyperparathyroidism with contraindications to surgery. Eur J Endocrinol. 2015;172(5):527‐535. 25637076 10.1530/EJE-14-0877 PMC5729741

[205] Ng CH, Chin YH, Tan MHQ, et al Cinacalcet and primary hyperparathyroidism: systematic review and meta regression. Endocr Connect. 2020;9(7):724‐735. 32621588 10.1530/EC-20-0221 PMC7424342

[206] Schwarz P, Body JJ, Cáp J, et al The PRIMARA study: a prospective, descriptive, observational study to review cinacalcet use in patients with primary hyperparathyroidism in clinical practice. Eur J Endocrinol. 2014;171(6):727‐735. 25240499 10.1530/EJE-14-0355

[207] Luque-Fernández I, García-Martín A, Luque-Pazos A. Experience with cinacalcet in primary hyperparathyroidism: results after 1 year of treatment. Ther Adv Endocrinol Metab. 2013;4(3):77‐81. 23730501 10.1177/2042018813482344 PMC3666442

[208] Manaka K, Sato J, Kinoshita Y, et al Effectiveness and safety of cinacalcet for primary hyperparathyroidism: a single center experience. Endocr J. 2019;66(8):683‐689. 31092749 10.1507/endocrj.EJ19-0034

[209] Khan A, Grey A, Shoback D. Medical management of asymptomatic primary hyperparathyroidism: proceedings of the third international workshop. J Clin Endocrinol Metab. 2009;94(2):373‐381. 19193912 10.1210/jc.2008-1762

[210] Komaba H, Nakanishi S, Fujimori A, et al Cinacalcet effectively reduces parathyroid hormone secretion and gland volume regardless of pretreatment gland size in patients with secondary hyperparathyroidism. Clin J Am Soc Nephrol. 2010;5(12):2305‐2314. 20798251 10.2215/CJN.02110310 PMC2994093

[211] Ichii M, Ishimura E, Okuno S, et al Decreases in parathyroid gland volume after cinacalcet treatment in hemodialysis patients with secondary hyperparathyroidism. Nephron Clin Pract. 2010;115(3):c195‐c202. 20413997 10.1159/000313035

[212] Meola M, Petrucci I, Barsotti G. Long-term treatment with cinacalcet and conventional therapy reduces parathyroid hyperplasia in severe secondary hyperparathyroidism. Nephrology Dialysis Transplantation. 2008;24(3):982‐989. 10.1093/ndt/gfn654 PMC2644631 19181759

[213] Tatsumi R, Komaba H, Kanai G, et al Cinacalcet induces apoptosis in parathyroid cells in patients with secondary hyperparathyroidism: histological and cytological analyses. Nephron Clin Pract. 2014;124(3-4):224‐231. 10.1159/000357951 24503607

[214] Nguyen S, Gosmanova EO, Gosmanov AR. Cinacalcet-associated resolution of primary hyperparathyroidism in a patient with normal kidney function. J Investig Med High Impact Case Rep. 2020;8:2324709620936836. 10.1177/2324709620936836 PMC7318811 32583691

[215] Falchetti A, Cilotti A, Vagelli L, et al A patient with MEN1-associated hyperparathyroidism, responsive to cinacalcet. Nat Clin Pract Endocrinol Metab. 2008;4(6):351‐357. 18414463 10.1038/ncpendmet0816

[216] Filopanti M, Verga U, Ermetici F, et al MEN1-related hyperparathyroidism: response to cinacalcet and its relationship with the calcium-sensing receptor gene variant Arg990Gly. Eur J Endocrinol. 2012;167(2):157‐164. 22577108 10.1530/EJE-12-0117

[217] Moyes VJ, Monson JP, Chew SL, Akker SA. Clinical use of cinacalcet in MEN1 hyperparathyroidism. Int J Endocrinol. 2010;2010:906163. 20585352 10.1155/2010/906163 PMC2877200

[218] Paik JM, Curhan GC, Taylor EN. Calcium intake and risk of primary hyperparathyroidism in women: prospective cohort study. BMJ. 2012;345:e6390. 23080543 10.1136/bmj.e6390 PMC3475985

[219] Locker REFG, Silverberg MSJ, Bilezikian MJP. Optimal dietary calcium intake in primary hyperparathyroidism. Am J Med. 1997;102(6):543‐550. 9217669 10.1016/s0002-9343(97)00053-3

[220] Moosgaard B, Vestergaard P, Heickendorff L, Melsen F, Christiansen P, Mosekilde L. Vitamin D status, seasonal variations, parathyroid adenoma weight and bone mineral density in primary hyperparathyroidism. Clin Endocrinol (Oxf). 2005;63(5):506‐513. 16268801 10.1111/j.1365-2265.2005.02371.x

[221] Meng L, Su C, Shapses SA, Al-Dayyeni A, He Y, Wang X. Lower total 25-hydroxyvitamin D but no difference in calculated or measured free 25-hydroxyvitamin D serum levels in patients with primary hyperparathyroidism. J Steroid Biochem Mol Biol. 2020;199:105616. 32027935 10.1016/j.jsbmb.2020.105616

[222] Meng L, Su C, Shapses SA, Wang X. Total and free vitamin D metabolites in patients with primary hyperparathyroidism. J Endocrinol Invest. 2022;45(2):301‐307. 34282553 10.1007/s40618-021-01633-1

[223] Acharya R, Kopczynska M, Goodmaker C, Mukherjee A, Doran H. Vitamin D repletion in primary hyperparathyroid patients undergoing parathyroidectomy leads to reduced symptomatic hypocalcaemia and reduced length of stay: a retrospective cohort study. Ann R Coll Surg Engl. 2022;104(1):41‐47. 34727512 10.1308/rcsann.2021.0078 PMC10335014

[224] Unsal IO, Calapkulu M, Sencar ME, et al Preoperative vitamin D levels as a predictor of transient hypocalcemia and hypoparathyroidism after parathyroidectomy. Sci Rep. 2020;10(1):9895. 32555278 10.1038/s41598-020-66889-8 PMC7303145

[225] Rao SD, Miragaya J, Parikh N, et al Effect of vitamin D nutrition on disease indices in patients with primary hyperparathyroidism. J Steroid Biochem Mol Biol. 2020;201:105695. 32407867 10.1016/j.jsbmb.2020.105695

[226] Marcocci C, Bollerslev J, Khan AA, Shoback DM. Medical management of primary hyperparathyroidism: proceedings of the fourth international workshop on the management of asymptomatic primary hyperparathyroidism. J Clin Endocrinol Metab. 2014;99(10):3607‐3618. 25162668 10.1210/jc.2014-1417

[227] Lee JH, Kim JH, Hong AR, Kim SW, Shin CS. Skeletal effects of vitamin D deficiency among patients with primary hyperparathyroidism. Osteoporos Int. 2017;28(5):1667‐1674. 28175978 10.1007/s00198-017-3918-2

[228] Walker MD, Cong E, Lee JA, et al Vitamin D in primary hyperparathyroidism: effects on clinical, biochemical, and densitometric presentation. J Clin Endocrinol Metab. 2015;100(9):3443‐3451. 26079779 10.1210/jc.2015-2022 PMC4570160

[229] Walker MD, Nishiyama KK, Zhou B, et al Effect of low vitamin D on volumetric bone mineral density, bone microarchitecture, and stiffness in primary hyperparathyroidism. J Clin Endocrinol Metab. 2016;101(3):905‐913. 26745256 10.1210/jc.2015-4218 PMC4803169

[230] Loh HH, Lim LL, Yee A, Loh HS, Vethakkan SR. Effect of vitamin D replacement in primary hyperparathyroidism with concurrent vitamin D deficiency: a systematic review and meta-analysis. Minerva Endocrinol. 2019;44(2):221‐231. 28294593 10.23736/S0391-1977.17.02584-6

[231] Ryhänen EM, Koski AM, Löyttyniemi E, Välimäki MJ, Kiviniemi U, Schalin-Jäntti C. Postoperative zoledronic acid for osteoporosis in primary hyperparathyroidism: a randomized placebo-controlled study. Eur J Endocrinol. 2021;185(4):515‐524. 34324430 10.1530/EJE-21-0322

[232] Orr LE, Zhou H, Zhu CY, Haigh PI, Adams AL, Yeh MW. Skeletal effects of combined medical and surgical management of primary hyperparathyroidism. Surgery. 2020;167(1):144‐148. 31582307 10.1016/j.surg.2019.04.059

[233] Choe HJ, Koo BK, Yi KH, et al Skeletal effects of combined bisphosphonates treatment and parathyroidectomy in osteoporotic patients with primary hyperparathyroidism. J Bone Miner Metab. 2022;40(2):292‐300. 34761302 10.1007/s00774-021-01279-2

[234] de França TCPT, Griz L, Pinho J, et al Bisphosphonates can reduce bone hunger after parathyroidectomy in patients with primary hyperparathyroidism and osteitis fibrosa cystica. Rev Bras Reumatol. 2011;51(2):131‐137. 21584419

[235] Lee IT, Sheu WHH, Tu ST, Kuo SW, Pei D. Bisphosphonate pretreatment attenuates hungry bone syndrome postoperatively in subjects with primary hyperparathyroidism. J Bone Miner Metab. 2006;24(3):255‐258. 16622740 10.1007/s00774-005-0680-x

[236] Cetani F, Pardi E, Marcocci C. Parathyroid carcinoma. Front Horm Res. 2019;51:63‐76. 30641523 10.1159/000491039

[237] Wei CH, Harari A. Parathyroid carcinoma: update and guidelines for management. Curr Treat Options Oncol. 2012;13(1):11‐23. 22327883 10.1007/s11864-011-0171-3

[238] Naderi N, McCurdy MT, Reed RM. Bone resorption in parathyroid carcinoma. Case Rep. 2014;2014:bcr2013203396. 10.1136/bcr-2013-203396 PMC4039922 24849640

[239] Christmas TJ, Chapple CR, Noble JG, Milroy EJG, Cowie AGA. Hyperparathyroidism after neck irradiation. Br J Surg. 2005;75(9):873‐874. 10.1002/bjs.1800750914 3179662

[240] Ireland JP, Fleming SJ, Levison DA, Cattell WR, Baker LR. Parathyroid carcinoma associated with chronic renal failure and previous radiotherapy to the neck. J Clin Pathol. 1985;38(10):1114‐1118. 4056066 10.1136/jcp.38.10.1114 PMC499451

[241] Khan MW, Worcester EM, Straus FH, Khan S, Staszak V, Kaplan EL. Parathyroid carcinoma in secondary and tertiary hyperparathyroidism1. J Am Coll Surg. 2004;199(2):312‐319. 15275889 10.1016/j.jamcollsurg.2004.04.014

[242] Haghighi P, Astarita RW, Wepsic HT, Wolf PL. Concurrent primary parathyroid hyperplasia and parathyroid carcinoma. Arch Pathol Lab Med. 1983;107(7):349‐350. 6687992

[243] Goldfarb M, O’Neal P, Shih JL, Hartzband P, Connolly J, Hasselgren PO. Synchronous parathyroid carcinoma, parathyroid adenoma, and papillary thyroid carcinoma in a patient with severe and long-standing hyperparathyroidism. Endocr Pract. 2009;15(5):463‐468. 19491068 10.4158/EP09075.CRR PMC2917245

[244] Campennì A, Ruggeri RM, Sindoni A, et al Parathyroid carcinoma presenting as normocalcemic hyperparathyroidism. J Bone Miner Metab. 2012;30(3):367‐372. 22246083 10.1007/s00774-011-0344-y

[245] Shahid A, Iftikhar F. Pathological bone fractures in a patient with parathyroid carcinoma—a case report. J Pak Med Assoc. 2017;67(12):1956‐1958. 29256556

[246] Domínguez-Gasca LG, Colín-González CG, Barroso-Gómez G, Arellano-Aguilar G, Domínguez-Carrillo LG, Aguirre-Trigueros J. [Parathyroid carcinoma: diagnosis by a femur fracture]. Acta Ortop Mex. 2018;32(4):229‐233. 30549507

[247] Rizwan A, Jamal A, Uzzaman M, Fatima S. Case report: lady with bone pains for 5 years—parathyroid carcinoma. BMC Res Notes. 2018;11(1):617. 30157930 10.1186/s13104-018-3711-0 PMC6114890

[248] Suzuki T, Tomiie Y. Radial fracture due to parathyroid carcinoma. N Engl J Med. 2018;379(15):e24. 30304649 10.1056/NEJMicm1710426

[249] Šplíchalová E, Holý R, Traboulsi E, Zadražilová A, Astl J. Nonfunctional parathyroid cancer − a case report. Rozhl Chir. 2021;100(3):133‐137. 33910359 10.33699/PIS.2021.100.3.133-137

[250] Salcuni AS, Cetani F, Guarnieri V, et al Parathyroid carcinoma. Best Pract Res Clin Endocrinol Metab. 2018;32(6):877‐889. 30551989 10.1016/j.beem.2018.11.002

[251] Yong TY, Li JYZ. Mediastinal parathyroid carcinoma presenting with severe skeletal manifestations. J Bone Miner Metab. 2010;28(5):591‐594. 20237944 10.1007/s00774-010-0173-4

[252] Betea D, Potorac I, Beckers A. Parathyroid carcinoma: challenges in diagnosis and treatment. Ann Endocrinol (Paris). 2015;76(2):169‐177. 25910997 10.1016/j.ando.2015.03.003

[253] Lenschow C, Schrägle S, Kircher S, et al Clinical presentation, treatment, and outcome of parathyroid carcinoma. Ann Surg. 2022;275(2):e479‐e487. 32649472 10.1097/SLA.0000000000004144

[254] Radulescu D, Chis B, Donca V, Münteanu V. Brown tumors of the femur and pelvis secondary to a parathyroid carcinoma: report of one case. Rev Med Chil. 2014;142(7):919‐923. 25378013 10.4067/S0034-98872014000700014

[255] Nguyen GN, Nguyen LV. The pathology femoral peritrochanteric fracture with multiple brown tumor as a first sign of parathyroid cancer—a case report. Int J Surg Case Rep. 2021;85:106259. 34343791 10.1016/j.ijscr.2021.106259 PMC8350005

[256] Tsushima Y, Sun S, Via MA. Brown tumors secondary to parathyroid carcinoma masquerading as skeletal metastases on 18F-FDG PET/CT: a case report. AACE Clin Case Rep. 2019;5(4):e230‐e232. 31967041 10.4158/ACCR-2018-0633 PMC6873831

[257] Parikh P, Shetty S, Rodrigues G, Bhat SN. Brown tumour mimicking skeletal metastasis. BMJ Case Rep. 2021;14(7):e243478. 10.1136/bcr-2021-243478 PMC8278892 34257125

[258] Dagang DJT, Gutierrez JB, Sandoval MAS, Lantion-Ang FL. Multiple brown tumours from parathyroid carcinoma. BMJ Case Rep. 2016;2016:bcr2016215961. doi:10.1136/bcr-2016-215961 PMC4932410 27358103

[259] Lin X, Fan Y, Zhang Z, Yue H. Clinical characteristics of primary hyperparathyroidism: 15-year experience of 457 patients in a single center in China. Front Endocrinol (Lausanne). 2021;12:602221. 33716964 10.3389/fendo.2021.602221 PMC7947808

[260] Xue S, Chen H, Lv C, et al Preoperative diagnosis and prognosis in 40 parathyroid carcinoma patients. Clin Endocrinol (Oxf). 2016;85(1):29‐36. 26939543 10.1111/cen.13055

[261] Silverberg SJ, Rubin MR, Faiman C, et al Cinacalcet hydrochloride reduces the Serum calcium concentration in inoperable parathyroid carcinoma. J Clin Endocrinol Metab. 2007;92(10):3803‐3808. 17666472 10.1210/jc.2007-0585

[262] Montenegro FdM, Chammas MC, Juliano AG, Cernea CR, Cordeiro AC. Ethanol injection under ultrasound guidance to palliate unresectable parathyroid carcinoma. Arq Bras Endocrinol Metabol. 2008;52(4):707‐711. 18604386 10.1590/s0004-27302008000400019

[263] Bradwell AR, Harvey TC. Control of hypercalcaemia of parathyroid carcinoma by immunisation. Lancet. 1999;353(9150):370‐373. 9950443 10.1016/S0140-6736(98)06469-1

[264] Betea D, Bradwell AR, Harvey TC, et al Hormonal and biochemical normalization and tumor shrinkage induced by anti-parathyroid hormone immunotherapy in a patient with metastatic parathyroid carcinoma. J Clin Endocrinol Metab. 2004;89(7):3413‐3420. 15240624 10.1210/jc.2003-031911

[265] Sarquis M, Marx SJ, Beckers A, et al Long-term remission of disseminated parathyroid cancer following immunotherapy. Endocrine. 2020;67(1):204‐208. 31782130 10.1007/s12020-019-02136-z PMC9361402

[266] Lenschow C, Fuss CT, Kircher S, et al Case report: abdominal lymph node metastases of parathyroid carcinoma: diagnostic workup, molecular diagnosis, and clinical management. Front Endocrinol (Lausanne). 2021;12:643328. 33833736 10.3389/fendo.2021.643328 PMC8021949

[267] Park D, Airi R, Sherman M. Microsatellite instability driven metastatic parathyroid carcinoma managed with the anti-PD1 immunotherapy, pembrolizumab. BMJ Case Rep. 2020;13(9):e235293. 10.1136/bcr-2020-235293 PMC7513558 32967944

[268] Ta B, Bennett MJ. Refractory hypercalcemia secondary to metastatic parathyroid carcinoma treated with immunotherapy. JCEM Case Rep. 2024;2(7):luae127. 39011405 10.1210/jcemcr/luae127 PMC11247164

[269] Ahmad R, Hammond JM. Primary, secondary, and tertiary hyperparathyroidism. Otolaryngol Clin North Am. 2004;37(4):701‐713. 15262510 10.1016/j.otc.2004.02.004

[270] Naveh-Many T, Volovelsky O. Parathyroid cell proliferation in secondary hyperparathyroidism of chronic kidney disease. Int J Mol Sci. 2020;21(12):4332. 32570711 10.3390/ijms21124332 PMC7352987

[271] Goto S, Komaba H, Fukagawa M. Pathophysiology of parathyroid hyperplasia in chronic kidney disease: preclinical and clinical basis for parathyroid intervention. Clin Kidney J. 2008;1(suppl 3):iii2‐iii8. 10.1093/ndtplus/sfn079 PMC4421132 25983967

[272] Bleskestad IH, Bergrem H, Leivestad T, Gøransson LG. Intact parathyroid hormone levels in renal transplant patients with normal transplant function. Clin Transplant. 2011;25(5):E566‐E570. 21955131 10.1111/j.1399-0012.2011.01515.x

[273] Torregrosa JV, Ferreira AC, Cucchiari D, Ferreira A. Bone mineral disease after kidney transplantation. Calcif Tissue Int. 2021;108(4):551‐560. 33765230 10.1007/s00223-021-00837-0

[274] Jamal SA, Miller PD. Secondary and tertiary hyperparathyroidism. J Clin Densitom. 2013;16(1):64‐68. 23267748 10.1016/j.jocd.2012.11.012

[275] Messa P, Alfieri CM. Secondary and tertiary hyperparathyroidism. Front Horm Res. 2019;51:91‐108. 30641516 10.1159/000491041

[276] Chen H, Han X, Cui Y, Ye Y, Purrunsing Y, Wang N. Parathyroid hormone fragments: new targets for the diagnosis and treatment of chronic kidney disease-mineral and bone disorder. Biomed Res Int. 2018;2018:9619253. 30627584 10.1155/2018/9619253 PMC6304519

[277] Kumari S, Singh PP, Kumar D, Kumar N, Kumar S, Shekhar R. Intact parathyroid hormone (iPTH) assay: an early approach for bone health assessment in chronic renal failure. Cureus. 2024 16(10):e72510. 39606517 10.7759/cureus.72510 PMC11599631

[278] Koizumi M, Komaba H, Fukagawa M. Parathyroid function in chronic kidney disease: role of FGF23-Klotho axis. Contrib Nephrol. 2013;180:110‐123. 23652554 10.1159/000346791

[279] Bover J, Arana C, Ureña P, et al Hyporesponsiveness or resistance to the action of parathyroid hormone in chronic kidney disease. Nefrologia (Engl Ed). 2021;41(5):514‐528. 36165134 10.1016/j.nefroe.2021.11.014

[280] Aguilar A, Gifre L, Ureña-Torres P, et al Pathophysiology of bone disease in chronic kidney disease: from basics to renal osteodystrophy and osteoporosis. Front Physiol. 2023;14:1177829. 37342799 10.3389/fphys.2023.1177829 PMC10277623

[281] Hung KC, Yao WC, Liu YL, et al The potential influence of uremic toxins on the homeostasis of bones and muscles in chronic kidney disease. Biomedicines. 2023;11(7):2076. 37509715 10.3390/biomedicines11072076 PMC10377042

[282] Natikar JA, Shailaja A. Secondary hyperparathyroidism in patients with chronic kidney disease: a case control study. Int J Clin Biochem Res. 2020;7(2):291‐296.

[283] Carneiro Dias RS, José de Araújo Brito D, Milhomem dos Santos E, et al Correlation between parathyroid hormone levels with urinary magnesium excretion in patients with non-dialysis dependent chronic kidney disease. Int J Nephrol Renovasc Dis. 2020;13:341‐348. 33239901 10.2147/IJNRD.S282106 PMC7682596

[284] Liu H, Zhao H, Zheng D, et al Misdiagnosis of chronic kidney disease and parathyroid hormone testing during the past 16 years. Sci Rep. 2023;13(1):15838. 37739989 10.1038/s41598-023-43016-x PMC10516991

[285] Nitta K, Yajima A, Tsuchiya K. Management of osteoporosis in chronic kidney disease. Int Med. 2017;56(24):3271‐3276. 10.2169/internalmedicine.8618-16 PMC5790712 29021477

[286] Williams ME . Chronic kidney disease/bone and mineral metabolism: the imperfect storm. Semin Nephrol. 2009;29(2):97‐104. 19371800 10.1016/j.semnephrol.2009.01.002

[287] Kazama JJ, Wakasugi M. Parathyroid hormone and bone in dialysis patients. Ther Apher Dial. 2018;22(3):229‐235. 29883066 10.1111/1744-9987.12678

[288] Bacchetta J, Boutroy S, Vilayphiou N, et al Early impairment of trabecular microarchitecture assessed with HR-pQCT in patients with stage II-IV chronic kidney disease. J Bone Miner Res. 2010;25(4):849‐57. 19775204 10.1359/jbmr.090831

[289] Kidney Disease: Improving Global Outcomes (KDIGO) CKD-MBD Update Work Group . KDIGO 2017 clinical practice guideline update for the diagnosis, evaluation, prevention, and treatment of chronic kidney disease–mineral and bone disorder (CKD-MBD). Kidney Int Suppl (2011). 2017;7(1):1‐59. 30675420 10.1016/j.kisu.2017.04.001 PMC6340919

[290] Kosowicz J, Bolko P, Swiderski A. [Results of bone scintigraphy, densitometry and radiography in secondary hyperparathyroidism in patients with chronic renal failure]. Pol Arch Med Wewn. 2003;109(2):125‐135. 12879775

[291] Ning JP, Sun M, Toru I, Etsuo Y. [The relationship between bone mineral density and secondary hyperparathyroidism bone disease]. Hunan Yi Ke Da Xue Xue Bao. 2000;25(1):77‐79. 12212259

[292] Tentori F, McCullough K, Kilpatrick RD, et al High rates of death and hospitalization follow bone fracture among hemodialysis patients. Kidney Int. 2014;85(1):166‐173. 23903367 10.1038/ki.2013.279 PMC3910091

[293] Nickolas TL, Leonard MB, Shane E. Chronic kidney disease and bone fracture: a growing concern. Kidney Int. 2008;74(6):721‐731. 18563052 10.1038/ki.2008.264 PMC4139042

[294] Ensrud KE . Renal function and risk of hip and vertebral fractures in older women. Arch Intern Med. 2007;167(2):133. 17242313 10.1001/archinte.167.2.133

[295] Alem AM, Sherrard DJ, Gillen DL, et al Increased risk of hip fracture among patients with end-stage renal disease. Kidney Int. 2000;58(1):396‐399. 10886587 10.1046/j.1523-1755.2000.00178.x

[296] Goto NA, Weststrate ACG, Oosterlaan FM, et al The association between chronic kidney disease, falls, and fractures: a systematic review and meta-analysis. Osteoporos Int. 2020;31(1):13‐29. 31720721 10.1007/s00198-019-05190-5 PMC6946749

[297] Nickolas TL, McMahon DJ, Shane E. Relationship between moderate to severe kidney disease and hip fracture in the United States. J Am Soc Nephrol. 2006;17(11):3223‐3232. 17005938 10.1681/ASN.2005111194

[298] Yamaguchi T, Kanno E, Tsubota J, Shiomi T, Nakai M, Hattori S. Retrospective study on the usefulness of radius and lumbar bone density in the separation of hemodialysis patients with fractures from those without fractures. Bone. 1996;19(5):549‐555. 8922656 10.1016/s8756-3282(96)00246-3

[299] Jamal SA, Chase C, Goh YI, Richardson R, Hawker GA. Bone density and heel ultrasound testing do not identify patients with dialysis-dependent renal failure who have had fractures. Am J Kidney Dis. 2002;39(4):843‐849. 11920352 10.1053/ajkd.2002.32006

[300] Jamal SA, Hayden JA, Beyene J. Low bone mineral density and fractures in long-term hemodialysis patients: a meta-analysis. Am J Kidney Dis. 2007;49(5):674‐681. 17472850 10.1053/j.ajkd.2007.02.264

[301] Tsukamoto Y . [Is it possible to-predict fracture in CKD patients?]. Clin Calcium. 2016;26(9):1295‐1300. 27561344

[302] West SL, Lok CE, Langsetmo L, et al Retracted: bone mineral density predicts fractures in chronic kidney disease. J Bone Miner Res. 2015;30(5):913‐919. 25400209 10.1002/jbmr.2406

[303] Gómez-Islas VE, García-Fong KR, Aguilar-Fuentes RE, et al Evaluation of bone densitometry by dual-energy x-ray absorptiometry as a fracture prediction tool in women with chronic kidney disease. Bone Rep. 2020;13:100298. 32743028 10.1016/j.bonr.2020.100298 PMC7387779

[304] Bucur RC, Panjwani DD, Turner L, Rader T, West SL, Jamal SA. Low bone mineral density and fractures in stages 3–5 CKD: an updated systematic review and meta-analysis. Osteoporos Int. 2015;26(2):449‐458. 25477230 10.1007/s00198-014-2813-3

[305] Iimori S, Mori Y, Akita W, et al Diagnostic usefulness of bone mineral density and biochemical markers of bone turnover in predicting fracture in CKD stage 5D patients—a single-center cohort study. Nephrol Dial Transplant. 2012;27(1):345‐351. 21652550 10.1093/ndt/gfr317

[306] Rampersad C, Whitlock RH, Leslie WD, et al Trabecular bone score in patients with chronic kidney disease. Osteoporos Int. 2020;31(10):1905‐1912. 32440892 10.1007/s00198-020-05458-1

[307] Yun HJ, Ryoo SR, Kim JE, et al Trabecular bone score may indicate chronic kidney disease-mineral and bone disorder (CKD-MBD) phenotypes in hemodialysis patients: a prospective observational study. BMC Nephrol. 2020;21(1):299. 32711466 10.1186/s12882-020-01944-0 PMC7382149

[308] Komaba H, Kakuta T, Fukagawa M. Management of secondary hyperparathyroidism: how and why? Clin Exp Nephrol. 2017;21(S1):37‐45. 28044233 10.1007/s10157-016-1369-2

[309] Singer RF . Vitamin D in dialysis: defining deficiency and rationale for supplementation. Semin Dial. 2013;26(1):40‐46. 23017052 10.1111/sdi.12010

[310] Miskulin DC, Majchrzak K, Tighiouart H, et al Ergocalciferol supplementation in hemodialysis patients with vitamin D deficiency: a randomized clinical trial. J Am Soc Nephrol. 2016;27(6):1801‐1810. 26677862 10.1681/ASN.2015040468 PMC4884115

[311] Okša A, Spustová V, Krivošíková Z, et al Effects of long-term cholecalciferol supplementation on mineral metabolism and calciotropic hormones in chronic kidney disease. Kidney Blood Press Res. 2008;31(5):322‐329. 18802363 10.1159/000157177

[312] Yeung WCG, Palmer SC, Strippoli GFM, et al Vitamin D therapy in adults with CKD: a systematic review and meta-analysis. Am J Kidney Dis. 2023;82(5):543‐558. 37356648 10.1053/j.ajkd.2023.04.003

[313] Bover J, Massó E, Gifre L, et al Vitamin D and chronic kidney disease association with mineral and bone disorder: an appraisal of tangled guidelines. Nutrients. 2023;15(7):1576. 37049415 10.3390/nu15071576 PMC10097233

[314] Schlosser K, Zielke A, Rothmund M. Medical and surgical treatment for secondary and tertiary hyperparathyroidism. Scand J Surg. 2004;93(4):288‐297. 15658670 10.1177/145749690409300407

[315] Wolf M, Thadhani R. VITAMIN D IN HEALTH AND DISEASE: beyond minerals and parathyroid hormone: role of active vitamin D in End-stage renal disease. Semin Dial. 2005;18(4):302‐306. 16076353 10.1111/j.1525-139X.2005.18406.x

[316] Ogata H, Koiwa F, Shishido K, et al Effects of 22-oxacalcitriol and calcitriol on PTH secretion and bone mineral metabolism in a crossover trial in hemodialysis patients with secondary hyperparathyroidism. Ther Apher Dial. 2007;11(3):202‐209. 17498002 10.1111/j.1744-9987.2007.00422.x

[317] Wesseling-Perry K, Pereira RC, Sahney S, et al Calcitriol and doxercalciferol are equivalent in controlling bone turnover, suppressing parathyroid hormone, and increasing fibroblast growth factor-23 in secondary hyperparathyroidism. Kidney Int. 2011;79(1):112‐119. 20861820 10.1038/ki.2010.352

[318] Li XH, Feng L, Yang ZH, Liao YH. Effect of active vitamin D on cardiovascular outcomes in predialysis chronic kidney diseases: a systematic review and meta-analysis. Nephrology. 2015;20(10):706‐714. 25963841 10.1111/nep.12505

[319] Cozzolino M, Bernard L, Csomor PA. Active vitamin D increases the risk of hypercalcaemia in non-dialysis chronic kidney disease patients with secondary hyperparathyroidism: a systematic review and meta-analysis. Clin Kidney J. 2021;14(11):2437‐2443. 34754440 10.1093/ckj/sfab091 PMC8573010

[320] Xu L, Wan X, Huang Z, et al Impact of vitamin D on chronic kidney diseases in non-dialysis patients: a meta-analysis of randomized controlled trials. PLoS One. 2013;8(4):e61387. 23626678 10.1371/journal.pone.0061387 PMC3634086

[321] Liu Y, Yang Q, Chen G, Zhou T. A systematic review and meta-analysis of efficacy and safety of calcimimetic agents in the treatment of secondary hyperparathyroidism in patients with chronic kidney disease. Curr Pharm Des. 2022;28(40):3289‐3304. 36305135 10.2174/1381612829666221027110656

[322] Yajima A, Akizawa T, Tsukamoto Y, Kurihara S, Ito A. Impact of cinacalcet hydrochloride on bone histology in patients with secondary hyperparathyroidism. Ther Apher Dial. 2008;12(s1):S38‐S43. 19032526 10.1111/j.1744-9987.2008.00630.x

[323] Yano S, Suzuki K, Sumi M, et al Bone metabolism after cinacalcet administration in patients with secondary hyperparathyroidism. J Bone Miner Metab. 2010;28(1):49‐54. 19548062 10.1007/s00774-009-0102-6

[324] Lindberg JS, Culleton B, Wong G, et al Cinacalcet HCl, an oral calcimimetic agent for the treatment of secondary hyperparathyroidism in hemodialysis and peritoneal dialysis: a randomized, double-blind, multicenter study. J Am Soc Nephrol. 2005;16(3):800‐807. 15689407 10.1681/ASN.2004060512

[325] Behets GJ, Spasovski G, Sterling LR, et al Bone histomorphometry before and after long-term treatment with cinacalcet in dialysis patients with secondary hyperparathyroidism. Kidney Int. 2015;87(4):846‐856. 25337774 10.1038/ki.2014.349 PMC4382689

[326] Effect of cinacalcet on cardiovascular disease in patients undergoing dialysis. N Engl J Med. 2012;367(26):2482‐2494. 23121374 10.1056/NEJMoa1205624

[327] Moe SM, Abdalla S, Chertow GM, et al Effects of cinacalcet on fracture events in patients receiving hemodialysis: the EVOLVE trial. J Am Soc Nephrol. 2015;26(6):1466‐1475. 25505257 10.1681/ASN.2014040414 PMC4446874

[328] Tsuruta Y, Okano K, Kikuchi K, Tsuruta Y, Akiba T, Nitta K. Effects of cinacalcet on bone mineral density and bone markers in hemodialysis patients with secondary hyperparathyroidism. Clin Exp Nephrol. 2013;17(1):120‐126. 22833360 10.1007/s10157-012-0665-8

[329] Charytan C, Coburn JW, Chonchol M, et al Cinacalcet hydrochloride is an effective treatment for secondary hyperparathyroidism in patients with CKD not receiving dialysis. Am J Kidney Dis. 2005;46(1):58‐67. 15983958 10.1053/j.ajkd.2005.04.013

[330] Alpay N, Yıldız A. Effects of cinacalcet on post-transplantation hypercalcemia and hyperparathyroidism in adult kidney transplant patients: a single-center experience. Cureus. 2023;15(3):e36248. 37069889 10.7759/cureus.36248 PMC10105616

[331] Arenas MD, Rodelo-Haad C, de Mier MVPR, Rodriguez M. Control of hyperparathyroidism with the intravenous calcimimetic etelcalcetide in dialysis patients adherent and non-adherent to oral calcimimetics. Clin Kidney J. 2021;14(3):840‐846. 33777366 10.1093/ckj/sfaa005 PMC7986320

[332] Blair HA . Etelcalcetide: first global approval. Drugs. 2016;76(18):1787‐1792. 27900648 10.1007/s40265-016-0671-3

[333] Martin KJ, Pickthorn K, Huang S, et al AMG 416 (velcalcetide) is a novel peptide for the treatment of secondary hyperparathyroidism in a single-dose study in hemodialysis patients. Kidney Int. 2014;85(1):191‐197. 23903371 10.1038/ki.2013.289

[334] Cunningham J, Block GA, Chertow GM, et al Etelcalcetide is effective at all levels of severity of secondary hyperparathyroidism in hemodialysis patients. Kidney Int Rep. 2019;4(7):987‐994. 31317120 10.1016/j.ekir.2019.04.010 PMC6611952

[335] Shigematsu T, Fukagawa M, Yokoyama K, et al Effects of the intravenous calcimimetic etelcalcetide on bone turnover and Serum fibroblast growth factor 23: post hoc analysis of an open-label study. Clin Ther. 2018;40(12):2099‐2111. 30473399 10.1016/j.clinthera.2018.10.016

[336] Karaboyas A, Muenz D, Fuller DS, et al Etelcalcetide utilization, dosing titration, and chronic kidney disease–mineral and bone disease (CKD-MBD) marker responses in US hemodialysis patients. Am J Kidney Dis. 2022;79(3):362‐373. 34273436 10.1053/j.ajkd.2021.05.020

[337] Zhu Y, Ou J, Liu X, Xie Z. Etelcalcetide versus placebo for secondary hyperparathyroidism in patients receiving hemodialysis: a meta-analysis of randomized controlled trials. Clin Nephrol. 2020;94(4):173‐180. 32729818 10.5414/CN110110

[338] Massimetti C, Tondo M, Feriozzi S. [Long-term efficacy and safety of etelcalcetide in hemodialysis patients with severe secondary hyperparathyroidism]. G Ital Nefrol. 2020;37(5):2020-vol5. 33026204

[339] Eidman KE, Wetmore JB. Treatment of secondary hyperparathyroidism: how do cinacalcet and etelcalcetide differ? Semin Dial. 2018;31(5):440‐444. 30009474 10.1111/sdi.12734

[340] Block GA, Bushinsky DA, Cheng S, et al Effect of etelcalcetide vs cinacalcet on Serum parathyroid hormone in patients receiving hemodialysis with secondary hyperparathyroidism. JAMA. 2017;317(2):156. 28097356 10.1001/jama.2016.19468

[341] Block GA, Chertow GM, Sullivan JT, et al An integrated analysis of safety and tolerability of etelcalcetide in patients receiving hemodialysis with secondary hyperparathyroidism. PLoS One. 2019;14(3):e0213774. 30875390 10.1371/journal.pone.0213774 PMC6420005

[342] Li X, Yu L, Asuncion F, et al Etelcalcetide (AMG 416), a peptide agonist of the calcium-sensing receptor, preserved cortical bone structure and bone strength in subtotal nephrectomized rats with established secondary hyperparathyroidism. Bone. 2017;105:163‐172. 28867373 10.1016/j.bone.2017.08.026

[343] Pereira LAL, Meng C, Amoedo MAG, et al Etelcalcetide controls secondary hyperparathyroidism and raises sclerostin levels in hemodialysis patients previously uncontrolled with cinacalcet. Nefrología (Engl Ed). 2022;43(2):197‐203. 36437202 10.1016/j.nefroe.2022.11.014

[344] Khairallah P, Cherasard J, Sung J, et al Changes in bone quality after treatment with etelcalcetide. Clin J Am Soc Nephrol. 2023;18(11):1456‐1465. 37574661 10.2215/CJN.0000000000000254 PMC10637456

[345] Kawata T, Tokunaga S, Murai M, et al A novel calcimimetic agent, evocalcet (MT-4580/KHK7580), suppresses the parathyroid cell function with little effect on the gastrointestinal tract or CYP isozymes in vivo and in vitro. PLoS One. 2018;13(4):e0195316. 29614098 10.1371/journal.pone.0195316 PMC5882164

[346] Akizawa T, Shimazaki R, Shiramoto M, Fukagawa M. Pharmacokinetics, pharmacodynamics, and safety of the novel calcimimetic agent evocalcet in healthy Japanese subjects: first-in-human phase I study. Clin Drug Investig. 2018;38(10):945‐954. 10.1007/s40261-018-0687-4 PMC6182462 30168004

[347] Akizawa T, Shimazaki R, Fukagawa M. Phase 2b study of evocalcet (KHK7580), a novel calcimimetic, in Japanese patients with secondary hyperparathyroidism undergoing hemodialysis: a randomized, double-blind, placebo-controlled, dose-finding study. PLoS One. 2018;13(10):e0204896. 30379826 10.1371/journal.pone.0204896 PMC6209414

[348] Tsuruya K, Shimazaki R, Fukagawa M, et al Efficacy and safety of evocalcet in Japanese peritoneal dialysis patients. Clin Exp Nephrol. 2019;23(6):739‐748. 30955188 10.1007/s10157-019-01692-y PMC6586709

[349] Yokoyama K, Shimazaki R, Fukagawa M, Akizawa T. Long-term efficacy and safety of evocalcet in Japanese patients with secondary hyperparathyroidism receiving hemodialysis. Sci Rep. 2019;9(1):6410. 31015494 10.1038/s41598-019-42017-z PMC6478860

[350] Toussaint ND, Elder GJ, Kerr PG. Bisphosphonates in chronic kidney disease; balancing potential benefits and adverse effects on bone and soft tissue. Clin J Am Soc Nephrol. 2009;4(1):221‐233. 18987295 10.2215/CJN.02550508

[351] Lin JH . Bisphosphonates: a review of their pharmacokinetic properties. Bone. 1996;18(2):75‐85. 8833200 10.1016/8756-3282(95)00445-9

[352] Papapoulos SE, Cremers SCLM. Prolonged bisphosphonate release after treatment in children. N Engl J Med. 2007;356(10):1075‐1076. 10.1056/NEJMc062792 17347467

[353] Damasiewicz MJ, Nickolas TL. Bisphosphonate therapy in CKD. Curr Opin Nephrol Hypertens. 2020;29(2):221‐226. 31833938 10.1097/MNH.0000000000000585 PMC9341147

[354] Pfister T, Atzpodien E, Bohrmann B, Bauss F. Acute renal effects of intravenous bisphosphonates in the rat. Basic Clin Pharmacol Toxicol. 2005;97(6):374‐381. 16364053 10.1111/j.1742-7843.2005.pto_160.x

[355] Markowitz GS, Fine PL, Stack JI, et al Toxic acute tubular necrosis following treatment with zoledronate (zometa). Kidney Int. 2003;64(1):281‐289. 12787420 10.1046/j.1523-1755.2003.00071.x

[356] Banerjee D, Asif A, Striker L, Preston RA, Bourgoignie JJ, Roth D. Short-term, high-dose pamidronate-induced acute tubular necrosis: the postulated mechanisms of bisphosphonate nephrotoxicity. Am J Kidney Dis. 2003;41(5):e18.1‐e18.6. 12778436

[357] Desikan R, Veksler Y, Raza S, et al Nephrotic proteinuria associated with high-dose pamidronate in multiple myeloma. Br J Haematol. 2002;119(2):496‐499. 12406092 10.1046/j.1365-2141.2002.03826.x

[358] MARKOWITZ GS, APPEL GB, FINE PL, et al Collapsing focal segmental glomerulosclerosis following treatment with high-dose pamidronate. J Am Soc Nephrol. 2001;12(6):1164‐1172. 11373339 10.1681/ASN.V1261164

[359] Torregrosa JV, Ramos AM. [Use of bisphosphonates in chronic kidney disease]. Nefrologia (Engl Ed). 2010;30(3):288‐296. 10.3265/Nefrologia.pre2010.Apr.10320 20514097

[360] Amerling R, Harbord NB, Pullman J, Feinfeld DA. Bisphosphonate use in chronic kidney disease: association with adynamic bone disease in a bone histology series. Blood Purif. 2010;29(3):293‐299. 20090316 10.1159/000276666

[361] Swallow EA, Aref MW, Metzger CE, et al Skeletal levels of bisphosphonate in the setting of chronic kidney disease are independent of remodeling rate and lower with fractionated dosing. Bone. 2019;127:419‐426. 31299384 10.1016/j.bone.2019.07.007 PMC6708715

[362] Miller PD, Roux C, Boonen S, Barton IP, Dunlap LE, Burgio DE. Safety and efficacy of risedronate in patients with age-related reduced renal function as estimated by the cockcroft and gault method: a pooled analysis of nine clinical trials. J Bone Miner Res. 2005;20(12):2105‐2115. 16294264 10.1359/JBMR.050817

[363] Jamal SA, Bauer DC, Ensrud KE, et al Alendronate treatment in women with normal to severely impaired renal function: an analysis of the fracture intervention trial. J Bone Miner Res. 2007;22(4):503‐508. 17243862 10.1359/jbmr.070112

[364] Wilson LM, Rebholz CM, Jirru E, et al Benefits and Harms of osteoporosis medications in patients with chronic kidney disease. Ann Intern Med. 2017;166(9):649. 28395318 10.7326/M16-2752

[365] Shigematsu T, Muraoka R, Sugimoto T, Nishizawa Y. Risedronate therapy in patients with mild-to-moderate chronic kidney disease with osteoporosis: post-hoc analysis of data from the risedronate phase III clinical trials. BMC Nephrol. 2017;18(1):66. 28201994 10.1186/s12882-017-0478-9 PMC5311729

[366] Sugimoto T, Inoue D, Maehara M, Oikawa I, Shigematsu T, Nishizawa Y. Efficacy and safety of once-monthly risedronate in osteoporosis subjects with mild-to-moderate chronic kidney disease: a post hoc subgroup analysis of a phase III trial in Japan. J Bone Miner Metab. 2019;37(4):730‐740. 30523414 10.1007/s00774-018-0977-1

[367] Torregrosa JV, Moreno A, Mas M, Ybarra J, Fuster D. Usefulness of pamidronate in severe secondary hyperparathyroidism in patients undergoing hemodialysis. Kidney Int. 2003;63:S88‐S90. 10.1046/j.1523-1755.63.s85.21.x 12753274

[368] Whitlock R, MacDonald K, Tangri N, Walsh M, Collister D. The efficacy and safety of bisphosphonate therapy for osteopenia/osteoporosis in patients with chronic kidney disease: a systematic review and individual patient-level meta-analysis of placebo-controlled randomized trials. Can J Kidney Health Dis. 2024;11:20543581241283523. 39381071 10.1177/20543581241283523 PMC11459530

[369] Wetmore JB, Benet LZ, Kleinstuck D, Frassetto L. Effects of short-term alendronate on bone mineral density in haemodialysis patients. Nephrology(Carlton). 2005;10(4):393‐399. 16109088 10.1111/j.1440-1797.2005.00436.x

[370] Yamamoto S, Suzuki A, Sasaki H, et al Oral alendronate can suppress bone turnover but not fracture in kidney transplantation recipients with hyperparathyroidism and chronic kidney disease. J Bone Miner Metab. 2013;31(1):116‐122. 23076292 10.1007/s00774-012-0391-z

[371] Tillmann FP, Schmitz M, Jäger M, Krauspe R, Rump LC. Ibandronate in stable renal transplant recipients with low bone mineral density on long-term follow-up. Int Urol Nephrol. 2016;48(2):279‐286. 26498632 10.1007/s11255-015-1133-7

[372] McKee H, Ioannidis G, Lau A, et al Comparison of the clinical effectiveness and safety between the use of denosumab vs bisphosphonates in renal transplant patients. Osteoporos Int. 2020;31(5):973‐980. 31900542 10.1007/s00198-019-05267-1

[373] Jamal SA, Ljunggren Ö, Stehman-Breen C, et al Effects of denosumab on fracture and bone mineral density by level of kidney function. J Bone Miner Res. 2011;26(8):1829‐1835. 21491487 10.1002/jbmr.403

[374] Broadwell A, Chines A, Ebeling PR, et al Denosumab safety and efficacy among participants in the FREEDOM extension study with mild to moderate chronic kidney disease. J Clin Endocrinol Metab. 2021;106(2):397‐409. 33211870 10.1210/clinem/dgaa851 PMC7823314

[375] Block GA, Bone HG, Fang L, Lee E, Padhi D. A single-dose study of denosumab in patients with various degrees of renal impairment. J Bone Miner Res. 2012;27(7):1471‐1479. 22461041 10.1002/jbmr.1613 PMC3505375

[376] Simonini M, Bologna A, Vezzoli G. Is denosumab an efficient and safe drug for osteoporosis in dialysis patients? Considerations and state of the art about its use in this setting. Int Urol Nephrol. 2024;56(10):3285‐3293. 38856936 10.1007/s11255-024-04110-9

[377] Ominsky MS, Libanati C, Niu QT, et al Sustained modeling-based bone formation during adulthood in cynomolgus monkeys may contribute to continuous BMD gains with denosumab. J Bone Miner Res. 2015;30(7):1280‐1289. 25684625 10.1002/jbmr.2480

[378] Iseri K, Mizobuchi M, Winzenrieth R, et al Long-term effect of denosumab on bone disease in patients with CKD. Clin J Am Soc Nephrol. 2023;18(9):1195‐1203. doi:10.2215/CJN.0000000000000213 37314764 PMC10564351

[379] Chen CL, Chen NC, Hsu CY, et al An open-label, prospective pilot clinical study of denosumab for severe hyperparathyroidism in patients with low bone mass undergoing dialysis. J Clin Endocrinol Metab. 2014;99(7):2426‐2432. 24670088 10.1210/jc.2014-1154

[380] Farinola N, Kanjanapan Y. Denosumab-induced hypocalcaemia in high bone turnover states of malignancy and secondary hyperparathyroidism from renal failure. Intern Med J. 2013;43(11):1243‐1246. 24237647 10.1111/imj.12283

[381] Wada Y, Iyoda M, Iseri K, et al Combination therapy of denosumab and calcitriol for a renal transplant recipient with severe bone loss due to therapy-resistant hyperparathyroidism. Tohoku J Exp Med. 2016;238(3):205‐212. 26947314 10.1620/tjem.238.205

[382] Ishani A, Blackwell T, Jamal SA, Cummings SR, Ensrud KE. The effect of raloxifene treatment in postmenopausal women with CKD. J Am Soc Nephrol. 2008;19(7):1430‐1438. 18400939 10.1681/ASN.2007050555 PMC2440292

[383] Melamed ML, Blackwell T, Neugarten J, et al Raloxifene, a selective estrogen receptor modulator, is renoprotective: a post-hoc analysis. Kidney Int. 2011;79(2):241‐249. 20927038 10.1038/ki.2010.378

[384] Hernández E, Valera R, Alonzo E, et al Effects of raloxifene on bone metabolism and serum lipids in postmenopausal women on chronic hemodialysis. Kidney Int. 2003;63(6):2269‐2274. 12753317 10.1046/j.1523-1755.2003.00005.x

[385] Ma HY, Chen S, Lu LL, Gong W, Zhang AH. Raloxifene in the treatment of osteoporosis in postmenopausal women with End-stage renal disease: a systematic review and meta-analysis. Horm Metab Res. 2021;53(11):730‐737. 34740274 10.1055/a-1655-4362

[386] Haghverdi F, Farbodara T, Mortaji S, Soltani P, Saidi N. Effect of raloxifene on parathyroid hormone in osteopenic and osteoporotic postmenopausal women with chronic kidney disease stage 5. Iran J Kidney Dis. 2014;8(6):461‐466. 25362221

[387] Neer RM, Arnaud CD, Zanchetta JR, et al Effect of parathyroid hormone (1–34) on fractures and bone mineral density in postmenopausal women with osteoporosis. N Engl J Med. 2001;344(19):1434‐1441. 11346808 10.1056/NEJM200105103441904

[388] Miller PD, Schwartz EN, Chen P, Misurski DA, Krege JH. Teriparatide in postmenopausal women with osteoporosis and mild or moderate renal impairment. Osteoporos Int. 2007;18(1):59‐68. 17013567 10.1007/s00198-006-0189-8

[389] Nishikawa A, Yoshiki F, Taketsuna M, Kajimoto K, Enomoto H. Safety and effectiveness of daily teriparatide for osteoporosis in patients with severe stages of chronic kidney disease: post hoc analysis of a postmarketing observational study. Clin Interv Aging. 2016;11:1653‐1659. 27895472 10.2147/CIA.S120175 PMC5117886

[390] Takeuchi Y, Tanaka S, Kuroda T, Hagino H, Mori S, Soen S. Association between renal function and fracture incidence during treatment with teriparatide or alendronate: an exploratory subgroup analysis of the Japanese osteoporosis intervention trial-05. Osteoporos Int. 2024;35(12):2175‐2182. 39343826 10.1007/s00198-024-07260-9 PMC11579086

[391] Igarashi S, Kasukawa Y, Nozaka K, et al Teriparatide and etelcalcetide improve bone, fibrosis, and fat parameters in chronic kidney disease model rats. Osteoporos Sarcopenia. 2023;9(4):121‐130. 38374820 10.1016/j.afos.2023.11.002 PMC10874735

[392] Ota M, Takahata M, Shimizu T, et al Efficacy and safety of osteoporosis medications in a rat model of late-stage chronic kidney disease accompanied by secondary hyperparathyroidism and hyperphosphatemia. Osteoporos Int. 2017;28(4):1481‐1490. 27933339 10.1007/s00198-016-3861-7

[393] Yamamoto J, Nakazawa D, Nishio S, et al Impact of weekly teriparatide on the bone and mineral metabolism in hemodialysis patients with relatively low Serum parathyroid hormone: a pilot study. Ther Apher Dial. 2020;24(2):146‐153. 31210004 10.1111/1744-9987.12867

[394] Palcu P, Dion N, Ste-Marie LG, et al Teriparatide and bone turnover and formation in a hemodialysis patient with low-turnover bone disease: a case report. Am J Kidney Dis. 2015;65(6):933‐936. 25843705 10.1053/j.ajkd.2015.01.025

[395] van Lierop AH, Appelman-Dijkstra NM, Papapoulos SE. Sclerostin deficiency in humans. Bone. 2017;96:51‐62. 27742500 10.1016/j.bone.2016.10.010

[396] Prather C, Adams E, Zentgraf W. Romosozumab: a first-in-class sclerostin inhibitor for osteoporosis. Am J Health Syst Pharm. 2020;77(23):1949‐1956. 32880646 10.1093/ajhp/zxaa285

[397] McClung MR . Clinical utility of anti-sclerostin antibodies. Bone. 2017;96:3‐7. 28115281 10.1016/j.bone.2016.12.012

[398] Miller PD, Adachi JD, Albergaria B, et al Efficacy and safety of romosozumab among postmenopausal women with osteoporosis and mild-to-moderate chronic kidney disease. J Bone Miner Res. 2022;37(8):1437‐1445. 35466448 10.1002/jbmr.4563 PMC9544335

[399] Sato M, Inaba M, Yamada S, Emoto M, Ohno Y, Tsujimoto Y. Efficacy of romosozumab in patients with osteoporosis on maintenance hemodialysis in Japan; an observational study. J Bone Miner Metab. 2021;39(6):1082‐1090. 34324082 10.1007/s00774-021-01253-y

[400] Suzuki T, Mizobuchi M, Yoshida S, et al Romosozumab successfully regulated progressive osteoporosis in a patient with autosomal dominant polycystic kidney disease undergoing hemodialysis. Osteoporos Int. 2022;33(12):2649‐2652. 35980440 10.1007/s00198-022-06534-4

[401] Ogata M, Ushimaru S, Fujishima R, Sumi H, Shiizaki K, Tominaga N. Romosozumab improves low bone mineral density in a postmenopausal woman undergoing chronic hemodialysis and treated with a calcium-sensing receptor agonist. Bone Rep. 2022;17:101639. 36438716 10.1016/j.bonr.2022.101639 PMC9685381

[402] Saito T, Mizobuchi M, Kato T, et al One-year Romosozumab treatment followed by one-year denosumab treatment for osteoporosis in patients on hemodialysis: an observational study. Calcif Tissue Int. 2022;112(1):34‐44. 36287217 10.1007/s00223-022-01031-6

[403] Rodríguez-Ortiz ME, Pendón-Ruiz de Mier MV, Rodríguez M. Parathyroidectomy in dialysis patients: indications, methods, and consequences. Semin Dial. 2019;32(5):444‐451. 30656752 10.1111/sdi.12772

[404] Lu KC, Ma WY, Yu JC, Wu CC, Chu P. Bone turnover markers predict changes in bone mineral density after parathyroidectomy in patients with renal hyperparathyroidism. Clin Endocrinol (Oxf). 2012;76(5):634‐642. 22007930 10.1111/j.1365-2265.2011.04265.x

[405] Yajima A, Ogawa Y, Takahashi HE, Tominaga Y, Inou T, Otsubo O. Changes of bone remodeling immediately after parathyroidectomy for secondary hyperparathyroidism. Am J Kidney Dis. 2003;42(4):729‐738. 14520623 10.1016/s0272-6386(03)00909-0

[406] Schneider R, Steinmetz C, Karakas E, Bartsch DK, Schlosser K. Influence of parathyroidectomy on bone metabolism and bone pain in patients with secondary hyperparathyroidism. Eur Surg Res. 2018;59(1-2):35‐47. 29393259 10.1159/000486172

[407] Ge Y, Yang G, Wang N, et al Bone metabolism markers and hungry bone syndrome after parathyroidectomy in dialysis patients with secondary hyperparathyroidism. Int Urol Nephrol. 2019;51(8):1443‐1449. 31264087 10.1007/s11255-019-02217-y

[408] Ma L, Zhao S, Li Z. Effects of parathyroidectomy on bone metabolism in haemodialysis patients with secondary hyperparathyroidism. Scand J Clin Lab Invest. 2017;77(7):527‐534. 28741963 10.1080/00365513.2017.1354256

[409] Siqueira Fd, Oliveira Kd, Dominguez WV, et al Effect of parathyroidectomy on bone tissue biomarkers and body composition in patients with chronic kidney disease and secondary hyperparathyroidism. Eur J Clin Nutr. 2021;75(7):1126‐1133. 33462459 10.1038/s41430-020-00829-7

[410] Wang AYM, Tang TK, Yau YY, Lo WK. Impact of parathyroidectomy versus oral cinacalcet on bone mineral density in patients on peritoneal dialysis with advanced secondary hyperparathyroidism: the PROCEED pilot randomized trial. Am J Kidney Dis. 2024;83(4):456‐466.e1. 38040277 10.1053/j.ajkd.2023.10.007

[411] Bellorin-Font E, Rojas E, Martin KJ. Bone disease in chronic kidney disease and kidney transplant. Nutrients. 2022;15(1):167. 36615824 10.3390/nu15010167 PMC9824497

[412] Sun L, Wang Z, Zheng M, et al Mineral and bone disorder after kidney transplantation: a single-center cohort study. Ren Fail. 2023;45(1):2210231. 37183797 10.1080/0886022X.2023.2210231 PMC10187110

[413] Sprague SM, Belozeroff V, Danese MD, Martin LP, Olgaard K. Abnormal bone and mineral metabolism in kidney transplant patients—a review. Am J Nephrol. 2008;28(2):246‐253. 17989497 10.1159/000110875

[414] Ferreira AC, Mendes M, Silva C, et al Improvement of mineral and bone disorders after renal transplantation. Transplantation. 2022;106(5):e251‐e261. 35266925 10.1097/TP.0000000000004099 PMC9038238

[415] Jia L, Chao S, Yang Q, et al The comprehensive incidence and risk factors of fracture in kidney transplant recipients: a meta-analysis. Nephrology. 2024;29(9):588‐599. 38689467 10.1111/nep.14301

[416] Tsai HL, Lin TC, Lin NC, Yang HH, Chang JW. Risk factors for fractures in renal transplantation: a population-based cohort study. Am J Nephrol. 2023;54(11-12):498‐507. 37783206 10.1159/000533125

[417] Eum SH, Kim DW, Lee JH, et al Time-varying risk factors for incident fractures in kidney transplant recipients: a nationwide cohort study in South Korea. J Clin Med. 2023;12(6):2337. 36983337 10.3390/jcm12062337 PMC10058856

[418] Iyer SP, Nikkel LE, Nishiyama KK, et al Kidney transplantation with early corticosteroid withdrawal: paradoxical effects at the central and peripheral Skeleton. J Am Soc Nephrol. 2014;25(6):1331‐1341. 24511131 10.1681/ASN.2013080851 PMC4033378

[419] Chou FF, Hsieh KC, Chen YT, Lee CT. Parathyroidectomy followed by kidney transplantation can improve bone mineral density in patients with secondary hyperparathyroidism. Transplantation. 2008;86(4):554‐557. 18724225 10.1097/TP.0b013e3181814b00

[420] Clarke BL, Brown EM, Collins MT, et al Epidemiology and diagnosis of hypoparathyroidism. J Clin Endocrinol Metab. 2016;101(6):2284‐2299. 26943720 10.1210/jc.2015-3908 PMC5393595

[421] Bilezikian JP . Hypoparathyroidism. J Clin Endocrinol Metab. 2020;105(6):1722‐1736. 32322899 10.1210/clinem/dgaa113 PMC7176479

[422] Clarke BL . Bone disease in hypoparathyroidism. Arq Bras Endocrinol Metabol. 2014;58(5):545‐552. 25166046 10.1590/0004-2730000003399

[423] Pepe J, Colangelo L, Biamonte F, et al Diagnosis and management of hypocalcemia. Endocrine. 2020;69(3):485‐495. 32367335 10.1007/s12020-020-02324-2

[424] Cianferotti L . Classification of hypoparathyroid disorders. Front Horm Res. 2019;51:127‐138. 30641517 10.1159/000491043

[425] Gafni RI, Collins MT. Hypoparathyroidism. N Engl J Med. 2019;380(18):1738‐1747. 31042826 10.1056/NEJMcp1800213

[426] Cusano NE, Rubin MR, Williams JM, et al Changes in skeletal microstructure through four continuous years of rhPTH(1–84) therapy in hypoparathyroidism. J Bone Miner Res. 2020;35(7):1274‐1281. 32155287 10.1002/jbmr.4005 PMC7363559

[427] Harsløf T, Rolighed L, Rejnmark L. Huge variations in definition and reported incidence of postsurgical hypoparathyroidism: a systematic review. Endocrine. 2019;64(1):176‐183. 30788669 10.1007/s12020-019-01858-4

[428] Asari R . Hypoparathyroidism after total thyroidectomy. Archives of Surgery. 2008;143(2):132. 18283137 10.1001/archsurg.2007.55

[429] Nagel K, Hendricks A, Lenschow C, et al Definition and diagnosis of postsurgical hypoparathyroidism after thyroid surgery: meta-analysis. BJS Open. 2022;6(5):zrac102. 36050906 10.1093/bjsopen/zrac102 PMC9437325

[430] Khan AA, Bilezikian JP, Brandi ML, et al Evaluation and management of hypoparathyroidism summary statement and guidelines from the second international workshop. J Bone Miner Res. 2022;37(12):2568‐2585. 36054621 10.1002/jbmr.4691

[431] Simões CA, Costa MK, Comerlato LB, et al A case of “late” postsurgical hypoparathyroidism. Case Rep Endocrinol. 2017;2017:3962951. 28642829 10.1155/2017/3962951 PMC5469994

[432] Halperin I, Nubiola A, Vendrell J, Vilardell E. Late-onset hypocalcemia appearing years after thyroid surgery. J Endocrinol Invest. 1989;12(6):419‐420. 2768762 10.1007/BF03350718

[433] Kakava K, Tournis S, Makris K, et al Identification of patients at high risk for postsurgical hypoparathyroidism. In Vivo (Brooklyn). 2020;34(5):2973‐2980. 10.21873/invivo.12128 PMC7652524 32871840

[434] Melikyan AA, Menkov AV. Postoperative hypoparathyroidism: prognosis, prevention, and treatment (review). Sovrem Tekhnologii Med. 2020;12(2):101. 34513060 10.17691/stm2020.12.2.13 PMC8353683

[435] Langdahl BL, Mortensen L, Vesterby A, Eriksen EF, Charles P. Bone histomorphometry in hypoparathyroid patients treated with vitamin D. Bone. 1996;18(2):103‐108. 8833203 10.1016/8756-3282(95)00443-2

[436] Rubin MR, Dempster DW, Zhou H, et al Dynamic and structural properties of the Skeleton in hypoparathyroidism. J Bone Miner Res. 2008;23(12):2018‐2024. 18684087 10.1359/JBMR.080803 PMC2686925

[437] Rubin MR, Dempster DW, Kohler T, et al Three dimensional cancellous bone structure in hypoparathyroidism. Bone. 2010;46(1):190‐195. 19782782 10.1016/j.bone.2009.09.020 PMC2818211

[438] Abugassa S, Nordenström J, Eriksson S, Sjödén G. Bone mineral density in patients with chronic hypoparathyroidism. J Clin Endocrinol Metab. 1993;76(6):1617‐1621. 8501170 10.1210/jcem.76.6.8501170

[439] Cusano NE, Nishiyama KK, Zhang C, et al Noninvasive assessment of skeletal microstructure and estimated bone strength in hypoparathyroidism. J Bone Miner Res. 2016;31(2):308‐316. 26234545 10.1002/jbmr.2609 PMC4832602

[440] Silva BC, Rubin MR, Cusano NE, Bilezikian JP. Bone imaging in hypoparathyroidism. Osteoporos Int. 2017;28(2):463‐471. 27577725 10.1007/s00198-016-3750-0

[441] Chan FKW, Tiu SC, Choi KL, Choi CH, Kong APS, Shek CC. Increased bone mineral density in patients with chronic hypoparathyroidism. J Clin Endocrinol Metab. 2003;88(7):3155‐3159. 12843159 10.1210/jc.2002-021388

[442] Chen Q, Kaji H, Iu MF, et al Effects of an excess and a deficiency of endogenous parathyroid hormone on volumetric bone mineral density and bone geometry determined by peripheral quantitative computed tomography in female subjects. J Clin Endocrinol Metab. 2003;88(10):4655‐4658. 14557436 10.1210/jc.2003-030470

[443] Starr JR, Tabacco G, Majeed R, Omeragic B, Bandeira L, Rubin MR. PTH and bone material strength in hypoparathyroidism as measured by impact microindentation. Osteoporos Int. 2020;31(2):327‐333. 31720712 10.1007/s00198-019-05177-2

[444] Underbjerg L, Sikjaer T, Mosekilde L, Rejnmark L. The epidemiology of nonsurgical hypoparathyroidism in Denmark: a nationwide case finding study. J Bone Miner Res. 2015;30(9):1738‐1744. 25753591 10.1002/jbmr.2501

[445] Mendonça ML, Pereira FA, Nogueira-Barbosa MH, et al Increased vertebral morphometric fracture in patients with postsurgical hypoparathyroidism despite normal bone mineral density. BMC Endocr Disord. 2013;13(1):1. 23286605 10.1186/1472-6823-13-1 PMC3546901

[446] Cipriani C, Minisola S, Bilezikian JP, et al Vertebral fracture assessment in postmenopausal women with postsurgical hypoparathyroidism. J Clin Endocrinol Metab. 2021;106(5):1303‐1311. 33567075 10.1210/clinem/dgab076 PMC8063231

[447] Saha S, Mannar V, Kandasamy D, Sreenivas V, Goswami R. Vertebral fractures, trabecular bone score and their determinants in chronic hypoparathyroidism. J Endocrinol Invest. 2022;45(9):1777‐1786. 35585296 10.1007/s40618-022-01818-2

[448] Underbjerg L, Sikjaer T, Mosekilde L, Rejnmark L. Postsurgical hypoparathyroidism-risk of fractures, psychiatric diseases, cancer, cataract, and infections. J Bone Miner Res. 2014;29(11):2504‐2510. 24806578 10.1002/jbmr.2273

[449] Ahn SH, Lee YJ, Hong S, et al Risk of fractures in thyroid cancer patients with postoperative hypoparathyroidism: a nationwide cohort study in Korea. J Bone Miner Res. 2023;38(9):1268‐1277. 37338940 10.1002/jbmr.4871

[450] Underbjerg L, Malmstroem S, Sikjaer T, Rejnmark L. Bone Status among patients with nonsurgical hypoparathyroidism, autosomal dominant hypocalcaemia, and pseudohypoparathyroidism: a cohort study. J Bone Miner Res. 2018;33(3):467‐477. 29087612 10.1002/jbmr.3328

[451] Chawla H, Saha S, Kandaswamy D, Sharma R, Sreenivas V, Goswami R. Vertebral fractures and bone mineral density in patients with idiopathic hypoparathyroidism on long term follow-up. J Clin Endocrinol Metab. 2016;102(1):251‐258. doi:10.1210/jc.2016-3292 27813708

[452] Pal R, Bhadada SK, Mukherjee S, Banerjee M, Kumar A. Fracture risk in hypoparathyroidism: a systematic review and meta-analysis. Osteoporos Int. 2021;32(11):2145‐2153. 34021765 10.1007/s00198-021-05966-8

[453] Brandi ML, Bilezikian JP, Shoback D, et al Management of hypoparathyroidism: summary statement and guidelines. J Clin Endocrinol Metab. 2016;101(6):2273‐2283. 26943719 10.1210/jc.2015-3907

[454] Arlt W, Fremerey C, Callies F, et al Well-being, mood and calcium homeostasis in patients with hypoparathyroidism receiving standard treatment with calcium and vitamin D. Eur J Endocrinol. 2002;146(2):215‐222. 11834431 10.1530/eje.0.1460215

[455] Stamm B, Blaschke M, Wilken L, et al The influence of conventional treatment on symptoms and complaints in patients with chronic postsurgical hypoparathyroidism. JBMR Plus. 2022;6(2):e10586. 35229064 10.1002/jbm4.10586 PMC8861984

[456] Bilezikian JP, Brandi ML, Cusano NE, et al Management of hypoparathyroidism: present and future. J Clin Endocrinol Metab. 2016;101(6):2313‐2324. 26938200 10.1210/jc.2015-3910 PMC5393596

[457] Rubin MR, Sliney J, McMahon DJ, Silverberg SJ, Bilezikian JP. Therapy of hypoparathyroidism with intact parathyroid hormone. Osteoporos Int. 2010;21(11):1927‐1934. 20094706 10.1007/s00198-009-1149-x PMC2947814

[458] Sikjaer T, Rejnmark L, Rolighed L, Heickendorff L, Mosekilde L. The effect of adding PTH(1-84) to conventional treatment of hypoparathyroidism: a randomized, placebo-controlled study. J Bone Miner Res. 2011;26(10):2358‐2370. 21773992 10.1002/jbmr.470

[459] Cusano NE, Rubin MR, McMahon DJ, et al Therapy of hypoparathyroidism with PTH(1–84): a prospective four-year investigation of efficacy and safety. J Clin Endocrinol Metab. 2013;98(1):137‐144. 23162103 10.1210/jc.2012-2984 PMC3537109

[460] Rubin MR, Dempster DW, Sliney J JR, et al PTH(1-84) administration reverses abnormal bone-remodeling dynamics and structure in hypoparathyroidism. J Bone Miner Res. 2011;26(11):2727‐2736. 21735476 10.1002/jbmr.452 PMC4019384

[461] Sikjaer T, Rejnmark L, Thomsen JS, et al Changes in 3-dimensional bone structure indices in hypoparathyroid patients treated with PTH(1-84): a randomized controlled study. J Bone Miner Res. 2012;27(4):781‐788. 22161686 10.1002/jbmr.1493

[462] van Dijk Christiansen P, Sikjær T, Andreasen CM, et al Transitory activation and improved transition from erosion to formation within intracortical bone remodeling in hypoparathyroid patients treated with rhPTH (1–84). JBMR Plus. 2023;7(12):e10829. 38130746 10.1002/jbm4.10829 PMC10731115

[463] Orloff LA, Wiseman SM, Bernet VJ, et al American thyroid association statement on postoperative hypoparathyroidism: diagnosis, prevention, and management in adults. Thyroid. 2018;28(7):830‐841. 29848235 10.1089/thy.2017.0309

[464] Mannstadt M, Clarke BL, Vokes T, et al Efficacy and safety of recombinant human parathyroid hormone (1–84) in hypoparathyroidism (REPLACE): a double-blind, placebo-controlled, randomised, phase 3 study. Lancet Diabetes Endocrinol. 2013;1(4):275‐283. 24622413 10.1016/S2213-8587(13)70106-2

[465] Lakatos P, Bajnok L, Lagast H, Valkusz Z. An open-label extension study of parathyroid hormone rhpth(1-84) in adults with hypoparathyroidism. Endocr Pract. 2016;22(5):523‐532. 26684150 10.4158/EP15936.OR

[466] Mannstadt M, Clarke BL, Bilezikian JP, et al Safety and efficacy of 5 years of treatment with recombinant human parathyroid hormone in adults with hypoparathyroidism. J Clin Endocrinol Metab. 2019;104(11):5136‐5147. 31369089 10.1210/jc.2019-01010 PMC6760337

[467] Rubin MR, Zhou H, Cusano NE, et al The effects of long-term administration of rhPTH(1-84) in hypoparathyroidism by bone histomorphometry. J Bone Miner Res. 2018;33(11):1931‐1939. 29972871 10.1002/jbmr.3543 PMC6546298

[468] Agarwal S, McMahon DJ, Chen J, et al The clinical and skeletal effects of long-term therapy of hypoparathyroidism with rhPTH (1–84). J Bone Miner Res. 2023;38(4):480‐492. 36726204 10.1002/jbmr.4780 PMC10101915

[469] Puliani G, Hasenmajer V, Simonelli I, et al Safety and efficacy of PTH 1–34 and 1–84 therapy in chronic hypoparathyroidism: a meta-analysis of prospective trials. J Bone Miner Res. 2022;37(7):1233‐1250. 35485213 10.1002/jbmr.4566 PMC9545848

[470] Winer KK, Yanovski JA, Cutler GB. Synthetic human parathyroid hormone 1–34 vs calcitriol and calcium in the treatment of hypoparathyroidism. JAMA. 1996;276(8):631‐636. 8773636

[471] Winer KK, Yanovski JA, Sarani B, Cutler GB Jr. A randomized, cross-over trial of once-daily versus twice-daily parathyroid hormone 1–34 in treatment of hypoparathyroidism. J Clin Endocrinol Metab. 1998;83(10):3480‐3486. 9768650 10.1210/jcem.83.10.5185

[472] Winer KK, Sinaii N, Peterson D, Sainz B, Cutler GB. Effects of once *Versus* twice-daily parathyroid hormone 1–34 therapy in children with hypoparathyroidism. J Clin Endocrinol Metab. 2008;93(9):3389‐3395. 18492754 10.1210/jc.2007-2552 PMC2567852

[473] Winer KK, Ko CW, Reynolds JC, et al Long-term treatment of hypoparathyroidism: a randomized controlled study comparing parathyroid hormone-(1–34) *Versus* calcitriol and calcium. J Clin Endocrinol Metab. 2003;88(9):4214‐4220. 12970289 10.1210/jc.2002-021736

[474] Winer KK, Sinaii N, Reynolds J, Peterson D, Dowdy K, Cutler GB. Long-term treatment of 12 children with chronic hypoparathyroidism: a randomized trial comparing synthetic human parathyroid hormone 1-34 versus calcitriol and calcium. J Clin Endocrinol Metab. 2010;95(6):2680‐2688. 20392870 10.1210/jc.2009-2464 PMC2902068

[475] Santonati A, Palermo A, Maddaloni E, et al PTH(1–34) for surgical hypoparathyroidism: a prospective, open-label investigation of efficacy and quality of life. J Clin Endocrinol Metab. 2015;100(9):3590‐3597. 26196949 10.1210/jc.2015-1855

[476] Palermo A, Santonati A, Tabacco G, et al PTH(1–34) for surgical hypoparathyroidism: a 2-year prospective, open-label investigation of efficacy and quality of life. J Clin Endocrinol Metab. 2018;103(1):271‐280. 29099939 10.1210/jc.2017-01555

[477] Winer KK, Ye S, Ferré EMN, et al Therapy with PTH 1–34 or calcitriol and calcium in diverse etiologies of hypoparathyroidism over 27 years at a single tertiary care center. Bone. 2021;149:115977. 33932619 10.1016/j.bone.2021.115977

[478] Winer KK, Fulton KA, Albert PS, Cutler GB. Effects of pump versus twice-daily injection delivery of synthetic parathyroid hormone 1-34 in children with severe congenital hypoparathyroidism. J Pediatr. 2014;165(3):556‐563.e1. 24948345 10.1016/j.jpeds.2014.04.060 PMC4174419

[479] Haider A, Symczyk O, Couso Y, Cater T, Bryan S. Desperate times call for desperate measures: use of continuous subcutaneous 1-34 PTH infusion for postsurgical hypoparathyroidism. Case Rep Endocrinol. 2021;2021:5593653. 33777458 10.1155/2021/5593653 PMC7969095

[480] Gafni RI, Brahim JS, Andreopoulou P, et al Daily parathyroid hormone 1-34 replacement therapy for hypoparathyroidism induces marked changes in bone turnover and structure. J Bone Miner Res. 2012;27(8):1811‐1820. 22492501 10.1002/jbmr.1627 PMC3399961

[481] Yao L, Li J, Li M, et al Parathyroid hormone therapy for managing chronic hypoparathyroidism: a systematic review and meta-analysis. J Bone Miner Res. 2022;37(12):2654‐2662. 36385517 10.1002/jbmr.4676

[482] Charoenngam N, Bove-Fenderson E, Wong D, Cusano NE, Mannstadt M. Continuous subcutaneous delivery of rhPTH(1-84) and rhPTH(1-34) by pump in adults with hypoparathyroidism. J Endocr Soc. 2024;8(5):bvae053. 38562130 10.1210/jendso/bvae053 PMC10983071

[483] Holten-Andersen L, Pihl S, Rasmussen CE, et al Design and preclinical development of TransCon PTH, an investigational sustained-release PTH replacement therapy for hypoparathyroidism. J Bone Miner Res. 2019;34(11):2075‐2086. 31291476 10.1002/jbmr.3824

[484] Karpf DB, Pihl S, Mourya S, et al A randomized double-blind placebo-controlled first-in-human phase 1 trial of TransCon PTH in healthy adults. J Bone Miner Res. 2020;35(8):1430‐1440. 32212275 10.1002/jbmr.4016 PMC9328939

[485] Khan AA, Rejnmark L, Rubin M, et al PaTH forward: a randomized, double-blind, placebo-controlled phase 2 trial of TransCon PTH in adult hypoparathyroidism. J Clin Endocrinol Metab. 2022;107(1):e372‐e385. 34347093 10.1210/clinem/dgab577 PMC8684498

[486] Khan AA, Rubin MR, Schwarz P, et al Efficacy and safety of parathyroid hormone replacement with TransCon PTH in hypoparathyroidism: 26-week results from the phase 3 PaTHway trial. J Bone Miner Res. 2023;38(1):14‐25. 36271471 10.1002/jbmr.4726 PMC10099823

[487] Mantovani G, Bastepe M, Monk D, et al Diagnosis and management of pseudohypoparathyroidism and related disorders: first international consensus statement. Nat Rev Endocrinol. 2018;14(8):476‐500. 29959430 10.1038/s41574-018-0042-0 PMC6541219

[488] Mantovani G . Pseudohypoparathyroidism: diagnosis and treatment. J Clin Endocrinol Metab. 2011;96(10):3020‐3030. 21816789 10.1210/jc.2011-1048

[489] Hanna P, Grybek V, Perez de Nanclares G, et al Genetic and epigenetic defects at the GNAS locus lead to distinct patterns of skeletal growth but similar early-onset obesity. J Bone Miner Res. 2018;33(8):1480‐1488. 29693731 10.1002/jbmr.3450 PMC6105438

[490] Schlund M, Depeyre A, Kohler F, Nicot R, Ferri J. Cranio-maxillofacial and dental findings in Albright's hereditary osteodystrophy and pseudohypoparathyroidism. Cleft Palate Craniofac J. 2019;56(6):831‐836. 30497275 10.1177/1055665618814661

[491] Mantovani G, Bastepe M, Monk D, et al Recommendations for diagnosis and treatment of pseudohypoparathyroidism and related disorders: an updated practical tool for physicians and patients. Horm Res Paediatr. 2020;93(3):182‐196. 32756064 10.1159/000508985 PMC8140671

[492] Wang Y, Lu C, Chen X. Variable bone phenotypes in patients with pseudohypoparathyroidism. Curr Osteoporos Rep. 2023;21(3):311‐321. 37014531 10.1007/s11914-023-00787-6

[493] Duan X, Yang Y, Wang O, et al Bone turnover markers are associated with the PTH levels in different subtypes of pseudohypoparathyroidism type 1 patients. Clin Endocrinol (Oxf). 2021;95(2):277‐285. 33961300 10.1111/cen.14496

[494] Chu X, Zhu Y, Wang O, et al Bone mineral density and its serial changes are associated with PTH levels in pseudohypoparathyroidism type 1B patients. J Bone Miner Res. 2018;33(4):743‐752. 29240265 10.1002/jbmr.3360

[495] Kanatani M, Sugimoto T, Kaji H, Ikeda K, Chihara K. Skeletal responsiveness to parathyroid hormone in pseudohypoparathyroidism. Eur J Endocrinol. 2001;144(3):263‐269. 11248746 10.1530/eje.0.1440263

[496] Sbrocchi AM, Rauch F, Lawson ML, et al Osteosclerosis in two brothers with autosomal dominant pseudohypoparathyroidism type 1b: bone histomorphometric analysis. Eur J Endocrinol. 2011;164(2):295‐301. 21062889 10.1530/EJE-10-0795 PMC3810006

[497] Chen X, Meng Y, Tang M, et al A paternally inherited non-sense variant c.424G > T (p.G142*) in the first exon of XLαs in an adult patient with hypophosphatemia and osteopetrosis. Clin Genet. 2020;97(5):712‐722. 32157680 10.1111/cge.13734

[498] Long DN, Levine MA, Germain-Lee EL. Bone mineral density in pseudohypoparathyroidism type 1a. J Clin Endocrinol Metab. 2010;95(9):4465‐4475. 20610593 10.1210/jc.2010-0498 PMC2936057

[499] Underbjerg L, Sikjaer T, Mosekilde L, Rejnmark L. Pseudohypoparathyroidism—epidemiology, mortality and risk of complications. Clin Endocrinol (Oxf). 2016;84(6):904‐911. 26387561 10.1111/cen.12948

[500] Tollin SR, Perlmutter S, Aloia JF. Serial changes in bone mineral density and bone turnover after correction of secondary hyperparathyroidism in a patient with pseudohypoparathyroidism type ib. J Bone Miner Res. 2000;15(7):1412‐1416. 10893692 10.1359/jbmr.2000.15.7.1412

[501] Neary NM, El-Maouche D, Hopkins R, Libutti SK, Moses AM, Weinstein LS. Development and treatment of tertiary hyperparathyroidism in patients with pseudohypoparathyroidism type 1B. J Clin Endocrinol Metab. 2012;97(9):3025‐3030. 22736772 10.1210/jc.2012-1655 PMC3431579

[502] Srivastava T, Krudys J, Mardis NJ, Sebestyen-VanSickle J, Alon US. Cinacalcet as adjunctive therapy in pseudohypoparathyroidism type 1b. Pediatric Nephrology. 2016;31(5):795‐800. 26628282 10.1007/s00467-015-3271-7

